# Boron Designer Enzyme with a Hybrid Catalytic Dyad

**DOI:** 10.1021/acscatal.4c06052

**Published:** 2024-12-03

**Authors:** Lars Longwitz, Marijn D. Kamer, Bart Brouwer, Andy-Mark W. H. Thunnissen, Gerard Roelfes

**Affiliations:** † Stratingh Institute for Chemistry, University of Groningen, Groningen 9747 AG, The Netherlands; ‡ Groningen Biomolecular Sciences and Biotechnology Institute, University of Groningen, Groningen 9747 AG, The Netherlands

**Keywords:** designer enzymes, boron catalysis, noncanonical amino acids, kinetic resolution, genetic incorporation

## Abstract

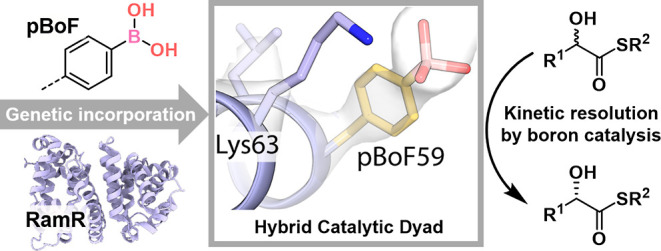

Genetically encoded noncanonical amino acids can introduce new-to-nature activation modes into enzymes. While these amino acids can act as catalysts on their own due to their inherent chemical properties, interactions with adjacent residues in an enzyme, such as those present in natural catalytic dyads or triads, unlock a higher potential for designer enzymes. We incorporated a boron-containing amino acid into the protein scaffold RamR to create an active enzyme for the kinetic resolution of α-hydroxythioesters. We found that a closely positioned lysine residue is crucial for the catalytic activity of the designer enzyme by forming a hybrid catalytic dyad with the boronic acid residue. The enzyme is capable of resolving differently substituted α-hydroxythioesters with good selectivities. High-resolution mass spectrometry, ^11^B NMR spectroscopy, and crystal structure analysis of the designer enzyme gave insight into the three steps of the mechanism (substrate binding, hydroxide transfer, product release). Mutations of a residue around the catalytic dyad led to a variant of the enzyme with 2-fold improvement of catalytic activity and selectivity.

## Introduction

The creation of enzymes to perform new-to-nature reactions can improve synthetic pathways or unlock new avenues for the application of biocatalysis.^1−3^ In recent years, promiscuous activity of different enzyme classes was utilized to widen the scope of biocatalytic methodologies for synthetic applications.^4,5^ However, enzymes only utilize a fraction of the different activation modes available to small molecule catalysts, which is the consequence of the limited number of functionalities present in the 20 canonical amino acids. This limitation can be overcome by creating designer enzymes using bioconjugation of protein scaffolds,^6^ supramolecular binding of unnatural cofactors,^7−9^ or genetic incorporation of noncanonical amino acids.^10−14^ The latter offers two main benefits; first, genetic incorporation techniques do not usually require posttranslational modification and thus no additional protein purification. Second, many different protein scaffolds can be chosen to incorporate the noncanonical amino acid and its position in the amino acid sequence can be varied as well, thus giving rapid access to a library of enzymes with different active site structures.^15−17^ Furthermore, the microenvironment around the catalytically active amino acid can be varied to suit the desired activation mode or to promote favorable interactions with the chosen substrate.^18,19^

**Figure 1:**
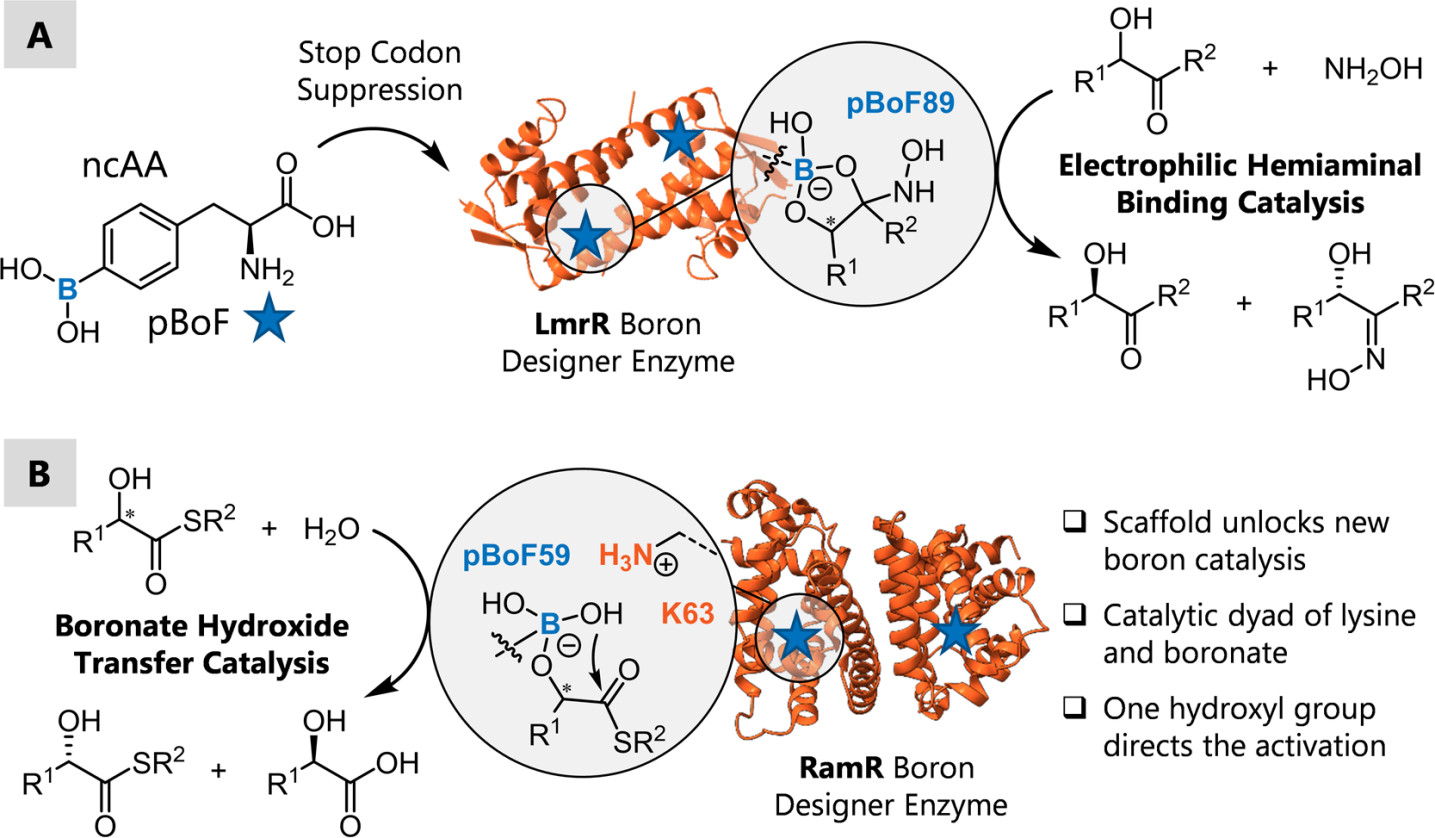
(A) Assembly of LmrR-based boron designer enzymes by genetic incorporation of para-boronophenylalanine (pBoF) and application as a catalyst for the oximation of α-hydroxyketones.^20^ (B). This work: Kinetic resolution of α-hydroxythioesters by boron-catalyzed hydroxide transfer enabled by interactions of an assisting lysine residue in the protein scaffold RamR.

Recently, we reported a boron designer enzyme created by incorporating the noncanonical amino acid para-boronophenylalanine (pBoF) into the multidrug resistance regulator LmrR (Figure 1A).^20^ This boron enzyme is capable of activating hemiaminal-type intermediates for the kinetic resolution of α-hydroxyketones by oximation. The mechanism of the reaction is unique to boron-based catalysts, which do not occur in nature.^21^ Subsequently, boron enzymes based on the LmrR scaffold have also been used by Xiang and co-workers for Friedel–Crafts alkylations using a phenol directing group to engage the boronic acid residue.^22^

Small-molecule catalysts based on boronic or borinic acids have been employed for many valuable organic transformations,^23−25^ but asymmetric catalysts are exceedingly rare.^26^ The general success of small molecule boron catalysts can be attributed to their versatile activation modes and activation of substrates proceeds via hydrogen bonding, Lewis acid interactions, or anhydride formation.^27^ Another example of boron catalysis is the ability to promote hydroxide transfer for the hydrolysis of hydroxyl group bearing imines, esters, and thioesters in aqueous solutions.^28−35^ In the reaction mechanism, the boron interacts with alcohols to form an anionic boronate intermediate, which can transfer a hydroxide to the nearby imine or (thio)ester, ultimately leading to hydrolysis. We envisioned that this type of boron catalysis could be used to perform a kinetic resolution reaction if the microenvironment of the protein preferentially promotes the reaction of only one enantiomer of the substrate. Here, we report that a designer boron enzyme based on the transcriptional regulator protein RamR enables the kinetic resolution of hydroxyl thioesters by a boron-catalyzed hydroxide transfer reaction assisted by a nearby lysine residue (Figure 1B).

## Results and Discussion

### Evaluating Different Boron Designer Enzymes

**Figure 2:**
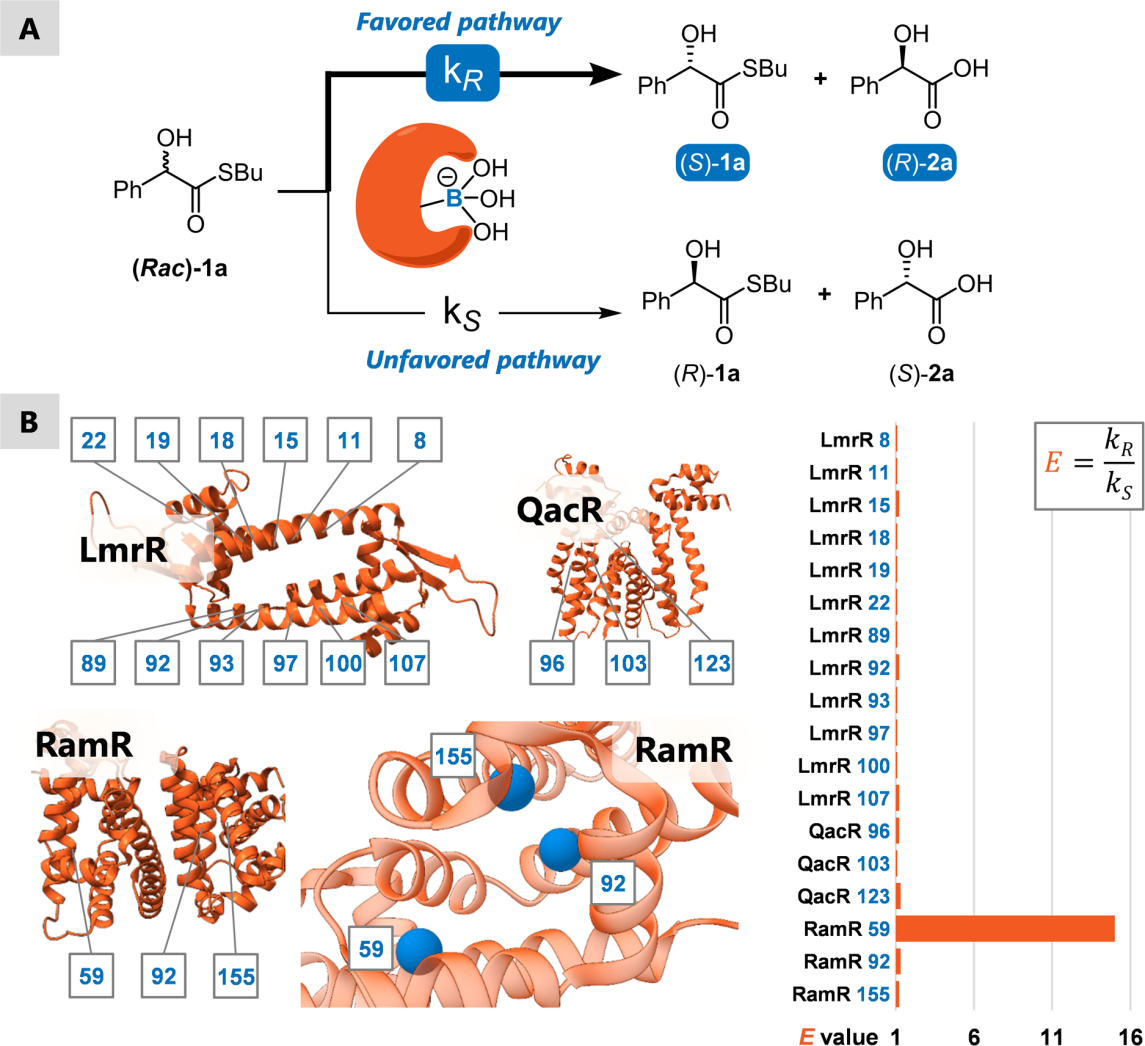
(A). Model reaction of the kinetic resolution by boron-catalyzed hydrolysis using thioester **1a**. (B). Results of screening of boron designer enzymes using the scaffolds LmrR, RamR, and QacR. Position of the pBoF residue in blue, *E* value obtained in the reaction in orange. Protein structure of the wild type protein shown (PDBs: LmrR (3F8B),^39^ RamR (3VVX),^40^ QacR (2HQ5)^38^), including a closer view of the hydrophobic pocket of RamR and the three positions chosen for pBoF incorporation. For reaction conditions, see SI.1.

We started our investigations by probing the hydrolysis of *S*-butyl 2-hydroxy-2-phenylethanethioate (**1a**) and found that the rate of reaction increased in a highly concentrated borate buffer compared to a phosphate buffer at the same pH, indicating that boron catalysis can indeed be used to accelerate the reaction (Figure 2A) (Supporting Information 2).^29^ Therefore, a panel of 18 boron designer enzymes was evaluated for the reaction, with a particular focus on selectivity. The enzymes were created by genetic incorporation of pBoF (Figure 2B), using the well-established stop codon suppression technology at various positions in three different protein scaffolds: LmrR, QacR, and RamR.^36−41^ These three dimeric proteins are transcriptional regulators without any innate catalytic function but contain one or two large promiscuous hydrophobic binding pockets, which make them ideal candidates to develop designer enzymes.^41−43^

The catalytic boron residues were placed into the proteins at positions inside or at the rim of their hydrophobic pockets. Interestingly, only one enzyme, RamR_Y59pBoF, showed any enantiodiscrimination in the model reaction (Figure 2B). RamR is originally a transcriptional regulator from *Salmonella typhimurium*, which itself has no inherent catalytic function.^40^ We optimized the reaction conditions and found that at pH 8.0, there is an optimum balance between enzyme activity and background hydrolysis, giving rise to an *E* value of 14 after 6 h (Supporting Information 2). The *E* value, also known as enantiomeric ratio, is a measure to quantify the enantiodiscrimination in a kinetic resolution reaction and describes the ratio of reaction rate of the preferred enantiomer to the less preferred enantiomer (Figure 2A).^44^ As the racemic background hydrolysis decreases the observed selectivity, we compared the activity of the preferred substrate (*R*)**-1a** and the less preferred (*S*)**-1a** using UV/vis spectroscopy (Supporting Information 3). This comparison allowed us to calculate the background corrected selectivity (E* value), which we determined to be 24. Free amino acid pBoF or RamR containing no boron residue had no activity under these reaction conditions (Supporting Information 2). Some lipases or esterases show promiscuous activity for thioesters^45,46^ and we evaluated two different commercially available lipases (Lipase Type VII and Amano Lipase PS); however, they were not active for the target transformation (Supporting Information 4).^47−49^ In contrast, porcine liver esterase has a higher activity when compared with the same catalyst loading used with the designer enzyme, but almost no enantioselectivity was observed, showcasing the difficulty to kinetically resolve thioesters.

### Investigating the Substrate Scope

**Scheme 1:**
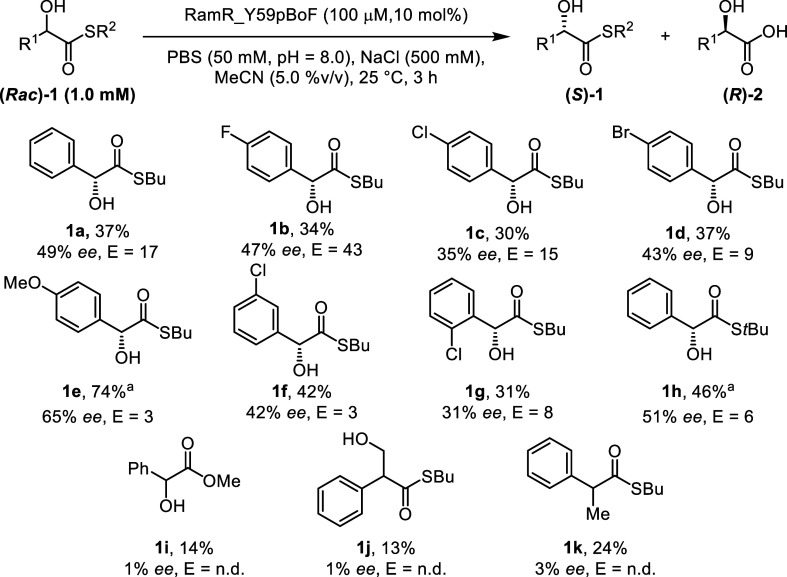
Overview of the Substrate Scope of the Kinetic Resolution of α-Hydroxythioesters Catalyzed by RamR_Y59pBoF. The Structure of the Preferentially Converted Enantiomer Is Given. Conversions and ee of the Remaining Starting Material Are Given. The Value Reported Is an Average of Two Experiments. n.d. = not Determined *t* = 16 h. For detailed reaction conditions, see Supporting Information 5.

With the optimized reaction conditions in hand, we investigated the substrate scope of the reaction (Scheme 1). The model substrate **1a** was converted, and an *E* value of 17 was reached. The reaction time can be prolonged (16 h) to recover starting material with enantiopurities of >99% (Supporting Information 5). Mandelic acid-derived butyl thioesters bearing para halogen substituents **1b**–**1d** gave similarly good results. Methoxy-substituted substrate **1e** as well as meta and ortho chloro-substituted substrates **1f** and **1g** were converted with overall much lower selectivity. The *tert*-butyl thioester **1h** reacted more sluggishly, and an *E* value of 6.3 was observed after a reaction time of 16 h. The boron enzyme did not show any catalytic activity on the corresponding *O*-methyl ester of mandelic acid **1i**. Substrate **1j**, which has more distance between the directing group and the reactive thioester, and substrate **1k**, which lacks a directing group, were both not suitable substrates for the boron enzyme. The fact that these substrates are not hydrolyzed supports the involvement of the boronic acid in activating the thioester using the hydroxyl group in the α-position as the directing group.

### Probing the Effect of the Protein Environment

**Figure 3:**
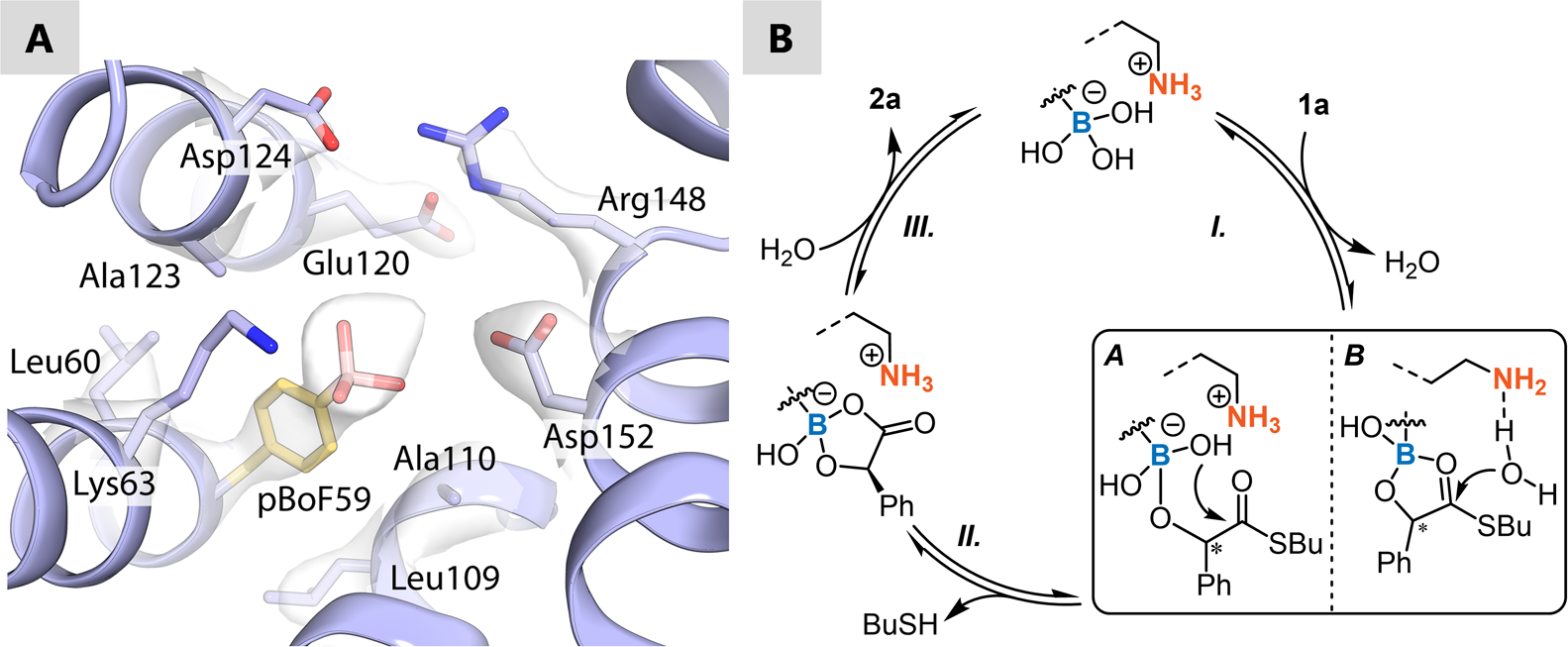
(A). Crystal structure of the hydrophobic pocket of RamR_Y59pBoF with pBoF59 residue and surrounding amino acids shown (PDB:9GTV) (Supporting Information 6). The gray surfaces represent the 2*F*_o_ – *F*_c_ electron densities (2.78 Å, contoured at 1σ) of the pBoF side chain and surrounding residues. (B). Proposed catalytic cycle of kinetic resolution of thioester **1a** by boron designer enzyme RamR_Y59pBoF.

As none of the other boron enzymes we tested showed any selectivity in the reaction, we set out to investigate why the position of the boron residue in the scaffold RamR is so important. We postulated that a residue in the proximity of the boronic acid in RamR_Y59pBoF has a large impact on the activity and selectivity. Therefore, we analyzed the crystal structure of RamR_Y59pBoF (2.78 Å resolution, crystallized at pH 8.5). The overall structure is very similar to RamR without a boronic acid residue (Supporting Information 6), but additional density at the para-position of the phenyl ring at position 59 clearly indicates the boronic acid residue, which seems to be mostly in the tetrahedral boronate form (Supporting Information 6). A closer look into the hydrophobic pocket reveals multiple amino acid side chains in the vicinity of the boron residue, including a lysine residue at position 63 (Figure 3A). We performed an alanine scan in the pocket and evaluated the catalytic activity of the respective mutants in the kinetic resolution of **1a** (Table 1).

**Table 1:**
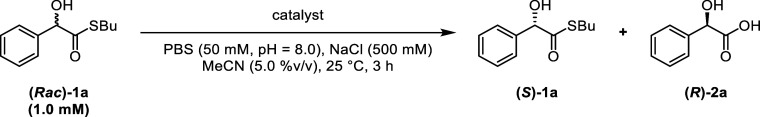
Catalytic Activity of Enzyme Variants in the Active Site of RamR_Y59pBoF^a^

entry	RamR_Y59pBoF variant	conversion 1a/%	*E* value
1	parent	25 ± 3	15
2	L60A	18 ± 2	4
3	K63A	1 ± 0	n.d.
4	K63R	0 ± 0	n.d.
5	K63M	0 ± 2	n.d.
6	K63N	0 ± 4	n.d.
7	L109A	12 ± 0	6
8	A110L	6 ± 0	14
9	E120A	20 ± 0	13
10	A123L	24 ± 3	8
11	D124A	14 ± 5	9
12	D152A	88 ± 1	3

a Reaction conditions: **1a** (1.0 mM, 200 nmol, 1.0 equiv), catalyst (50 μM, 5.0 mol %), PBS (50 mM, NaCl 500 mM, pH = 8.0), MeCN (5.0% v/v), 25 °C, 3 h. Conversion and selectivity determined with SFC using 2-phenylquinoline as the internal standard. The value reported is an average of two experiments. All error values are given as standard deviations. n.d. = not determined.

Most mutations around the pBoF residue negatively influence the activity (L60A, L109A, A100L, A123L, and D124A), whereas the mutation E120A has only a minor effect on the catalytic activity. The variant RamR_Y59pBoF_D152A showed a great improvement in catalytic activity, however with a loss of enantioselectivity under these reaction conditions (Table 1, entry 12). Notably, the K63A mutation gave an inactive enzyme with a complete loss of catalytic activity (entry 3). In the crystal structure, Lys63 lacks a clear electron density, indicating that its side chain is significantly disordered. However, the lowest energy side chain rotamer in a sterically favorable conformation available to Lys63 allows the formation of a direct interaction between the side chain ε-amino group and the boronic acid of pBoF (Nε(Lys63) to B(pBoF59) distance of ∼4.0 Å). The K63R mutation also proved detrimental, suggesting that a positive charge is not the only property relevant to catalysis (entry 4). However, it is also possible that steric hindrance prevents the more bulky arginine side chain from positioning its guanidinium group close to the boron. Mutants K63M and K63N also showed no catalytic activity (entries 5 and 6).

### A Hybrid Catalytic Dyad Containing a Boron-Based Amino Acid

Based on these observations, we believe that the canonical amino acid Lys63 and the noncanonical amino acid pBoF59 residue may form a hybrid catalytic dyad, in which the lysine acts similarly to serine/lysine dyads during the hydrolytic reaction catalyzed by certain serine peptidases.^50−58^ The term “hybrid catalytic dyad” refers to one canonical (Lys) and one noncanonical (pBoF) residue in designer enzymes that are in close proximity and are both essential for activity and selectivity in catalysis. We turned our attention to the reaction mechanism to gain insight into the role of both residues (Figure 3B). Based on the crystal structure, the boron enzyme is in an equilibrium between boronate and boronic acid under catalytic conditions, most likely shifted to the respective anionic boronate species (Supporting Information 6). In the ^11^B NMR spectrum, a chemical shift of 18.3 ppm for the boron residue was observed, mirroring the results of the crystal structure and indicating partial boronate formation under reaction conditions.^59^ The first step of the catalytic cycle is the formation of a boronate ester bond of the directing hydroxyl group with the boronic acid (Figure 3B(I)). We demonstrated the activity of RamR_Y59pBoF to form boronate ester bonds by reacting the boron enzyme with 4-nitrocatechol and following the reaction with HRMS or ^11^B NMR spectroscopy. We chose 4-nitrocatechol instead of substrate **1a** because the condensation of simple alcohols like **1a** does not form a stable cyclic boronate species, which can be detected by HRMS or ^11^B NMR spectroscopy. After incubation with 4-nitrocatechol, the observed mass of the enzyme shifted quantitatively to the boronate ester (Supporting Information 7). In the ^11^B NMR spectrum, a high field shift to 8.5 ppm upon the addition of 4-nitrocatechol is observed, which indicates the formation of a tetrahedral boronate ester (Supporting Information 8). Notably, the variant RamR_Y59pBoF_K63A shows identical ^11^B NMR shifts, with and without 4-nitrocatechol present, which suggests that this mutation does not substantially affect the catalytic boron center (Supporting Information 8). In the second step of the mechanism, the hydroxide of the boronate is transferred to the thioester group (Figure 3B(II)). Lys63 could facilitate this reaction step by providing a positive charge to stabilize the boronate intermediate or the oxyanion generated by the attack on the thioester (Figure 3B, intermediate A). Alternatively, Lys63 could act as a base catalyst according to previously reported assisted boron catalysis,^31^ and as it was observed for lysines in other catalytic dyads (Figure 3B, intermediate B) (Supporting Information 9).^50−58^ The lysine residue Lys63 is crucial for the enantioselectivity observed in the reaction, as other boron enzymes were giving only racemic conversion (Supporting Information 1). The resulting product of the hydrolysis is the product/enzyme complex, which we could observe via HRMS as a major adduct peak and also using ^11^B NMR spectroscopy with a high field shift of the boron signal to 6.8 ppm (Supporting Information 7 and 8). Interestingly, the structure of this complex includes a formal boronic acid/carboxylic acid anhydride. A mixed anhydride species is often an intermediate in boron-catalyzed reactions,^23^ and the observation of this species here could lead to new applications of boron designer enzymes, which we are currently investigating further.

### Site Saturation Mutagenesis of Residue Asp152

Based on the promising result of alanine mutant RamR_Y59pBoF_D152A (Table 1, entry 10), we performed site saturation mutagenesis at the Asp152 position to evaluate the effect of different amino acids. Overall, all amino acids aside from glutamic acid showed an increase in activity, however for many small or hydrophobic groups at the cost of selectivity (Supporting Information 10). The greatest improvements in quality of kinetic resolution were observed with the mutants D152Q (conversion 50%, *E* value = 24) and D152R (conversion 53%, *E* value = 17). The activity of these improved variants for the preferred substrate as determined using initial rates is in the range of 1.0 min^–1^, which is in the range of previously fully evolved boron designer enzymes in new-to-nature reactions.^20^ The results of the site saturation mutagenesis showcase the importance of the alkaline environment around the hybrid catalytic dyad. Likely, Asp152 has an unproductive interaction with Lys63, and removal of this interaction leads to a large increase in catalyst activity.

## Conclusions

We report a designer enzyme capable of asymmetric boron catalysis that operates like evolved natural enzymes: containing multiple key residues that work together in the active site, forming catalytic dyads or triads. Furthermore, we expanded the type of non-natural catalysis performed by boron designer enzymes to hydroxide transfer reactions. This new activation mode is enabled by a lysine residue in the protein scaffold RamR closely positioned next to the boron-containing amino acid pBoF. The emerging catalytic pBoF/lysine dyad proved essential for the kinetic resolution of α-hydroxy thioesters, and good selectivities were obtained with a variety of substrates. HRMS and ^11^B NMR spectroscopy revealed the reactivity of the boron enzymes in the key steps (substrate binding, hydroxide transfer, and product release) of the mechanism. The successful combination of non-natural reactive centers and the natural amino acids present in the protein scaffold enables more intricate enzyme designs, which can unlock new modes of activation and widen the scope of new-to-nature reactions in biocatalysis.

## Materials and Methods

### Representative Procedure for Protein Expression and Purification

A glycerol stock or single colony of *Escherichia coli* BL21-DE3 cells harboring the pET17b +_RamR mutant plasmid with a stop codon in the relevant position, as well as any other desired mutations, as well as the pEVOL_pBoF plasmid were used to inoculate 5.0 mL of Luria–Bertani medium containing ampicillin and chloramphenicol in a culture tube, which was then incubated overnight (37 °C, 135 rpm). The dense culture (1.0 mL) was then used to inoculate fresh Terrific Broth medium (100 mL) with the same antibiotics in a 500 mL Erlenmeyer flask. The culture was incubated (37 °C, 135 rpm) until OD_600_ ∼0.4 when pBoF and arabinose were added to final concentrations of 1 mM and 0.2%, respectively. The culture was further incubated (37 °C, 135 rpm) until OD_600_ ∼1.0 when protein expression was induced by adding IPTG (1.0 mM final concentration). The culture was incubated overnight (30 °C, 135 rpm), and the cells were harvested by centrifugation (4 °C, 6000 rpm, 20 min) and resuspended with Buffer X (50 mM NaH_2_PO_4_, 500 mM NaCl, pH = 8.0, 10 mL) and DENARASE (c-LEcta) was added (∼100 U final concentration). The cells were disrupted by sonication, and the cell debris was removed by centrifugation (4 °C, 12,000 rpm, 45 min). The supernatant was passed through a syringe filter (0.45 μm, Whatman) and applied to Strep-tag II resin (IBA Lifesciences) (4.0 mL). The resin was then washed with Buffer X (3 × 8 mL) and Buffer X containing a higher NaCl concentration (50 mM NaH_2_PO_4_, 1.0 M NaCl, pH = 8.0, 10 mL). The protein was eluted with Buffer X containing desthiobiotin (IBA Lifesciences, 5.0 mM, 12 mL). The protein was concentrated to 0.5 mL with a centrifugal filter and exchanged into the buffer used for catalysis (50 mM NaH_2_PO_4_, 500 mM NaCl, pH = 8.0) with a desalting column (GE Healthcare). The protein concentration was determined by absorbance at 280 nm measured with a Nanodrop 2000 (Thermo Scientific) with extinction coefficients estimated using the expasy Web server: https://web.expasy.org/protparam/.

### General Procedures for the Biocatalytic Kinetic Resolution Reaction

To a 2.0 mL Eppendorf plastic tube was added a designer enzyme in PBS buffer (50 mM, NaCl = 500 mM, pH = 8.0) with a total volume of 190 μL. Thioester **1** in MeCN (10 μL, 200 nmol, 20 mM stock, 1.0 mM final concentration) was added. The reaction mixture was gently shaken (450 rpm) at 25 °C for 16 h. 2-Phenyl quinoline in MeCN (10 μL, 40 nmol, 4 mM stock) was added, and the reaction mixture was extracted with *n*BuOH (500 μL). After strong mixing and short centrifugation, the organic layer (400 μL) was separated and washed with H_2_O (300 μL). The organic layer (200 μL) was then dried using anhydrous Na_2_SO_4_ and directly analyzed using HPLC (injection volume of 20 μL) or SFC (injection volume of 5.0 μL).

## Supporting Information Available

Crystallographic data for the structures of RamR_Y59pBoF has been deposited in the Protein Data Bank with accession number 9GTV. Screening of microenvironments in multidrug-resistant regulator-based protein scaffolds; reaction optimization for kinetic resolution of **1a**; determination and validity of *E* values; investigation of natural enzymes and substrate scope for kinetic resolution of thioesters; structural details of RamR_Y59pBoF and HRMS adduct study of RamR_Y59pBoF with **2a**; ^11^B NMR of RamR_Y59pBoF in the presence of different ligands; role of Lys63 in the catalytic cycle; site saturation mutagenesis of Asp152; general considerations; additional data on X-ray crystallography; molecular biology procedures; synthetic chemistry information; HPLC and SFC chromatograms; NMR spectra; MS spectra of purified proteins; and additional references (PDF)
