# Supplementary material for: Boron Designer Enzyme with a Hybrid Catalytic Dyad
Source: ACS Catal. 2024 Dec 3;14(24):18469–76. doi: 10.1021/acscatal.4c06052 (PMC11667675; doi:10.1021/acscatal.4c06052)
Supplement: Supplementary file 1 — cs4c06052_si_001.pdf [file cs4c06052_si_001.pdf]

Supporting Information

Boron Designer Enzyme with a Hybrid Catalytic Dyad

Lars Longwitz[1], Marijn D. Kamer[1], Bart Brouwer[1], Andy-Mark W. H. Thunnissen[2], Gerard Roelfes[1]*

[1]Stratingh Institute for Chemistry, University of Groningen, 9747, AG, Groningen, The Netherlands

[2]Groningen Biomolecular Sciences and Biotechnology Institute, University of Groningen, 9747, AG, Groningen, The Netherlands

SI.1	Screening of microenvironments in multidrug resistant regulator based protein scaffolds	3
SI.2	Reaction optimization for the kinetic resolution of 1a	5
SI.3	Determination and Validity of the E value and determination of corrected E values	6
SI.4	Investigation of natural enzymes for the kinetic resolution of thioesters	9
SI.5	Substrate scope of the kinetic resolution of thioesters	10
SI.6	Structural details of RamR_Y59pBoF obtained from X-ray crystallography	11
SI.7	HRMS adduct study of RamR_Y59pBoF with mandelic acid (2a)	13
SI.8	11B NMR of RamR_Y59pBoF in the presence of different ligands	14
SI.9	The role of Lysine 63 in the catalytic cycle	16
SI.10	Site saturation mutagenesis of D152	17

Procedures and Supplementary Data

SI.11	General considerations	20
SI.12	Additional information on the X-ray crystallography	22
SI.13	Molecular biology procedures (Protein expression, mutagenesis, protein analytics)	24
SI.14	Synthetic chemistry (General procedures, substrate synthesis, reaction analysis)	34
SI.15	HPLC and SFC chromatograms	41
SI.16	NMR spectra	53
SI.17	MS spectra of purified proteins	71
SI.18	References of supporting information	85

SI.1 Screening of microenvironments in multidrug resistant regulator based protein scaffolds

Table S1. Evaluation of different microenvironments for boron catalyzed thioester hydrolysis.[a]

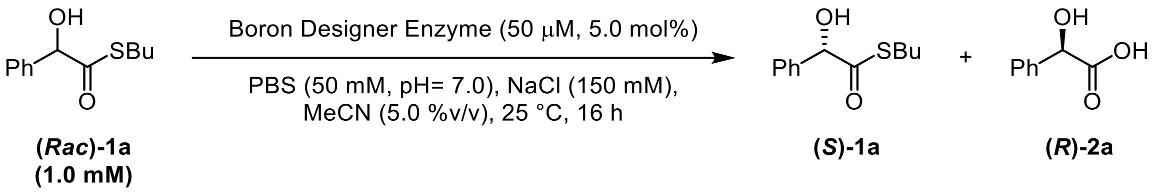

Entry	Boron Designer Enzyme	Conversion **1a**/ %	E value
1	LmrR_M8pBoF	33 ± 4	1
2	LmrR_A11pBoF	5 ± 1	1
3	LmrR_V15pBoF	6 ± 1	1
4	LmrR_L18pBoF	9 ± 2	1
5	LmrR_N19pBoF	7 ± 3	1
6	LmrR_K22pBoF	11 ± 5	1
7	LmrR_M89pBoF	3 ± 1	1
8	LmrR_A92pBoF	5 ± 1	1
9	LmrR_F93pBoF	9 ± 0	1
10	LmrR_S97pBoF	9 ± 2	1
11	LmrR_D100pBoF	6 ± 0	1
12	LmrR_E107pBoF	8 ± 1	1
13	QacR_Q96pBoF	11 ± 2	1
14	QacR_Y103pBoF	7 ± 0	1
15	QacR_Y123pBoF	9 ± 1	1
16	RamR_Y59pBoF	5 ± 0	15
17	RamR_Y92pBoF	3 ± 0	1
18	RamR_F155pBoF	7 ± 2	1

[a] Reaction conditions: **1a** (1.0 mM, 200 nmol, 1.0 equiv), catalyst (5.0 mol%, concentration of dimer), PBS (50 mM, NaCl 150 mM, pH= 7.0), MeCN (5.0 %v/v), 25 °C, 16 h. Conversion and selectivity determined with SFC using 2-phenyl quinoline as the internal standard. If not otherwise specified, the value reported is an average of two experiments. All error values are given as standard deviations.

From all boron designer enzymes evaluated, only RamR_Y59pBoF showed enantioselectivity in the kinetic resolution reaction of thioesters (Table S1, entry 16). Under the given reaction

conditions, we observed precipitation of the enzyme RamR_Y59pBoF, which could be decreased by increasing the pH or increasing salt concentration. For this reason, further experiments were conducted with higher NaCl concentration (500 mM).

SI.2 Reaction optimization for the kinetic resolution of 1a

Table S2. List of conducted experiments with natural enzymes.[a]

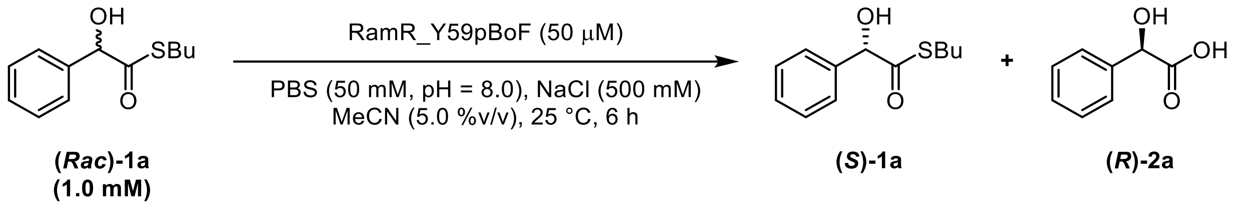

Entry	Deviations from standard conditions	pH	Conversion 1**a**/ %	E value
1	no catalyst	7.0	1 ± 0	-
2	$B(OH)_3$ (20 mM)	7.0	38 ± 1	-
3		6.0	1 ± 2	-
4		6.5	6 ± 2	3
5		7.0	9 ± 0	4
6		7.5	27 ± 3	8
7		8.0	41 ± 2	14
8	No catalyst, 22 h	8.0	14 ± 0	-
9		8.5	51 ± 1	14
10		9.0	49 ± 2	11
11	No catalyst, 22 h	9.0	16 ± 1	-
12	Tris (20 mM)	8.0	19 ± 3	4
13	HEPES (20 mM)	8.0	39 ± 1	7
14	T = 4 °C, t = 24 h	8.0	16 ± 1	7
15	T = 37 °C, t = 1 h	8.0	68 ± 1	2
16	$B(OH)_3$ (50 μM)	8.0	2 ± 1	-
17	pBoF (50 μM)	8.0	6 ± 0	-
18	RamR (wild type) (50 μM)	8.0	2 ± 0	-
19	Biological duplicate	8.0	37 ± 2	13

[a] Reaction conditions: **1a** (1.0 mM, 200 nmol, 1.0 equiv), RamR_Y59pBoF (50 μM), PBS (50 mM, NaCl 500 mM, pH= 7.0 or 8.0), MeCN (5.0 %v/v), 25-50 °C, 15 min-6 h. Conversion and selectivity determined with SFC using 2-phenyl quinoline as the internal standard. If not otherwise specified, the value reported is an average of two experiments. All error values are given as standard deviations.

SI.3 Determination and Validity of the E value and determination of corrected E values

E values were determined using either substrate *ee* and calculated conversion based on internal standard.

$$E = \frac{\ln[(1-c)(1-ee_S)]}{\ln[(1-c)(1+ee_S)]}$$

This formula gives the uncorrected E value as it is observed in the experiment. However, when changing catalyst loading or reaction time, the E-value changes during the reaction due to the presence of a relatively fast uncatalysed reaction.

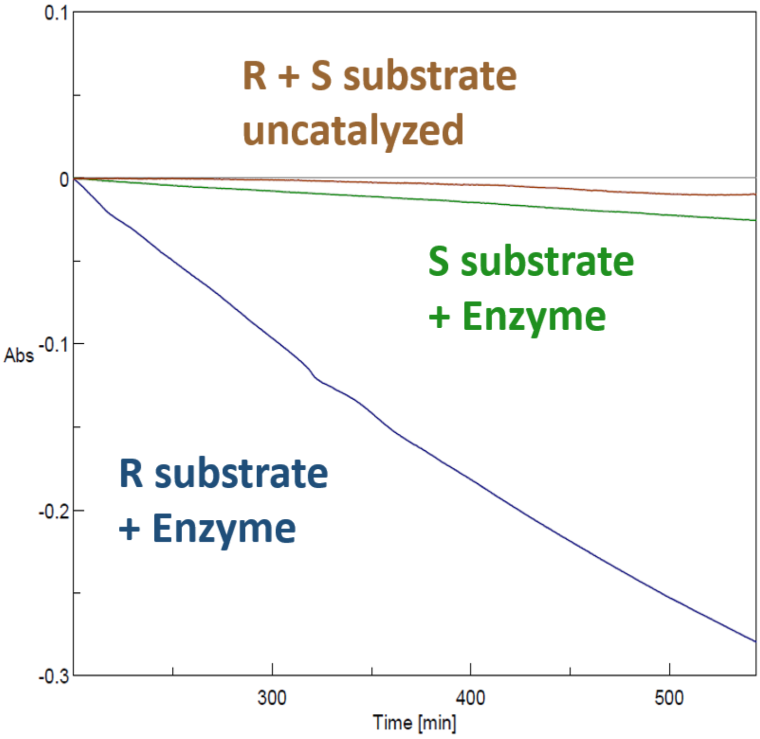

Figure S1. Absorbance time plots monitored at 237 nm obtained when converting (*Rac*)-**1a** without a catalyst and (*R*) or (*S*) substrate **1a** in the presence of catalyst.

An uncatalyzed background towards the same product as the enzymatic reaction will lead to an underestimation of the real E value, if standard equations are used.[1] For this reason, we evaluated the rates of the individual enantiomers using UV/Vis spectroscopy to follow the reaction. A typical time course is given in Figure S1. In this case, the following equation can be used for the determination of the E value.

$$E = \frac{k(R)}{k(S)} = \frac{k\mathrm{cat(R)}/K\mathrm{m}(R)}{k\mathrm{cat}(S)/K\mathrm{m}(S)}$$

As we were unable to obtain full Michaelis-Menten kinetics due to the low solubility of the substrate, the overlap of enzyme absorbance with the substrate (237 nm) and relatively slow kinetics, we used the initial rates to determine the E value. The comparison of initial rates to determine the E value is valid for scenarios where the substrate concentration is much lower than K_m or the K_m of both enantiomers is identical, which seems to be the case for RamR_Y59pBoF.[1]

While the background reaction without catalyst and the less preferred (*S*)-**1a** were measured over hours, initial rates from (R)-1a were extracted between 0 and 30 min of reaction time. The absorbance was converted using the absorption coefficient of the substrate (237 M^{-1} mm^{-1}) and the product (40 M^{-1} mm^{-1}). The activity observed in the uncatalysed reaction of racemic substrate of reaction run was then removed to obtain the catalyzed reaction rate. Comparing the catalyzed rate observed of the (*S*) substrate and the (*R*) substrate gives the corrected E value (E*). This value is a closer representation of the true selectivity of the enzyme.

Table S3. Kinetic analysis of activity and selectivity of RamR_Y59pBoF.[a]

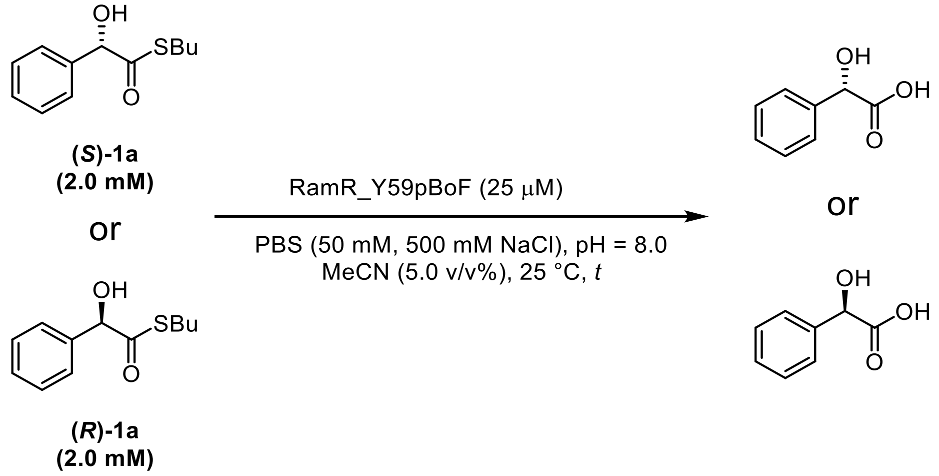

Entry	Substrate	Catalyst	Activity μM/h	TOF /h	E	E*
1	Rac	-	9.7 ± 2.2	-	-	-
2	S	Parent	30.2 ± 0.9	0.8 ± 0.1	17	24
3	R	Parent	509 ± 13	20 ± 1		

[a] Reaction conditions: **1a** (2.0 mM, 400 nmol, 1.0 equiv), catalyst (25 μM), PBS (50 mM, NaCl 500 mM, pH= 8.0), MeCN (5.0 %v/v), 25 °C. Conversion was monitored via UV/Vis spectroscopy at 237 nm. If not otherwise specified, the value reported is an average of two experiments. All error values are given as standard deviations.

For RamR_Y59pBoF, an E value of 17 and a corrected E* value of 24 are obtained. This fits well with the E values observed using SFC measurements and showcases a significant influence

of the background on the outcome of the reaction. While we could use the initial rates to obtain results that were in good correlation with our observed E values, we observed that for improved mutants, this was not the case anymore. Therefore, the E value that is observed using SFC analysis of the converted racemic substrate gives a more accurate depiction of the E value for improved variants. In these cases with higher enzyme activity, the background reactivity of the buffer also becomes negligible, and it is no longer necessary to take into account.

SI.4 Investigation of natural enzymes for the kinetic resolution of thioesters

Table S4. List of conducted experiments with natural enzymes.[a]

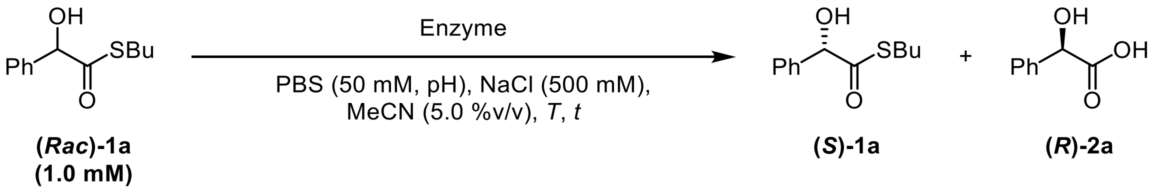

Entry	Catalyst	U	*T* / °C	*t*	pH	Conv. **1a**/ %	E
1	Lipase Type VII	1100	25	6 h	7.0	0 ± 2	1.0
2	Lipase Type VII	0.2	37	15 min	8.0	2 ± 1	1.0
3	Amano Lipase PS	20	25	6 h	7.0	3 ± 3	1.0
4	Amano Lipase PS	0.2	50	15 min	8.0	10 ± 0	1.0
5	Esterase from porcine liver	400	25	15 min	8.0	100 ± 0	-
6	Esterase from porcine liver	4	25	15 min	8.0	100 ± 0	-
7	Esterase from porcine liver	2	25	3 h	8.0	98 ± 2	-
8	Esterase from porcine liver	1	25	3 h	8.0	34 ± 6	2.3

[a] Reaction conditions: **1a** (1.0 mM, 200 nmol, 1.0 equiv), catalyst (concentration given as U in the table), PBS (50 mM, NaCl 500 mM, pH= 7.0 or 8.0), MeCN (5.0 %v/v), 25-50 °C, 15 min-6 h. Conversion and selectivity determined with SFC using 2-phenyl quinoline as the internal standard. If not otherwise specified, the value reported is an average of two experiments. All error values are given as standard deviations.

Different commercially available lipases and an esterase were evaluated for the model reaction. We performed reactions both with high enzyme loading similar to the designer enzymes and under reaction conditions as proposed by the manufacturer (lower loadings, different pH and temperature). Lipase Type VII from *Candida Rugosa* and Amano Lipase PS from *Burkholderia Cepacia* did not show any activity for the hydrolysis of thioester **1a** (Table S4, entries 1-4). Esterase from porcine liver showed high activity for the substrate, quickly reaching full conversion (entries 5, 6 and 7). After adjusting the catalyst loading, we found that while this esterase shows high activity, only marginal enantioselectivity and an E value of 2.3 were observed (entry 8). Interestingly, porcine liver esterase preferably converts the (*S*) substrate **1a** in contrast with RamR_Y59pBoF. The result with these robust enzymes show that kinetic resolution of thioesters is a challenging reaction, which is not easily solved by using natural enzymes.

SI.5 Substrate scope of the kinetic resolution of thioesters

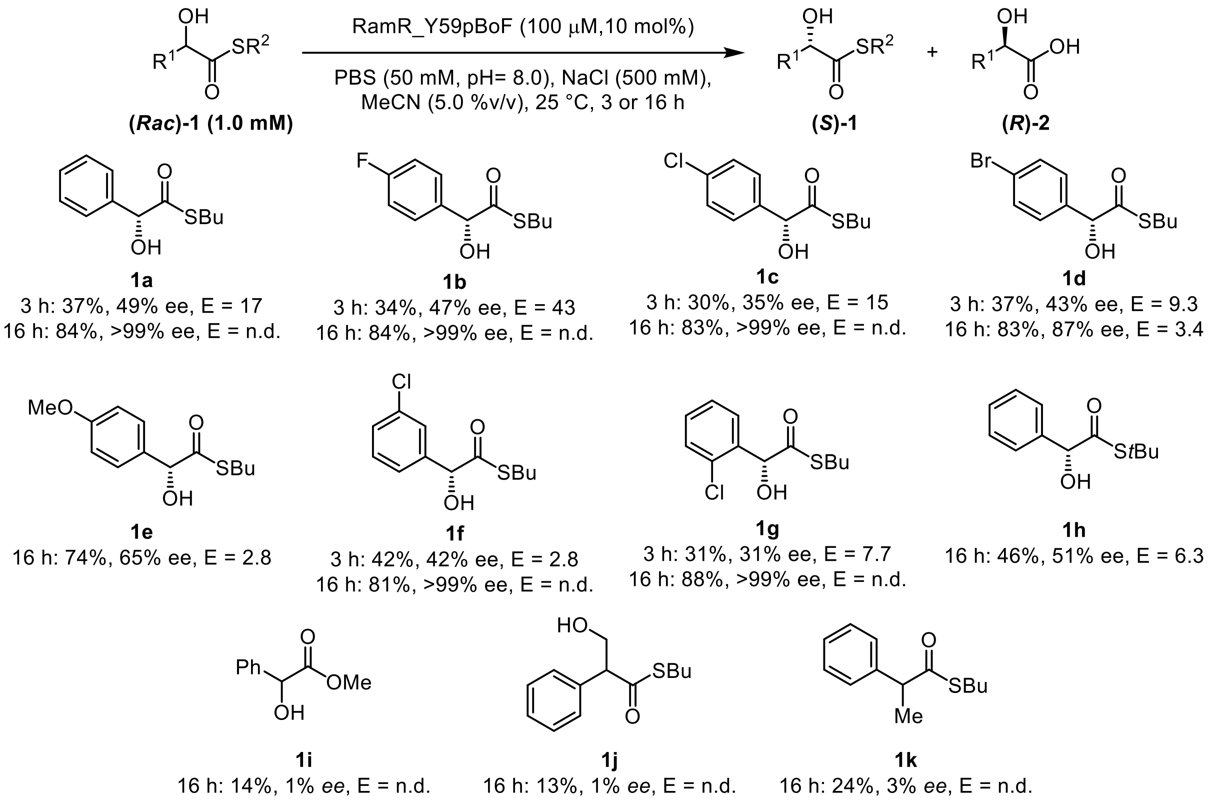

Scheme S1. Overview of the substrate scope of the kinetic resolution of α-hydroxythioesters catalyzed by RamR_Y59pBoF. The structure of the preferentially converted enantiomer is given. Conversions and *ee* of the remaining starting material is given. If not otherwise specified, the value reported is an average of two experiments. Reaction time was varied to obtain results between 30-70% conversions for accurate E value determination. (n.d. = not determined). Reaction conditions: **1** (1.0 mM, 200 nmol, 1.0 equiv), catalyst (100 µM, 10 mol%), PBS (50 mM, NaCl 500 mM, pH= 8.0), MeCN (5.0 %v/v), 25 °C. Conversion and selectivity determined with SFC or HPLC using 2-phenyl quinoline as the internal standard. The value reported is an average of two experiments. All error values are given as standard deviations.

SI.6 Structural details of RamR_Y59pBoF obtained from X-ray crystallography

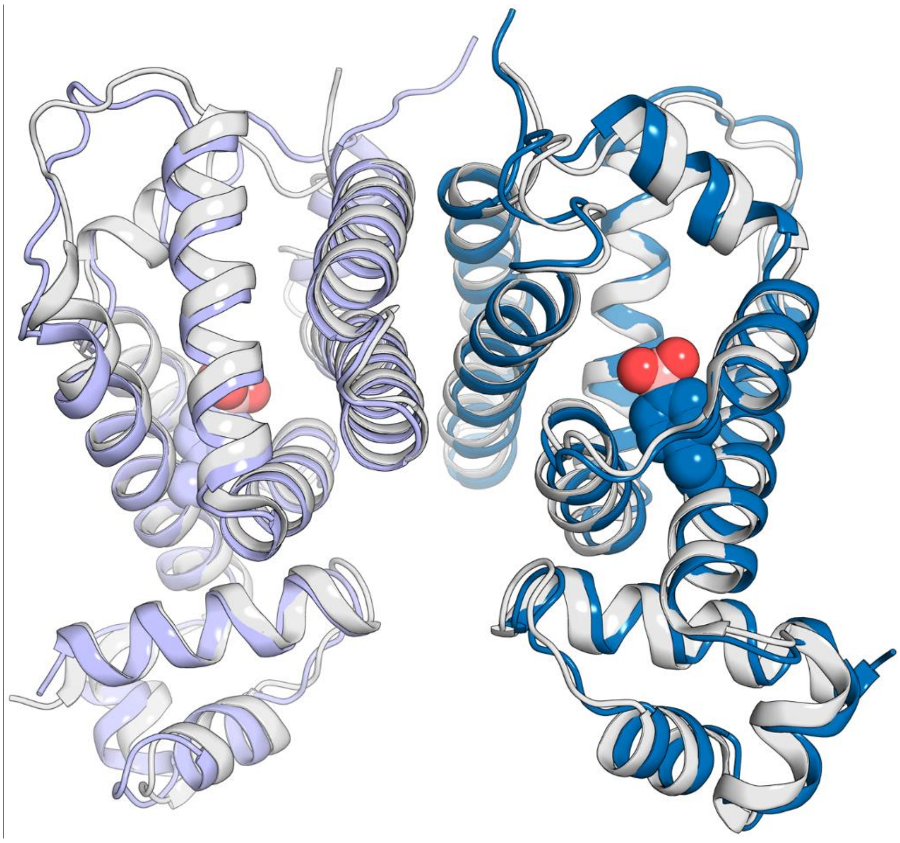

Figure S2. Crystal structure of the RamR_Y59pBoF dimer (light and dark blue, PDB: 9GTV, chains A and B) superimposed to wild type RamR (light grey, PDB: 3VVX). The pBoF residues are depicted as spheres.

The Cα-backbone RMSDs of RamR-Y59pBoF versus wild-type RamR subunits range between 0.46-0.70 Å (Figure S2), while the mutual Cα-backbone RMSDs of the four polypeptide chains in the asymmetric unit of RamR-Y59pBoF range between 0.19 and 0.50 Å. Thus, incorporation of the *pBoF* residue does not significantly affect the overall conformation of RamR. Regarding the boronic acid residue, the boron can either be in the free boronic acid form, which is trigonal planar, or present as the anionic boronate. This acid boronate equilibrium is present in solution and since the pkA of boronic acids is around the pH of the reaction buffer (8.0), both forms are expected to co-exist. The electron density observed in the crystal structure, which was obtained at pH 8.5, points more towards a boronate structure, as the electron density is not in plane with the aryl ring and seems to fit a tetragonal boron (Figure S3). The 11B NMR spectrum of the enzyme also points to a mixture of boronic acid and boronate, as the observed shift of 18.3 ppm is neither a free boronic acid (~30 ppm) nor a boronate species (~5 ppm).

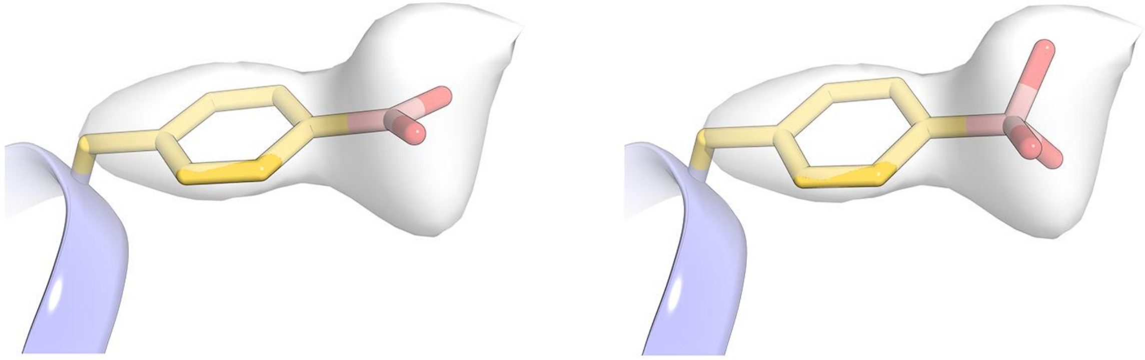

Figure S3. F_o-F_c omit difference map at 2.78 Å resolution (grey, contoured at 3σ) of the pBoF residue (chain C). The fitted *pBoF* residue is shown either in the free acid state (panel left) or in its boronate state (panel right).

SI.7 HRMS adduct study of RamR_Y59pBoF with mandelic acid (2a)

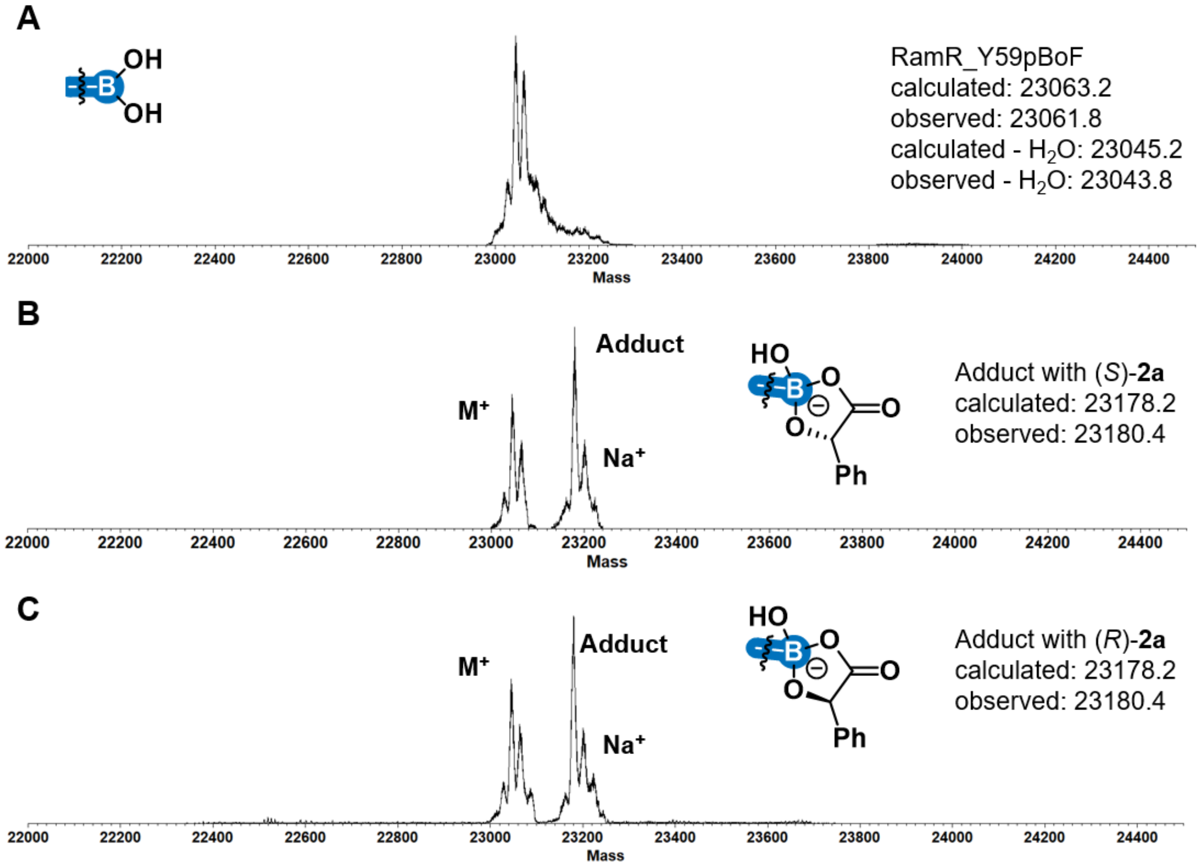

Figure S4. Direct injection HRMS spectra of RamR_Y59pBoF (A) and incubated with *(S)*-**2a** (B) or *(R)*-**2a** (C) showing the formation of an equal amount of boronate ester adducts independent of enantiomer.

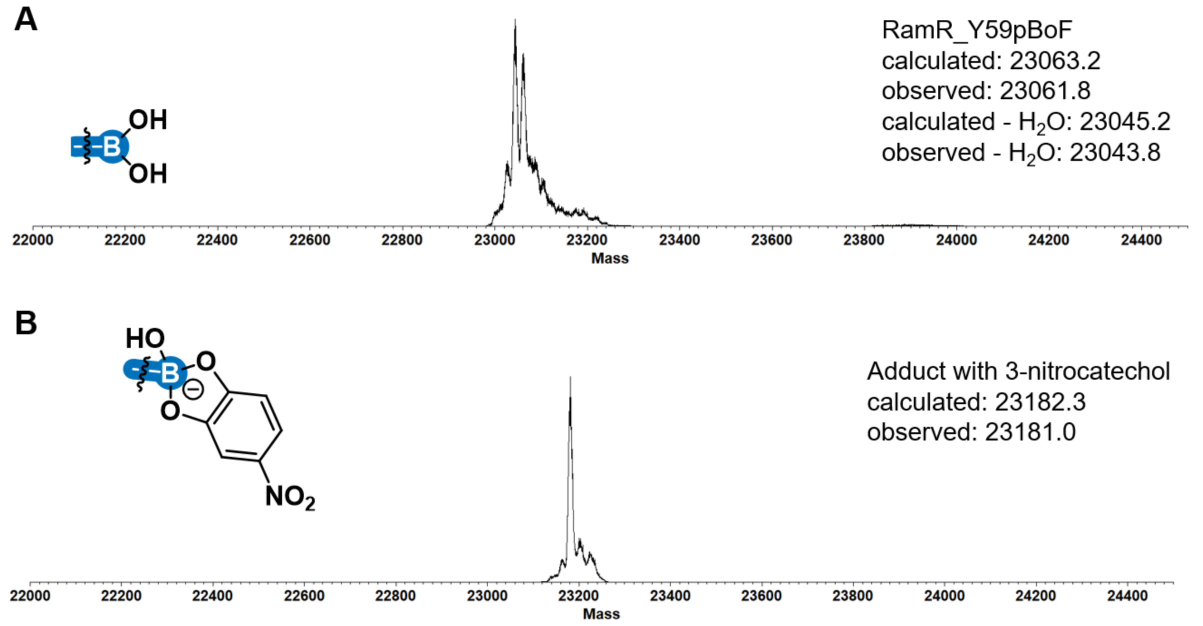

Figure S5. Direct injection HRMS spectra of RamR_Y59pBoF (A) and incubated with 4-nitrocatechol (B).

SI.8 ^{11}B NMR of RamR_Y59pBoF in the presence of different ligands

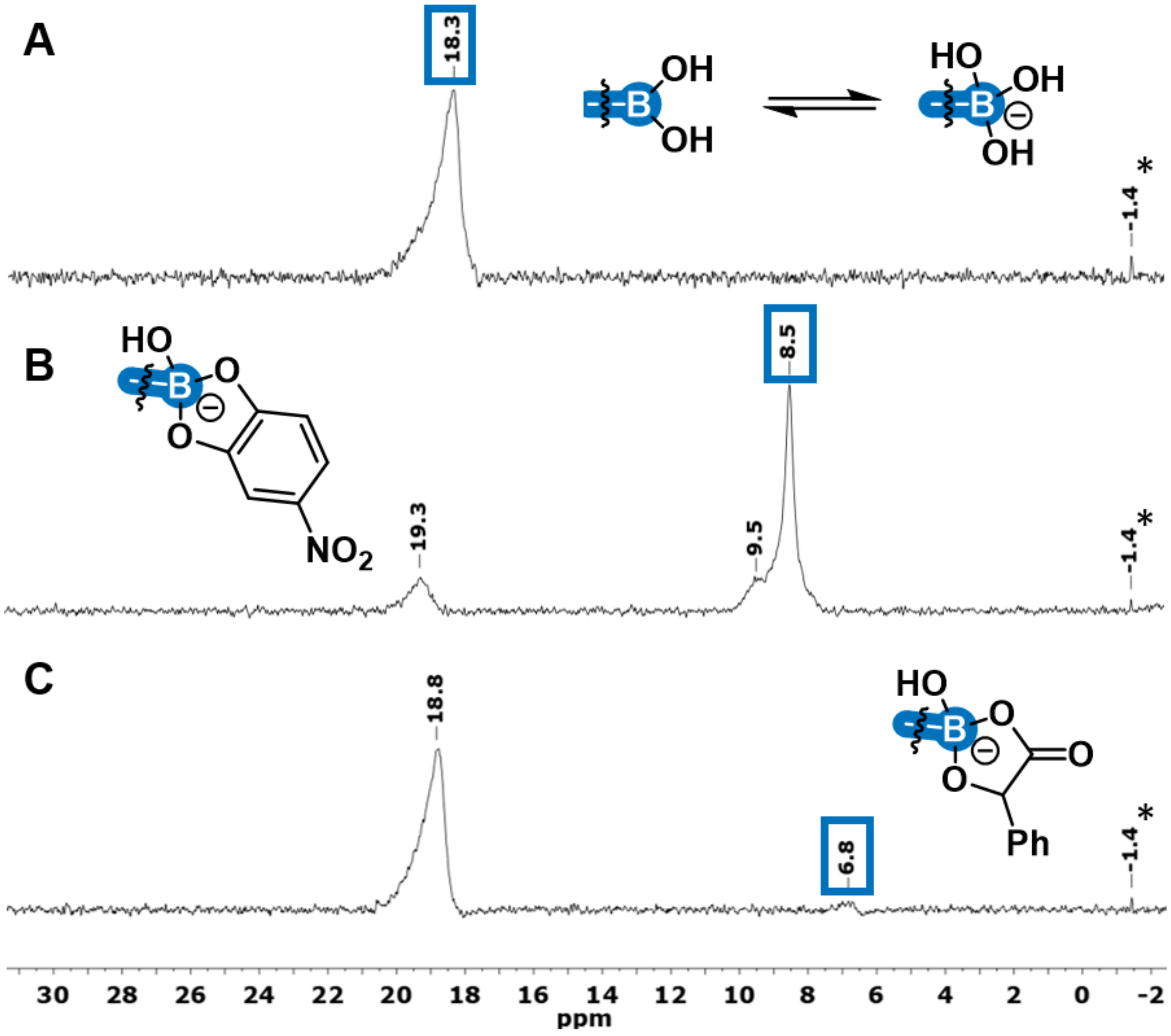

Figure S6. ^{11}B NMR spectra of RamR_Y59pBoF in PBS buffer at pH 8.0 in the presence of no additional ligands (A), 4-nitrocatechol (B) and Mandelic acid (*Rac*)-**2a** (C). The internal standard $NaBF_4$ has a chemical shift of -1.4 ppm (*).

We conducted ^{11}B NMR experiments of RamR_Y59pBoF, which showed high field shifting (δ = 18.3 ppm) compared to the free amino acid pBoF (δ = 25.4 ppm) at the same pH.[2] The shift indicates the acid base equilibrium between free acid and boronate species is influenced by the microenvironment of the protein and more of the negatively charged boronate species is present und catalysis conditions (pH 8.0). However, this high field shifting of the boron NMR signal is also present in other inactive mutants though (e.g., LmrR_V15pBoF)[2] and also the RamR_Y59pBoF_K63A mutant shows an identical spectrum (δ = 18.3 ppm). The K63A variant also does not show differences when comparing with the parent in the presence of 4-

nitrocatechol (Figure S6B vs S7C). Therefore, it cannot be concluded that the shift is in direct correlation with the residues of the protein environment, as it is most likely closer related to the electronics on the boron atom. As there is no difference in the spectrum of parent and K63A, it is also more likely that Lys63 has a more active role in the catalytic cycle and is not fine tuning the electronic parameters of the boron moiety to improve substrate binding or activation.

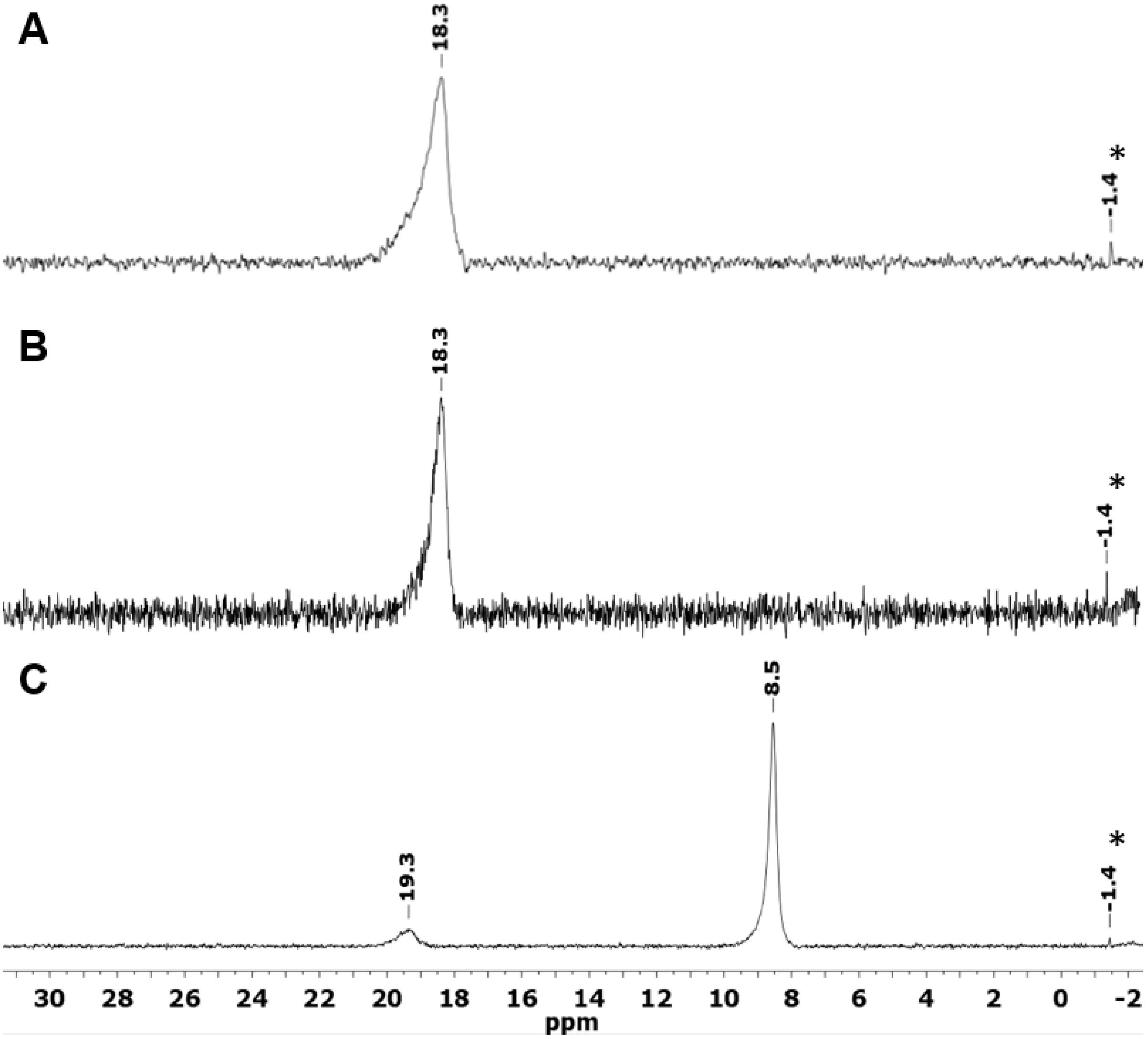

Figure S7. ^{11}B NMR spectra of RamR_Y59pBoF in PBS buffer at pH 8.0 (A), RamR_Y59pBoF_K63A under the same conditions (B) and RamR_Y59pBoF_K63A in the presence of 4-nitrocatechol (C).

SI.9 The role of Lysine 63 in the catalytic cycle

The lysine could facilitate the hydrolysis of the thioester substrate in different ways. A purely structural importance of the residue is unlikely, as alanine K63A, arginine K63R methionine K63M and asparagine K63N mutants were well expressed and K63A showed an almost identical 11B NMR spectrum to the parent. We also tried expressing a variant bearing a K63D mutation, which did not yield any enzyme. Furthermore, interactions based on charge stabilization are also unlikely, as the K63R mutation is completely deleterious. We propose two possible ways for the lysine residue to enable the enantioselective hydrolysis of thioester in combination with the boron residue (Figure S8). The lysine could provide stabilizing interactions to transfer a hydroxide from the boron to the substrate in an enantioselective manner. Alternatively, the lysine could also act as a general base catalyst to deprotonate a water molecule which then attacks the substrate, which is activated by boron. We did not find any evidence for the binding of the substrate to the Lewis acidic boron center as depicted on the right intermediate, so we believe the hydroxide transfer pathway is more likely.

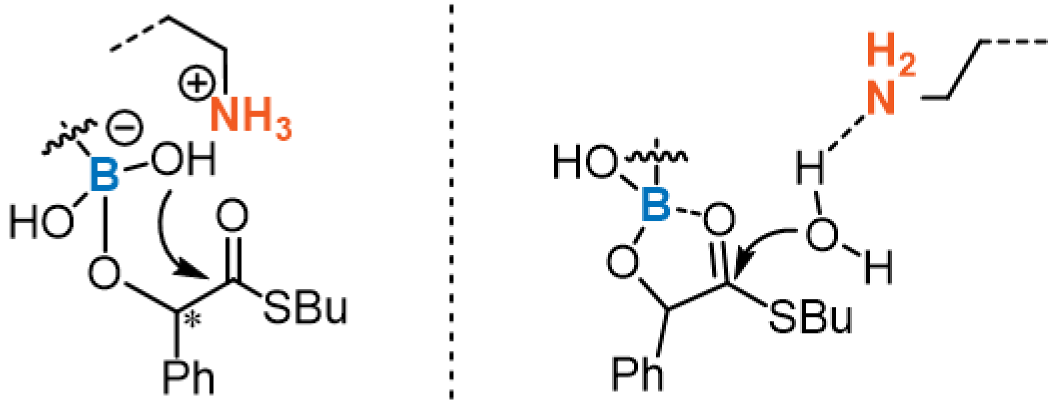

Figure S8. Proposed role of Lysine 63 in the boron catalyzed hydrolysis of thioesters. Left: Assisted hydroxide transfer. Right: Deprotonation of the attacking water molecule.

SI.10 Site saturation mutagenesis of Asp152

Table S5. Catalytic activity of enzyme variants in the active site of RamR_Y59pBoF.[a]

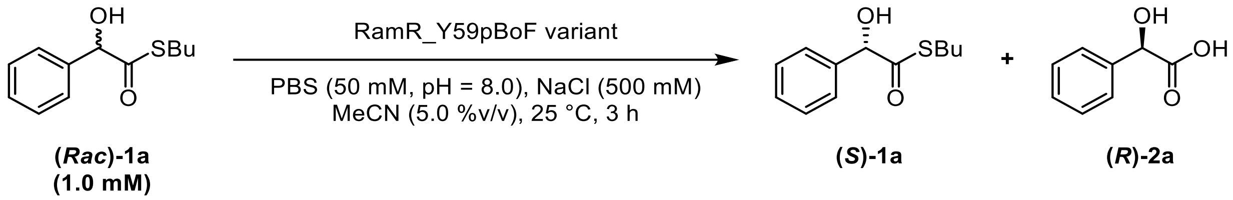

Entry	RamR_Y59pBoF variant	Conversion **1a**/ %	E value
1	Parent	25 ± 3[b]	15[b]
2	D152A	88 ± 1[b]	3[b]
3	D152C	80 ± 0	3
4	D152E	27 ± 3	11
5	D152F	97 ± 1	2
6	D152G	95 ± 1	2
7	D152H	63 ± 4	10
8	D152I	82 ± 2	4
9	D152K	60 ± 1	7
10	D152L	69 ± 3	7
11	D152M	76 ± 2	5
12	D152N	61 ± 2	18
13	D152P	66 ± 5	8
14	D152Q	50 ± 1[b]	24[b]
15	D152R	53 ± 2 [b]	17[b]
16	D152S	68 ± 4	7
17	D152T	65 ± 2	6
18	D152V	74 ± 0	7
19	D152W	71 ± 8	5
20	D152Y	59 ± 1	17

[a] Reaction conditions: **1a** (1.0 mM, 200 nmol, 1.0 equiv), catalyst (50 µM, 5.0 mol%), PBS (50 mM, NaCl 500 mM, pH= 8.0), MeCN (5.0 %v/v), 25 °C, 3 h. Conversion and selectivity determined with SFC using 2-phenyl quinoline as the internal standard. If not otherwise specified, the value reported is an average of two experiments. All error values are given as standard deviations. [b] Average values of biological duplicates. Reactions performed in duplicate.

Based on the results of the alanine scan conducted (Table 1), we decided to perform site saturation mutagenesis on Asp152. The effects of different amino acids at this position for the model thioester hydrolysis reaction was investigated using purified enzymes (Table S5). In general, all amino acid side chains aside from glutamic acid (entry 4) showed an increase in catalytic activity. An acidic environment is thus hindering the thioester hydrolysis by the 59pBoF and Lys63 residues, indicating the importance of an alkaline environment. The introduction of small or hydrophobic groups (entries 2, 5, 6, 8, 10, 13, 18 and 19) yields a decrease in enantioselectivity. The selectivity does increase when introducing asparagine, glutamine or arginine (entries 12, 14 and 15, respectively), with the best catalysts (D152Q and D152R) yielding conversions of close to 50% and E values of 24 and 17, respectively. Merely including a basic amino acid at this position does not directly lead to an increase in selectivity, as indicated by the results of basic residues like histidine and lysine (entries 7 and 9). This indicates a more complex method of interaction with the substrate in the active site of the artificial enzyme.

The most promising mutants as assessed by SFC were also subjected to analysis using UV/Vis spectroscopy to obtain initial rates of the different mutants with both enantiomers of the starting material (Table S6). Also the mutant D152A that proved less selective than RamR_Y59pBoF was assessed. The catalyst loading of the evolved mutants was adapted from the conditions in which the parent was tested, as the hydrolysis in the mutants proceeded too fast to conduct accurate UV/Vis measurements in our experimental setup. The data obtained shows that mutations at this position significantly increase the activity on the preferred substrate **(*R*)- 1a** (2.1-fold and 3.2-fold for D152Q and D152R compared to parent, Table S6 entries 5-8). Notably, the enzyme RamR_Y59pBoF_D152A shows a 31-fold increase in activity on the preferred substrate **(*R*)- 1a**. However, as observed in the kinetic resolution experiments, the activity on the non-preferred substrate **(*S*)- 1a** is also increased (51-fold), while this increase is not as severe for the more selective variants D152Q and D152R (entries 5 and 7). Generally, the results match well to the data obtained by kinetic resolution of the racemate (Table S5) with RamR_Y59pBoF_D152Q and RamR_Y59pBoF_D152R showing the highest improvements in combined selectivity and activity.

Table S6. Kinetic analysis of activity and selectivity of RamR_Y59pBoF and the improved alanine mutant RamR_Y59pBoF_D152A.[a]

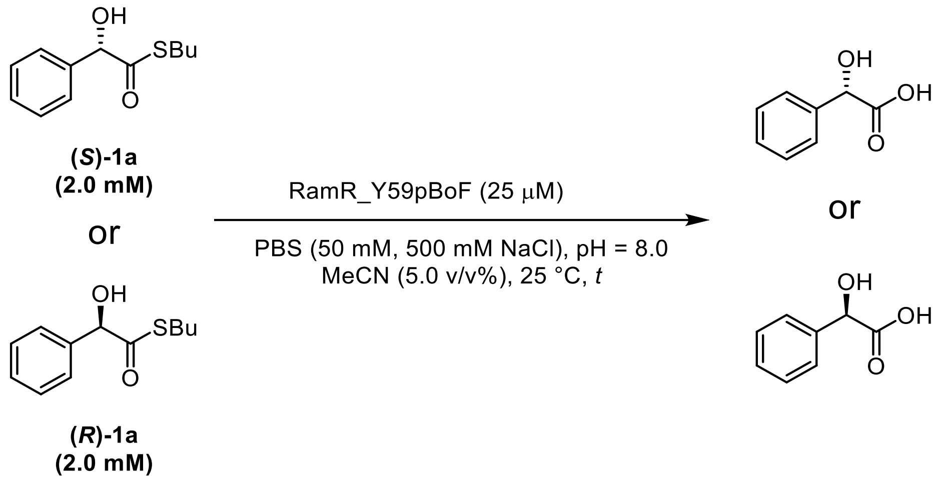

Entry	Substrate	Catalyst	Catalyst loading (μM)	TOF (h^{-1})	Fold increase
1	S	Parent	25	0.8 ± 0.1	-
2	R	Parent	25	20 ± 1	-
3	S	D152A	5.0	42 ± 25	51
4	R	D152A	5.0	619 ± 77	31
5	S	D152Q	5.0	6 ± 6	7.3
6	R	D152Q	5.0	42 ± 11	2.1
7	S	D152R	5.0	3 ± 1	3.4
8	R	D152R	5.0	65 ± 29	3.2

[a] Reaction conditions: **1a** (2.0 mM, 400 nmol, 1.0 equiv), catalyst (5 or 25 μM), PBS (50 mM, NaCl 500 mM, pH= 8.0), MeCN (5.0 %v/v), 25 °C. Conversion was monitored via UV/Vis spectroscopy at 237 nm. If not otherwise specified, the value reported is an average of two experiments conducted with biological duplicates. All error values are given as standard deviations. Turn-over frequency (TOF) was calculated by using initial rates for each enantiomer, corrected for background hydrolysis, divided by enzyme concentration.

SI.11 General considerations

Chemicals were purchased from commercial suppliers (*Sigma* (UK), *Acros* (Germany), *TCI* (Belgium/Japan), *abcr* (Germany), *BLD-Pharm* (Germany) and *Doug Discovery* (UK)) and used without further purification unless specified. Flash column chromatography was performed on silica gel (Silica-P flash silica gel from *Silicycle*, 0.040-0.063 mm, 230-400 mesh). The unnatural amino acid pBoF was purchased as the enantiopure freebase from *BLD-Pharm* (Germany). Lipase Type VII (CAS: 9001-62-1, EC no. 232-619-9 Product no. L1754), Amano Lipase PS (Product no. 534641) and Esterase from porcine liver (CAS: 9016-18-6, EC no. 232-773-7, Product no. E2884) were purchased from *sigma*. NMR ^{1}H-NMR and ^{13}C-NMR spectra were recorded on a *Varian* 400 (400 MHz) spectrometer in $CDCl_3$ or $(CD_3)_2SO$. Chemical shifts values (δ) are denoted in ppm using residual solvent peaks as the internal standard ($CHCl_3$: δ 7.26 for ^{1}H; 77.16 for ^{13}C. $(CD_3)_2SO$: δ 2.50 for ^{1}H; 39.52 for ^{13}C). ^{11}B-NMR spectroscopy was performed on a *Bruker* 600 (600 MHz) using the zgbs pulse sequence as reported by MacMillan and co-workers (at = 0.25 s, d1 = 0.25 s, n = 100000).[2, 3] The sample was prepared with concentrated protein solution via the addition of the internal standard ($NaBF_4$, δ= -1.4 ppm) in D_2O (final concentration of D_2O = 10%). For the 11B adduct studies, substrates were added in pH adjusted buffer for a final concentration of 10 mM. HPLC analysis of thioesters was conducted using a *Shimadzu* LC-10ADVP HPLC equipped with a *Shimadzu* SPD-M10AVP diode array detector. HPLC analysis of hydroxyacids was conducted on a Shimadzu LC-10AT HPLC with a Shimadzu SPD-M10A diode array S2 detector. The plasmids pEVOL-pBoF were gifts from Prof. Peter Schultz (The Scripps Research Institute)).[4] *E. coli* strains, NEB10β and BL21(DE3) (*New England Biolabs*) were used for cloning and expression. Primers were synthesized by *Eurofins MWG Operon* (Germany) and *Sigma-Aldrich* (UK). Plasmid Purification Kits were obtained from *QIAGEN* (Germany) and DNA sequencing carried out by *GATC-Biotech* (Germany). *Phusion* polymerase and DpnI were purchased from *New England Biolabs*. Strep-tactin columns (Strep-Tactin® Superflow® high capacity) and Desthiobiotin were purchased from *IBA-Lifesciences* (Germany). DENARASE® was purchased from *c-LEcta.* Concentrations of DNA and protein solutions were determined based on the absorption at 260 nm or 280 nm on a *Thermo Scientific* Nanodrop 2000 UV-Vis spectrophotometer. Molar extinction coefficients were approximated using the ProtParam Expasy web server https://web.expasy.org/protparam/. SFC analysis was performed using a *Waters* Acquity UPC2 system. High-Resolution Mass Spectrometry (HRMS) measurements of

small molecules were performed using a *Thermo* LTQ Orbitrap XL. Other Mass Spectrometry (MS) measurements were performed using a *Waters* Acquity H-class UPLC with *Waters* Xevo G2 QTOF.

SI.12 Additional information on the X-ray crystallography

Prior to crystallization, RamR_Y59pBoF was further purified by size exclusion chromatography on a Superdex™ 75 increase 10/300 GL (*Cytiva*) using 20 mM HEPES, 500 mM NaCl, pH 8.0 as eluent. Crystallization of RamR_Y59pBoF was performed with the PACT *premier*™ and JCGS-*plus*™ screens (Molecular Dimensions) using the sitting-drop vapor diffusion method at 21 °C. Drops of 200 nL were dispensed using a Mosquito robot (*SPTLabTech*), mixing protein solution (RamR_Y59pBoF: 13.5 mg/mL or 24.0 mg/mL in 20 mM HEPES, 500 mM NaCl, pH 8.0) with reservoir solutions at two volume ratio's (75:125 and 125:75) in MRC 96-Well 2-Drop crystallization plates. Crystals appeared at various conditions from both crystallization screens. Crystals of RamR_Y59pBoF obtained from varying conditions were cryo-protected by brief transfer to a solution composed of the respective crystallization condition with added NaCl up to 500 mM and 25% (v/v) PEG400 or glycerol, and subsequently flash cooled in liquid nitrogen. The crystal which gave the highest quality X-ray diffraction data was obtained with crystallization condition C8 from the JCGS-*plus*™ suite (0.1 M Tris pH 8.5, 20% ethanol), and was cryo-protected using a solution composed of the condition described above with an additional 500 mM NaCl and 25% (v/v) glycerol.

X-ray diffraction data were collected at 100 K at the ID30A-1 (MASSIF-1) beamline of the European Synchrotron Radiation Facility (ESRF) in Grenoble (wavelength X-rays 0.9655 Å). Initial data processing and analysis was performed using the automatic routines at the MASSIF-1 beamline.[5] The highest quality X-ray diffraction data set, exhibiting a maximum resolution of 2.78 Å, was selected for reprocessing and crystal structure determination. Unmerged data, obtained after indexing and integration by XDS[6], were scaled and merged with the Aimless routine[7] from the CCP4 software suite[8]. Initial phases and a starting structure were determined by molecular replacement with the program Phaser,[9] using a monomer of a previously published RamR structure (PDB: 3VVX)[10] as a search model. The structure of RamR_Y59pBoF belongs to space group $P2_1$ and contains four polypeptide chains in the asymmetric unit with a solvent content of 52.6%. Two of the polypeptide chains form a dimer in the same asymmetric unit, while the other two chains form dimers with polypeptide chains of a neighboring asymmetric unit. The final optimized structure was obtained via alternated rounds of model building and refinement using Coot[11] and Refmac5,[12] respectively. The structure was initially refined with a phenylalanine residue at position 59, resulting in clear

residual electron density for the boronic acid moiety. Subsequent refinement was performed with a pBoF residue at position 59, either in a free acid state (Phe-BO_2) or in a boronate anionic state (Phe-BO_3^-). The geometry dictionary file for the pBoF free acid state was obtained from the CCP4 library (residue code 78N), while for the boronate state it was calculated with the CCP4 AceDRG program.[13] The final structure was refined with a pBoF boronate residue in all four polypeptide chains in the asymmetric unit, using the program Phenix.refine.[14] Structure validation was performed with Molprobity[15] and using the wwPDB Validation Server (https://validate.wwpdb.org), ensuring that the final structure was of acceptable quality. Data collection and structure refinement statistics are summarized in Table S7. The structure of RamR_Y59pBoF was deposited in the Protein Data Bank with entry code 9GTV.

Table S7. **X-ray data collection and refinement statistics**

	RamR_Y59pBoF
**Data collection**	
Space group	$P2_1$
Cell dimensions	
a, b, c (Å), β (°)	78.8, 51.9, 118.3, 96.5
Resolution range (Å)	78 – 2.78
R_{meas}	0.091 (0.610)
R_{pim}	0.051 (0.399)
$CC_{1/2}$	0.996 (0.684)
Mean (I/σI)	8.5 (1.3)
Completeness (%)	93.4 (64.3)
Redundancy	2.9 (1.9)
**Refinement**	
Resolution range (Å)	78 – 2.78
No. reflections	22663
R-factor / R_{free} *	0.210 / 0.257
Content asymmetric unit	4 polypeptide chains (5874 non-H atoms), 4 pBoF residues (60 non-H atoms, boronate state)
B-factors (Å^2)	
protein, pBoF	93.2, 87.6
R.m.s. deviations	
bond lengths (Å)	0.0096
bond angles (°)	1.2
Ramachandran (%)	
favored, outliers	98.2, 0.0
Rotamer outliers (%)	4.7
Clashscore	11.5
PDB	9GTV

Values in parentheses are for the highest-resolution shell.
* The R-free is calculated using 5% of all reflections randomly chosen and excluded from structure factor calculation and refinement.

SI.13 Molecular biology procedures (Protein expression, mutagenesis, protein analytics)

Previously prepared plasmids pET17b+_LmrR_M8TAG[2], pET17b+_LmrR_A11TAG[2], pET17b+_LmrR_V15TAG[2], pET17b+_LmrR_M89TAG[2], pET17b+_LmrR_A92TAG[2], pET17b+_LmrR_F93TAG[2], pET17b+_LmrR_N19TAG[16], pET17b+_LmrR_K22TAG[16], pET17b+_QacR_Q96TAG[17], pET17b+_QacR_Y103[17], pET17b+_QacR_Y123[17], pET17b+_RamR_Y59[17], pET17b+_RamR_Y92[17], pET17b+_RamR_F155[17] were used in this study. Plasmids pET17b+_LmrR_L18TAG, pET17b+_LmrR_S97TAG, pET17b+_LmrR_D100TAG and pET17b+_LmrR_E107TAG were prepared using the Quikchange procedure in unpublished work. LmrR contains two point mutations in the DNA binding site (K55D_K59Q); native cysteine residues in QacR (C72A and C141S) and RamR (C67S and C134S) were substituted.

DNA Sequence of LmrR

```
GGTGCCGAAATCCCGAAAGAAATGCTGCGTGCTCAAACCAATGTCATCCTGCTGAATGTCCT
GAAACAAGGCGATAACTATGTGTATGGCATTATCAAACAGGTGAAAGAAGCGAGCAACGGTG
AAATGGAACTGAATGAAGCCACCCTGTATACGATTTTTGATCGTCTGGAACAGGACGGCATT
ATCAGCTCTTACTGGGGTGATGAAAGTCAAGGCGGTCGTCGCAAATATTACCGTCTGACCGA
AATCGGCCATGAAAACATGCGCCTGGCGGAAGAATCCTGGAGTCGTGTGGACAAAATCATTG
AAAATCTGGAAGCAAACAAAAAATCTGAAGCGATCAAATCTAGAGGTGGCAGCGGTGGCTGG
AGCCACCCGCAGTTCGAAAAA
```

DNA Sequence of QacR

```
CATATGAACCTGAAGGACAAAATCCTGGGTGTGGCGAAGGAGCTGTTCATTAAAAACGGTTA
TAACGCGACCACCACCGGCGAGATCGTTAAGCTGAGCGAAAGCAGCAAAGGCAACCTGTACT
ATCACTTCAAGACCAAAGAGAACCTGTTTCTGGAAATCCTGAACATTGAGGAAAGCAAGTGG
CAGGAGCAATGGAAAAGCGAACAGATTAAGTGCAAAACCAACCGTGAGAAGTTCTATCTGTA
CAACGAACTGAGCCTGACCACCCAGTACTATTACCCGCTGCAAAACGCGATCATCGAGTTCT
ACACCGAATACTACAAGACCAACAGCATCAACGAGAAGATGAACAAACTGGAAAACAAGTAT
ATCGACGCGTACCACGTGATTTTCAAAGAGGGTAACCTGAACGGCGAATGGTGCATTAACGA
TGTGAACGCGGTTAGCAAGATCGCGGCGAACGCGGTGAACGGTATTGTTACCTTTACCCACG
AGCAGAACATCAACGAACGTATTAAGCTGATGAACAAATTCAGCCAAATCTTTCTGAACGGC
CTGAGCAAGGCGGCGTGGAGCCACCCGCAATTTGAAAAATAACTCGAG
```

DNA/Amino Acid Sequence of RamR_Y59TAG

CATATGGTGGCGCGTCCGAAGAGCGAGGACAAGAAACAAGCGCTGCTGGAAGCGGCGACCCA
GGCGATTGCGCAAAGCGGTATTGCGGCGAGCACCGCGGTGATTGCGCGTAACGCGGGTGTTG
CGGAGGGTACCCTGTTCCGTTACTTTGCGACCAAGGACGAACTGATTAACACCCTGTAGCTG
CACCTGAAACAGGATCTGAGCCAAAGCATGATCATGGAGCTGGACCGTAGCATTACCGATGC
GAAAATGATGACCCGTTTCATCTGGAACAGCTACATTAGCTGGGGCCTGAACCATCCGGCGC
GTCACCGTGCGATCCGTCAGCTGGCGGTTAGCGAGAAGCTGACCAAAGAAACCGAACAACGT
GCGGACGATATGTTCCCGGAACTGCGTGATCTGAGCCACCGTAGCGTGCTGATGGTTTTTAT
GAGCGACGAGTACCGTGCGTTCGGTGATGGCCTGTTTCTGGCGCTGGCGGAAACCACCATGG
ATTTTGCGGCGCGTGATCCGGCGCGTGCGGGCGAGTATATTGCGCTGGGCTTTGAAGCGATG
TGGCGTGCGCTGACCCGTGAGGAACAGGCGGCGTGGAGCCACCCGCAATTTGAAAAGTAA

MVARPKSEDKKQALLEAATQAIAQSGIAASTAVIARNAGVAEGTLFRYFATKDELINTLpBo
FLHLKQDLSQSMIMELDRSITDAKMMTRFIWNSYISWGLNHPARHRAIRQLAVSEKLTKETE
QRADDMFPELRDLSHRSVLMVFMSDEYRAFGDGLFLALAETTMDFAARDPARAGEYIALGFE
AMWRALTREEQAAWSHPQFEK

List of Primers Used for Quikchange Mutagenesis

**Primer Name**	**Sequence (5'to 3')**
RamR_Y59TAG_L60A_fwd	CACCCTGTAGGCGCACCTGAAACAG
RamR_Y59TAG_L60A_rev	CTGTTTCAGGTGCGCCTACAGGGTG
RamR_Y59TAG_K63A_fwd	CTGCACCTGGCACAGGATCTGAGCCAA
RamR_Y59TAG_K63A_rev	TTGGCTCAGATCCTGTGCCAGGTGCAG
RamR_Y59TAG_L109A_fwd	ATCCGTCAGGCAGCGGTTAGCGAG
RamR_Y59TAG_L109A_rev	CTCGCTAACCGCTGCCTGACGGAT
RamR_Y59TAG_A110L_fwd	CCGTCAGCTGCTGGTTAGCGAGAAG
RamR_Y59TAG_A110L_rev	CTTCTCGCTAACCAGCAGCTGACGG
RamR_Y59TAG_E120A_fwd	AAAGAAACCGCACAACGTGCGGACGAT
RamR_Y59TAG_E120A_rev	ATCGTCCGCACGTTGTGCGGTTTCTTT
RamR_Y59TAG_A123L_fwd	CGAACAACGTCTGGACGATATGTTCCC
RamR_Y59TAG_A123L_rev	GGGAACATATCGTCCAGACGTTGTTCG
RamR_Y59TAG_D124A_fwd	CGTGCGGCGGATATGTTCCCGGAAC
RamR_Y59TAG_D124A_rev	GTTCCGGGAACATATCCGCCGCACG

RamR_Y59TAG_K63D_fwd	CACCTGGATCAGGATCTGAGCCAAAG
RamR_Y59TAG_K63D_rev	CCTGATCCAGGTGCAGCTACAGGG
RamR_Y59TAG_K63R_fwd	CACCTGCGCCAGGATCTGAGCCAAAG
RamR_Y59TAG_K63R_rev	CCTGGCGCAGGTGCAGCTACAGGG
RamR_Y59TAG_K63M_fwd	CACCTGATGCAGGATCTGAGCCAAAG
RamR_Y59TAG_K63M_rev	CCTGCATCAGGTGCAGCTACAGG
RamR_Y59TAG_K63N_fwd	CACCTGAACCAGGATCTGAGCCAAAG
RamR_Y59TAG_K63N_rev	CCTGGTTCAGGTGCAGCTACAGG
RamR_Y59TAG_D152A_fw	CGTTCGGTGCGGGCCTGTTTCTGGC
RamR_Y59TAG_D152A_rv	AACAGGCCCGCACCGAACGCACGGTAC
RamR_Y59TAG_D152C_fw	CGTTCGGTTGCGGCCTGTTTCTGGC
RamR_Y59TAG_D152C_rv	AACAGGCCGCAACCGAACGCACG
RamR_Y59TAG_D152E_fw	CGTTCGGTGAAGGCCTGTTTCTGGCG
RamR_Y59TAG_D152E_rv	AACAGGCCTTCACCGAACGCACGGTAC
RamR_Y59TAG_D152F_fw	CGTTCGGTTTTGGCCTGTTTCTGGCG
RamR_Y59TAG_D152F_rv	AACAGGCCAAAACCGAACGCACGG
RamR_Y59TAG_D152G_fw	CGTTCGGTGGCGGCCTGTTTCTGG
RamR_Y59TAG_D152G_rv	AACAGGCCGCCACCGAACGCAC
RamR_Y59TAG_D152H_fw	CGTTCGGTCATGGCCTGTTTCTG
RamR_Y59TAG_D152H_rv	AACAGGCCATGACCGAACGCAC
RamR_Y59TAG_D152I_fw	CGTTCGGTATTGGCCTGTTTCTGGC
RamR_Y59TAG_D152I_rv	AACAGGCCAATACCGAACGCACGG
RamR_Y59TAG_D152K_fw	CGTTCGGTAAAGGCCTGTTTCTGGCG
RamR_Y59TAG_D152K_rv	AACAGGCCTTTACCGAACGCACGG
RamR_Y59TAG_D152L_fw	CGTTCGGTCTGGGCCTGTTTCTGGC
RamR_Y59TAG_D152L_rv	AACAGGCCCAGACCGAACGCACG
RamR_Y59TAG_D152M_fw	CGTTCGGTATGGGCCTGTTTCTGGCG
RamR_Y59TAG_D152M_rv	AACAGGCCCATACCGAACGCACGG
RamR_Y59TAG_D152N_fw	CGTTCGGTAACGGCCTGTTTCTGG
RamR_Y59TAG_D152N_rv	AACAGGCCGTTACCGAACGCACGGTAC
RamR_Y59TAG_D152P_fw	CGTTCGGTCCGGGCCTGTTTCTG
RamR_Y59TAG_D152P_rv	AACAGGCCCGGACCGAACGCAC
RamR_Y59TAG_D152Q_fw	CGTTCGGTCAGGGCCTGTTTCTGGC

RamR_Y59TAG_D152Q_rv	AACAGGCCCTGACCGAACGCACG
RamR_Y59TAG_D152R_fw	CGTTCGGTCGCGGCCTGTTTCTGG
RamR_Y59TAG_D152R_rv	AACAGGCCGCGACCGAACGCAC
RamR_Y59TAG_D152S_fw	CGTTCGGTAGCGGCCTGTTTCTGGC
RamR_Y59TAG_D152S_rv	AACAGGCCGCTACCGAACGCACG
RamR_Y59TAG_D152T_fw	CGTTCGGTACCGGCCTGTTTCTGGC
RamR_Y59TAG_D152T_rv	AACAGGCCGGTACCGAACGCACG
RamR_Y59TAG_D152V_fw	CGTTCGGTGTGGGCCTGTTTCTGGC
RamR_Y59TAG_D152V_rv	AACAGGCCCACACCGAACGCACG
RamR_Y59TAG_D152W_fw	CGTTCGGTTGGGGCCTGTTTCTGGC
RamR_Y59TAG_D152W_rv	AACAGGCCCCAACCGAACGCACG
RamR_Y59TAG_D152Y_fw	CGTTCGGTTATGGCCTGTTTCTGGC
RamR_Y59TAG_D152Y_rv	AACAGGCCATAACCGAACGCACGG

Quikchange Mutagenesis to Prepare TAG Mutants

Reactions were conducted in 20 µL or 50 µL total volume containing Cloned *Pfu* reaction buffer (1X final concentration), dNTPs mixture (0.2 mM each dNTP), DNA template (based on pET17b+, 5.0 ng/µL), primers (2.5 ng/µL), DMSO (3 % v/v) and *PfuTurbo* DNA polymerase (2.5 U). The reactions were then briefly centrifuged and transferred to a thermocycler (*BioRad*) and were then subject to the following steps: initial denaturation (98 °C, 30 seconds), then 20-25 cycles of (1) denaturation (98 °C, 10 seconds) (2) annealing (specified temperature, 30 seconds) (3) extension (72 °C, 120 seconds), followed by a final extension (72 °C, 10 minutes).

**PCR protocol 1**		
**Temperature**	**Time**	**No. of cycles**
98 °C	30 seconds	1
98 °C	10 seconds	20-25x
64 °C – 0.2 °C / cycle	30 seconds	
72 °C	120 seconds	
72 °C	10 minutes	1

**PCR protocol 2**		
**Temperature**	**Time**	**No. of cycles**
99 °C	1 minute	1
98 °C	15 seconds	20-25x
52 °C + 0.3 °C / cycle	30 seconds	
72 °C	120 seconds	
72 °C	10 minutes	1

**PCR protocol 3**		
**Temperature**	**Time**	**No. of cycles**
99 °C	1 minute	1
98 °C	30 seconds	15x
70 °C – 1.0 °C / cycle	30 seconds	
72 °C	120 seconds	
98 °C	30 seconds	10x
58 °C	30 seconds	
72 °C	120 seconds	
72 °C	10 minutes	1

Representative Procedure for Plasmid Amplification, Isolation and Transformation

A 50 µL aliquot of chemically competent *E. Coli* NEB10β cells prepared using a protocol adapted from Yang et al. was supplied with 10 µL KCM buffer (500 mM KCl, 150 mM $CaCl_2$, 250 mM $MgCl_2$), 25 µL MilliQ and 15 µL of PCR product and incubated on ice for 30 min.[18] Cells were heat-shocked at 42 °C for 40 s before being supplied with 600 µL Super Optimal Broth medium and recovering for 1 h (37 °C, 600 rpm). The cell culture (100 µL) was then spread on Luria–Bertani medium agar laced with ampicillin and incubated at 37 °C overnight. Single colonies were used to inoculate 5 mL of Luria–Bertani medium containing ampicillin in a culture tube which was then incubated overnight (37 °C, 180 rpm). Purified plasmids were obtained using *QIAGEN* Plasmid Purification kits. pET17b+_RamR mutant plasmid (3.0 µL) was added to a 30 µL aliquot of chemically competent *E. Coli* BL21-DE3 cells harboring the pEVOL_pBoF plasmid, and incubated on ice for 30 min. Cells were heat-shocked and recovered as described above, and 100 µL of cell culture was spread on Luria–Bertani medium agar laced with ampicillin and chloramphenicol and incubated at 37 °C overnight. Single colonies were picked to inoculate 5.0 mL of Luria–Bertani medium containing ampicillin and chloramphenicol in a culture tube which was then incubated overnight (37 °C, 180 rpm). A glycerol stock was then prepared by combining 500 µL of dense preculture with 500 µL of 50% glycerol and storing at -70 °C.

Representative Procedure for Protein Expression and Purification

A glycerol stock or single colony of *E. coli* BL21-DE3 cells harboring pET17b+_Protein with stop codon in the relevant position, as well as any other desired mutations, as well as the pEVOL_pBoF plasmid were used to inoculate 5 mL of Luria–Bertani medium containing ampicillin and chloramphenicol in a culture tube which was then incubated overnight (37 °C, 135 rpm). The dense culture (1.0 mL) was then used to inoculate fresh Terrific Broth medium (100 mL) with the same antibiotics in a 500 mL Erlenmeyer flask. The culture was incubated (37 °C, 135 rpm) until OD_{600} ~ 0.4 when pBoF and arabinose were added to final concentrations of 1 mM and 0.2 %, respectively. The culture was further incubated (37 °C, 135 rpm) until OD_{600} ~ 1 when protein expression was induced with the addition of IPTG (1 mM final concentration). The culture was incubated overnight (30 °C, 135 rpm) and the cells were harvested by centrifugation (4 °C, 6000 rpm, 20 minutes) and resuspended with Buffer X (50 mM PBS, 500 mM NaCl, pH = 8.0, 10 mL) and DENARASE® (*c-LEcta*) was added (~100 U final concentration). The cells were disrupted by sonication, and the cell debris removed by

centrifugation (4 °C, 12000 rpm, 45 minutes). The supernatant was passed through a syringe-filter (0.2 µm, *Whatman*) and applied to Strep-tag II resin (*IBA Lifesciences*) (4 mL). The resin was then washed with Buffer X (3 × 8 mL) and Buffer X containing higher NaCl concentration (50 mM PBS, 1 M NaCl, pH = 8.0, 10 mL). The protein was eluted with Buffer X containing desthiobiotin (*IBA Lifesciences*, 5 mM, 12 mL). The protein was concentrated to 0.5 mL with a centrifugal filter and exchanged into the buffer used for catalysis (50 mM PBS, 500 mM NaCl, pH = 8.0) with a desalting column (*GE Healthcare*). The protein concentration was determined by absorbance at 280 nm measured with a Nanodrop 2000 (*Thermo Scientific*) with extinction coefficients estimated using the expasy webserver: https://web.expasy.org/protparam/.

Confirmation of Protein Identity with LCMS

UPLC/MS analysis was performed on *Waters* Acquity Ultra Performance LC with Xevo G2 QTOF detector. Water (solvent A) and acetonitrile (solvent B) containing 0.1% formic acid by volume, were used as the mobile phase at a flow rate of 0.3 mL/min. Gradient: 90% A for 2 min, linear gradient to 50% A in 2 min, linear gradient to 20% A in 5 min, followed by 2 min at 5% A. Re-equilibration of the column with 2 min at 90% A.

Procedure for adduct study via direct injection HRMS

The respective protein (20 µM) in 500 µL NH_4HCO_3 buffer (50 mM, pH = 7.8) was incubated with the substrate dissolved in DMF (50 equiv, 1.0 mM final concentration, 20 mM stock solution in DMF) for 1 h on ice. The solution was then injected into a *Waters* Xevo G2 QTOF with a syringe pump over 5 min. Capillary voltage (3.5 kV), sampling core temperature (40 °C), source temperature (120 °C), desolvation temperature (450 °C), Cone gas (20 L/h), desolvation flow (700 L/h).

List of purified proteins

Wild type RamR	RamR_Y59pBoF	RamR_Y59pBoF_D152F
LmrR_M8pBoF[2]	RamR_Y92pBoF	RamR_Y59pBoF_D152G
LmrR_A11pBoF[2]	RamR_F155pBoF	RamR_Y59pBoF_D152H
LmrR_V15pBoF[2]	RamR_Y59pBoF_L60A	RamR_Y59pBoF_D152I
LmrR_M89pBoF[2]	RamR_Y59pBoF_K63A	RamR_Y59pBoF_D152K
LmrR_A92pBoF[2]	RamR_Y59pBoF_L109A	RamR_Y59pBoF_D152L
LmrR_F93pBoF[2]	RamR_Y59pBoF_A110L	RamR_Y59pBoF_D152M
LmrR_L18pBoF	RamR_Y59pBoF_E120A	RamR_Y59pBoF_D152N
LmrR_N19pBoF	RamR_Y59pBoF_A123L	RamR_Y59pBoF_D152P
LmrR_K22pBoF	RamR_Y59pBoF_D124A	RamR_Y59pBoF_D152Q
LmrR_S97pBoF	RamR_Y59pBoF_D152A	RamR_Y59pBoF_D152R
LmrR_D100pBoF	RamR_Y59pBoF_K63M	RamR_Y59pBoF_D152S
LmrR_E107pBoF	RamR_Y59pBoF_K63N	RamR_Y59pBoF_D152T
QacR_Q96pBoF	RamR_Y59pBoF_K63R	RamR_Y59pBoF_D152V
QacR_Y103pBoF	RamR_Y59pBoF_D152C	RamR_Y59pBoF_D152W
QacR_Y123pBoF	RamR_Y59pBoF_D152E	RamR_Y59pBoF_D152Y

Representative yields for different boron designer enzymes

LmrR_M8pBoF	54 mg/L
LmrR_V15pBoF	39 mg/L
LmrR_L18pBoF	81 mg/L
LmrR_N19pBoF	92 mg/L
LmrR_K22pBoF	136 mg/L
LmrR_M89pBoF	26 mg/L
LmrR_S97pBoF	63 mg/L
LmrR_D100pBoF	346 mg/L
LmrR_E107pBoF	93 mg/L
QacR_Q96pBoF	14 mg/L
QacR_Y103pBoF	2 mg/L
QacR_Y123pBoF	48 mg/L
RamR_Y59pBoF	47 mg/L
RamR_Y92pBoF	80 mg/L

RamR_F155pBoF	40 mg/L

SI.14 Synthetic chemistry (General procedures, substrate synthesis, reaction analysis)

General procedures for the biocatalytic kinetic resolution reaction

General procedure GP1 for the evaluation of artificial enzymes in the conversion of thioesters: To a 2.0 mL Eppendorf plastic tube was added an artificial enzyme in PBS buffer (50 mM, NaCl = 500 mM, pH = 8.0) with a total volume of 190 μL. Thioester **1** in MeCN (10 μL, 200 nmol, 20 mM stock, 1.0 mM final concentration) was added. The reaction mixture was gently shaken (450 rpm) at 25 °C for 16 h. 2-Phenyl quinoline in MeCN (10 μL, 40 nmol, 4 mM stock) was added and the reaction mixture was extracted with *n*BuOH (500 μL). After strong mixing and short centrifugation, the organic layer (400 μL) was separated and washed with H_2O (300 μL). The organic layer (200 μL) was then dried using anhydrous Na_2SO_4 and directly analyzed using HPLC (injection volume 20 μL) or SFC (injection volume 5.0 μL). The absolute configuration of starting material was determined by comparison with an authentic commercial sample via SFC. Other substrates are assumed to convert in a similar manner. When the peak area of an enantiomer in the chromatogram was below the detection limit, an area of 1000 was used (estimated limit of detection ~0.5% yield).

Synthetic procedures and characterization data of synthesized substrates

Substrate **1i** is commercially available.

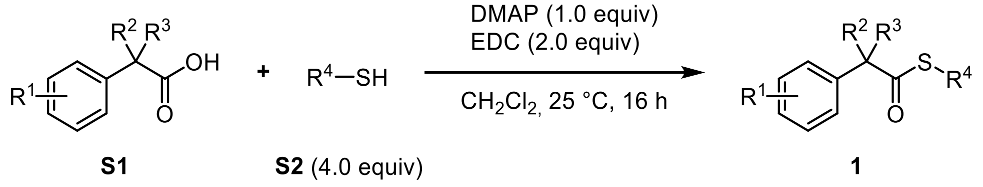

Scheme S2. Reaction scheme for the synthesis of substituted thioesters **1**.

General procedure GP2 for the formation of thioesters: A 100 mL round bottom flask was charged with the carboxylic acid substrate (**S1**, 1.0 equiv, 3.0 mmol scale) and 4-dimethylaminopyridine (DMAP, 1.0 equiv). CH_2Cl_2 was added (0.5 M of substrate, 6.0 mL) and the respective thiol **S2** (4.0 equiv) were added. Under strong stirring, 1-ethyl-3-(3-dimethylaminopropyl)carbodiimide (EDC, 2.0 equiv) was added portion wise and the reaction was stirred at room temperature for 16 h. After completion of the reaction time, the mixture

was diluted with CH_2Cl_2 and immediately loaded on SiO2 by evaporation under reduced pressure. The crude products **1** were subsequently purified via column chromatography (SiO_2, EtOAc/heptane).

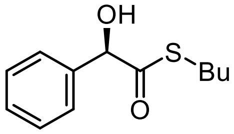

The substrate (*R*)-**1a** was synthesized following general procedure **GP2** employing (*R*)-mandelic acid (760 mg, 5.00 mmol, 1.0 equiv), BuSH (2.1 mL, 1.80 g, 20 mmol, 4.0 equiv), DMAP (61 mg, 0.5 mmol, 0.1 equiv) and EDC (1.91 g, 10 mmol, 2.0 equiv) at room temperature for 16 h. The target product S-butyl (*R*)-2-hydroxy-2-phenylethanethioate ((*R*)-**1a**, 265 mg, 1.18 mmol, 24%) was isolated using column chromatography (SiO_2, pentane/EtOAc = 20:1) as a colorless oil. Spectroscopic data is matching with previous reported data in the literature.[19] ^1H NMR (600 MHz, $CDCl_3$, 25 °C) δ= 7.46 – 7.29 (m, 5H), 5.22 (d, J = 4.5 Hz, 1H), 3.55 (d, J = 4.6 Hz, 1H), 3.00 – 2.77 (m, 2H), 1.57 – 1.46 (m, 2H), 1.41 – 1.27 (m, 2H), 0.89 (t, J = 7.3 Hz, 3H) ppm.

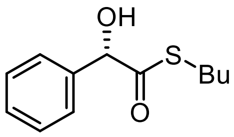

The substrate (*S*)-**1a** was synthesized following general procedure **GP2** employing (*S*)-mandelic acid (1.52 g, 10.0 mmol, 1.0 equiv), BuSH (2.1 mL, 1.80 g, 20 mmol, 2.0 equiv), DMAP (122 mg, 1.0 mmol, 0.1 equiv) and EDC (3.83 g, 20 mmol, 2.0 equiv) at room temperature for 3 h. The target product S-butyl (*S*)-2-hydroxy-2-phenylethanethioate ((*S*)-**1a**, 59 mg, 0.26 mmol, 3%) was isolated using column chromatography (SiO_2, pentane/EtOAc = 20:1) as a colorless oil. Spectroscopic data is matching with previous reported data in the literature.[19] ^1H NMR (600 MHz, $CDCl_3$, 25 °C) δ= 7.46 – 7.29 (m, 5H), 5.22 (d, J = 4.5 Hz, 1H), 3.55 (d, J = 4.6 Hz, 1H), 3.00 – 2.77 (m, 2H), 1.57 – 1.46 (m, 2H), 1.41 – 1.27 (m, 2H), 0.89 (t, J = 7.3 Hz, 3H) ppm.

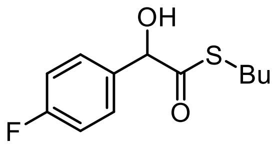

The substrate **1b** was synthesized following general procedure **GP2** employing 2-(4-fluorophenyl)-2-hydroxyacetic acid (510 mg, 3.00 mmol, 1.0 equiv), BuSH (1.3 mL, 1.08 g, 12 mmol, 4.0 equiv), DMAP (366 mg, 3.0 mmol, 1.0 equiv) and EDC (1.15 g, 6.0 mmol, 2.0 equiv) at room temperature for 16 h. The target product S-butyl 2-(4-fluorophenyl)-2-hydroxyethanethioate (**1b**, 341 mg, 1.41 mmol, 47%) was isolated using column chromatography (SiO_2, pentane/EtOAc = 10:1) as a colorless oil. ^1H NMR (600 MHz, $CDCl_3$, 25 °C) δ= 7.43 – 7.32 (m, 2H), 7.11 – 6.99 (m, 2H), 5.16 (s, 1H), 3.81 (s, 1H), 2.96 – 2.69 (m, 2H), 1.57 – 1.45 (m,

2H), 1.41 – 1.27 (m, 2H), 0.88 (t, J = 7.4 Hz, 3H) ppm. ^{13}C NMR (151 MHz, $CDCl_3$, 25 °C) δ = 202.2, 163.8, 162.2, 134.1, 134.1, 128.9, 128.8, 115.7, 115.6, 79.2, 31.2, 28.7, 21.9, 13.5. ppm. HRMS (ESI) *m/z* for $C_{12}H_{15}FO_2S$ $[M+Na]^+$, calcd 265.0669, found 265.0666.

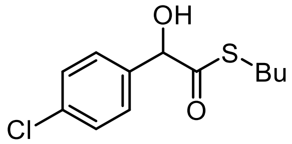

The substrate **1c** was synthesized following general procedure **GP2** employing 2-(4-chlorophenyl)-2-hydroxyacetic acid (560 mg, 3.00 mmol, 1.0 equiv), BuSH (1.3 mL, 1.08 g, 12 mmol, 4.0 equiv), DMAP (366 mg, 3.0 mmol, 1.0 equiv) and EDC (1.15 g, 6.0 mmol, 2.0 equiv) at room temperature for 16 h. The target product S-butyl 2-(4-chlorophenyl)-2-hydroxyethanethioate (**1c**, 145 mg, 0.56 mmol, 19%) was isolated using column chromatography (SiO_2, pentane/EtOAc = 10:1) as a colorless oil. Spectroscopic data is matching with previous reported data in the literature.[19] ^{1}H NMR (600 MHz, $CDCl_3$, 25 °C) δ = 7.38 – 7.31 (m, 4H), 5.16 (s, 1H), 3.64 (s, 1H), 2.88 (dt, J = 11.6, 7.4 Hz, 2H), 1.58 – 1.48 (m, 2H), 1.42 – 1.27 (m, 2H), 0.89 (t, J = 7.4 Hz, 3H) ppm. ^{13}C NMR (151 MHz, $CDCl_3$, 25 °C) δ = 201.8, 136.7, 134.8, 129.0, 128.4, 79.3, 31.3, 28.8, 22.0, 13.6 ppm. HRMS (ESI) *m/z* for $C_{12}H_{15}ClO_2S$ $[M+Na]^+$, calcd 281.0374, found 281.0372.

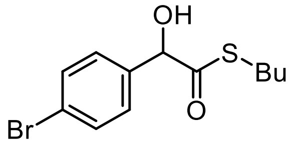

The substrate **1d** was synthesized following general procedure **GP2** employing 2-(4-bromophenyl)-2-hydroxyacetic acid (693 mg, 3.00 mmol, 1.0 equiv), BuSH (1.3 mL, 1.08 g, 12 mmol, 4.0 equiv), DMAP (366 mg, 3.0 mmol, 1.0 equiv) and EDC (1.15 g, 6.0 mmol, 2.0 equiv) at room temperature for 16 h. The target product S-butyl 2-(4-bromophenyl)-2-hydroxyethanethioate (**1d**, 145 mg, 0.48 mmol, 16%) was isolated using column chromatography (SiO_2, pentane/EtOAc = 10:1) as a colorless oil. ^{1}H NMR (600 MHz, $CDCl_3$, 25 °C) δ = 7.49 (d, J = 8.5 Hz, 2H), 7.28 (d, J = 8.4 Hz, 2H), 5.15 (s, 1H), 3.63 (s, 1H), 3.00 – 2.75 (m, 2H), 1.55 – 1.46 (m, 2H), 1.41 – 1.28 (m, 2H), 0.89 (t, J = 7.4 Hz, 3H) ppm. ^{13}C NMR (151 MHz, $CDCl_3$, 25 °C) δ = 201.7, 137.3, 131.9, 128.8, 123.0, 79.3, 31.3, 28.8, 22.0, 13.6 ppm. HRMS (ESI) *m/z* for $C_{12}H_{15}BrO_2S$ $[M+Na]^+$, calcd 324.9868, found 324.9867.

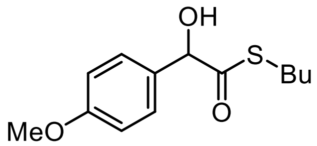

The substrate **1e** was synthesized following general procedure **GP2** employing 2-(4-methoxyphenyl)-2-hydroxyacetic acid (547 mg, 3.00 mmol, 1.0 equiv), BuSH (1.3 mL, 1.08 g, 12 mmol, 4.0 equiv), DMAP (366 mg, 3.0 mmol, 1.0 equiv) and EDC (1.15 g, 6.0 mmol, 2.0 equiv) at room temperature for 16 h. The target product S-butyl 2-(4-methoxyphenyl)-2-hydroxyethanethioate

(**1e**, 217 mg, 0.85 mmol, 28%) was isolated using column chromatography (SiO_2, pentane/EtOAc = 10:1) as a colorless oil. ^{1}H NMR (600 MHz, $CDCl_3$, 25 °C) δ = 7.31 (d, J = 8.7 Hz, 2H), 6.88 (d, J = 8.8 Hz, 2H), 5.12 (s, 1H), 3.79 (s, 3H), 3.66 (s, 1H), 2.96 – 2.79 (m, 2H), 1.59 – 1.47 (m, 2H), 1.39 – 1.27 (m, 3H), 0.89 (t, J = 7.4 Hz, 4H) ppm. ^{13}C NMR (151 MHz, $CDCl_3$, 25 °C) δ = 202.4, 160.0, 130.5, 128.5, 114.2, 79.5, 55.3, 31.3, 28.7, 22.0, 13.6 ppm. HRMS (ESI) *m/z* for $C_{13}H_{18}O_3S$ $[M+Na]^+$, calcd 277.0869, found 277.0870.

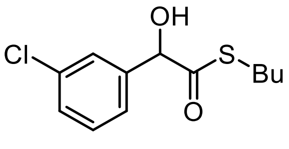

The substrate **1f** was synthesized following general procedure **GP2** employing 2-(3-chlorophenyl)-2-hydroxyacetic acid (560 mg, 3.00 mmol, 1.0 equiv), BuSH (1.3 mL, 1.08 g, 12 mmol, 4.0 equiv), DMAP (366 mg, 3.0 mmol, 1.0 equiv) and EDC (1.15 g, 6.0 mmol, 2.0 equiv) at room temperature for 16 h. The target product S-butyl 2-(3-chlorophenyl)-2-hydroxyethanethioate (**1f**, 479 mg, 1.85 mmol, 62%) was isolated using column chromatography (SiO_2, pentane/EtOAc = 10:1) as a colorless oil. ^{1}H NMR (600 MHz, $CDCl_3$, 25 °C) δ = 7.41 (s, 1H), 7.34 – 7.27 (m, 3H), 5.15 (s, 1H), 3.81 (s, 1H), 2.98 – 2.80 (m, 2H), 1.57 – 1.48 (m, 2H), 1.40 – 1.30 (m, 2H), 0.88 (t, J = 7.4 Hz, 3H) ppm. ^{13}C NMR (151 MHz, $CDCl_3$, 25 °C) δ = 201.6, 140.2, 134.7, 130.0, 129.0, 127.2, 125.3, 79.3, 31.2, 28.8, 22.0, 13.6 ppm. HRMS (ESI) *m/z* for $C_{12}H_{15}ClO_2S$ $[M+Na]^+$, calcd 281.0374, found 281.0375.

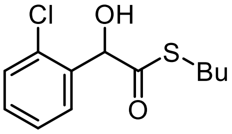

The substrate **1g** was synthesized following general procedure **GP2** employing 2-(2-chlorophenyl)-2-hydroxyacetic acid (560 mg, 3.00 mmol, 1.0 equiv), BuSH (1.3 mL, 1.08 g, 12 mmol, 4.0 equiv), DMAP (366 mg, 3.0 mmol, 1.0 equiv) and EDC (1.15 g, 6.0 mmol, 2.0 equiv) at room temperature for 16 h. The target product S-butyl 2-(2-chlorophenyl)-2-hydroxyethanethioate (**1g**, 186 mg, 0.72 mmol, 24%) was isolated using column chromatography (SiO_2, pentane/EtOAc = 10:1) as a colorless oil. ^{1}H NMR (600 MHz, $CDCl_3$, 25 °C) δ = 7.42 – 7.37 (m, 2H), 7.30 – 7.25 (m, 2H), 5.63 (s, 1H), 3.81 (s, 1H), 2.98 – 2.80 (m, 2H), 1.58 – 1.50 (m, 2H), 1.43 – 1.30 (m, 2H), 0.89 (t, J = 7.4 Hz, 3H) ppm. ^{13}C NMR (151 MHz, $CDCl_3$, 25 °C) δ = 201.3, 136.2, 133.8, 130.1, 130.0, 129.3, 127.3, 76.9, 31.3, 28.8, 22.0, 13.6ppm. HRMS (ESI) *m/z* for $C_{12}H_{15}ClO_2S$ $[M+Na]^+$, calcd 281.0374, found 281.0372.

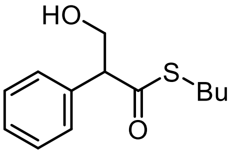

The substrate **1j** was synthesized following general procedure **GP2** employing 3-hydroxy-2-phenylpropanoic acid (499 mg, 3.00 mmol, 1.0 equiv), BuSH (1.3 mL, 1.08 g, 12 mmol, 4.0 equiv), DMAP (366 mg,

3.0 mmol, 1.0 equiv) and EDC (1.15 g, 6.0 mmol, 2.0 equiv) at room temperature for 16 h. The target product S-butyl 3-hydroxy-2-phenylpropanethioate (**1j**, 311 mg, 1.31 mmol, 44%) was isolated using column chromatography (SiO_2, pentane/EtOAc = 10:1) as a colorless oil. ^1H NMR (600 MHz, $CDCl_3$, 25 °C) δ = 7.45 – 7.28 (m, 5H), 4.22 (dd, J = 11.2, 8.3 Hz, 1H), 4.03 (dd, J = 8.2, 5.4 Hz, 1H), 3.85 (dd, J = 11.2, 5.4 Hz, 1H), 2.95 – 2.79 (m, 2H), 2.05 (s, 1H), 1.61 – 1.43 (m, 2H), 1.35 (h, J = 7.4 Hz, 2H), 0.89 (t, J = 7.4 Hz, 3H) ppm. ^{13}C NMR (151 MHz, $CDCl_3$, 25 °C) δ = 200.8, 135.7, 129.0, 128.7, 128.1, 64.7, 62.4, 31.5, 29.0, 22.1, 13.7 ppm. HRMS (ESI) *m/z* for $C_{13}H_{18}O_2S$ $[M+Na]^+$, calcd 261.0920, found 261.0920.

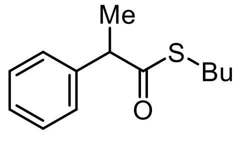

The substrate **1k** was synthesized following general procedure **GP2** employing 2-phenylpropanoic acid (451 mg, 3.00 mmol, 1.0 equiv), BuSH (1.3 mL, 1.08 g, 12 mmol, 4.0 equiv), DMAP (366 mg, 3.0 mmol, 1.0 equiv) and EDC (1.15 g, 6.0 mmol, 2.0 equiv) at room temperature for 16 h. The target product S-butyl 2-phenylpropanethioate (**1k**, 536 mg, 2.41 mmol, 80%) was isolated using column chromatography (SiO_2, pentane/EtOAc = 10:1) as a colorless oil. ^1H NMR (600 MHz, $CDCl_3$, 25 °C) δ = 7.36 – 7.27 (m, 5H), 3.88 (q, J = 7.1 Hz, 1H), 2.93 – 2.75 (m, 2H), 1.53 (d, J = 7.1 Hz, 3H), 1.52 – 1.47 (m, 2H), 1.39 – 1.30 (m, 2H), 0.88 (t, J = 7.4 Hz, 3H) ppm. ^{13}C NMR (151 MHz, $CDCl_3$, 25 °C) δ = 201.4, 140.2, 128.8, 128.0, 127.5, 54.4, 31.6, 28.9, 22.1, 18.6, 13.7 ppm. HRMS (APCI) *m/z* for $C_{13}H_{18}OS$ $[M+H]^+$, calcd 223.1151, found 223.1152.

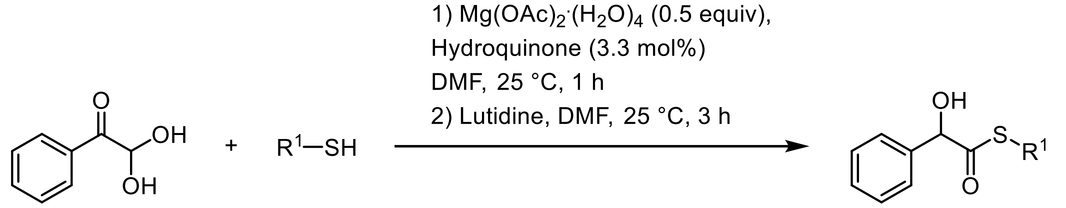

Scheme S3. Synthesis of substrate **1a and 1h**.[19]

Synthesis of **1a**: To a 100 mL round bottom flask was added 2,2-dihydroxy-1-phenylethan-1-one (2.01 g, 15.0 mmol, 1.0 equiv), $Mg(OAc)_2$ tetrahydrate (1.61 g, 7.5 mmol, 0.5 equiv) and DMF (20 mL). The mixture was stirred at room temperature for 15 min and BuSH (3.2 mL, 2.71 g, 30.0 mmol, 2.0 equiv) and hydroquinone (55 mg, 0.50 mmol, 3.3 mol%) were added. The reaction mixture was stirred for 1 h at room temperature and a solution of 2,6-dimethylpyridine (1.61 g, 15.0 mmol, 1.0 equiv) in DMF (5.0 mL) was added. The reaction mixture was stirred for 3 h at room temperature and then quenched with brine (50 mL) and Et_2O

(50 mL). The organic phase was dried with anhydrous Na_2SO_4 and all volatiles were removed in vacuo. The target product S-butyl 2-hydroxy-2-phenylethanethioate (**1a**, 2.33 g, 10.4 mmol, 69%) was isolated using column chromatography (SiO_2, pentane/EtOAc = 10:1) as a light yellow oil. Spectroscopic data is matching with previous reported data in the literature.[19] ^{1}H NMR (600 MHz, $CDCl_3$, 25 °C) δ = 7.46 – 7.29 (m, 5H), 5.22 (d, J = 4.5 Hz, 1H), 3.55 (d, J = 4.6 Hz, 1H), 3.00 – 2.77 (m, 2H), 1.57 – 1.46 (m, 2H), 1.41 – 1.27 (m, 2H), 0.89 (t, J = 7.3 Hz, 3H) ppm.

Synthesis of **1h**: To a 50 mL round bottom flask was added 2,2-dihydroxy-1-phenylethan-1-one (500 mg, 3.3 mmol, 1.0 equiv), $Mg(OAc)_2$ tetrahydrate (352 mg, 1.6 mmol, 0.5 equiv) and DMF (4 mL). The mixture was stirred at room temperature for 15 min and a solution of *tert*-BuSH (0.74 mL, 592 mg, 6.6 mmol, 2.0 equiv) and hydroquinone (12 mg, 0.11 mmol, 3.3 mol%) in DMF (0.5 mL). The reaction mixture was stirred for 1 h at room temperature and a solution of 2,6-dimethylpyridine (352 mg, 3.0 mmol, 1.0 equiv) in DMF (0.5 mL) was added. The reaction mixture was stirred for 3 h at room temperature and then quenched with brine (5.0 mL) and Et_2O (10 mL). The aqueous phase was extracted with Et_2O (2 × 10 mL). The combined organic phase was dried with anhydrous Na_2SO_4 and all volatiles were removed in vacuo. The target product S-(*tert*-butyl) 2-hydroxy-2-phenylethanethioate (**1h**, 273 mg, 1.2 mmol, 37%) was isolated using column chromatography (SiO_2, pentane/EtOAc = 20:1 to 10:1) as a colorless oil. Spectroscopic data is matching with previous reported data in the literature.[19] ^{1}H NMR (400 MHz, $CDCl_3$, 25 °C) δ = 7.42 – 7.32 (m, 5H), 5.11 (s, 1H), 3.73 (s, 1H), 1.45 (s, 9H) ppm.

HPLC and SFC methods

Methods for the SFC using $scCO_2$ and MeOH or *i*PrOH as eluents. Flowrates are 1.8 mL/min. High performance liquid chromatography (HPLC) was performed using a Shimadzu LC-10AT HPLC with a Shimadzu SPD-M10A diode array S2 detector. The columns used in the SFC were (*R*,*R*)Whelk-01 (*Regis Technologies*), Trefoil AMY1 (*Waters*), Trefoil CEL2 (*Waters*). The column used in the HPLC was a Chiralpak AD-H (*Daicel Chiral Technologies*). The column used for the separation of hydroxyacid **2a** was 3126 (D)-penicillamine column (250 × 4 mm, *Chirex*).

Flowrate 1.8 mL/min, $scCO_2$/MeOH

**Method 1**		
t / min	%CO_2	%MeOH
0	97	3
6	80	20
8	50	50
10	50	50

**Method 2**		
t / min	%CO_2	%MeOH
0	97	3
5	97	3
7	90	10
8	50	50
10	50	50

**Method 3**		
t / min	%CO_2	%MeOH
0	97	3
3	55	45
3	97	3
4	97	3

SI.15 HPLC and SFC chromatograms

1a

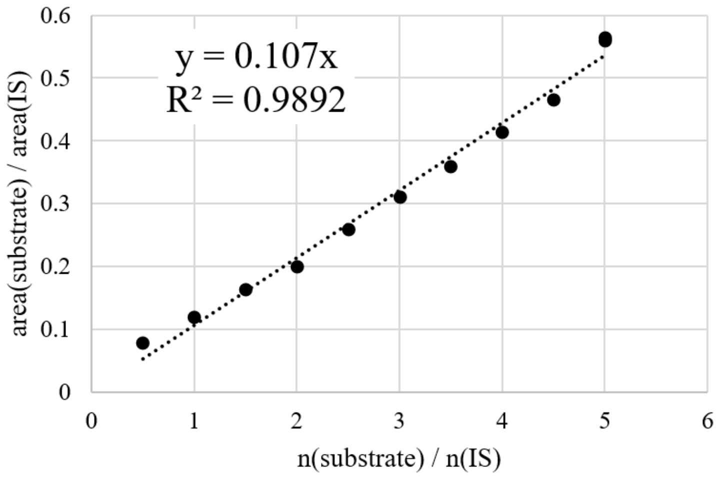

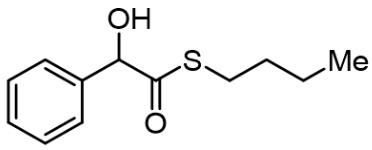

S-butyl 2-hydroxy-2-phenylethanethioate
(*R*,*R*)Whelk-01, method 3, 245 nm
enantiomer A = 1.65 min,
enantiomer B = 1.81 min

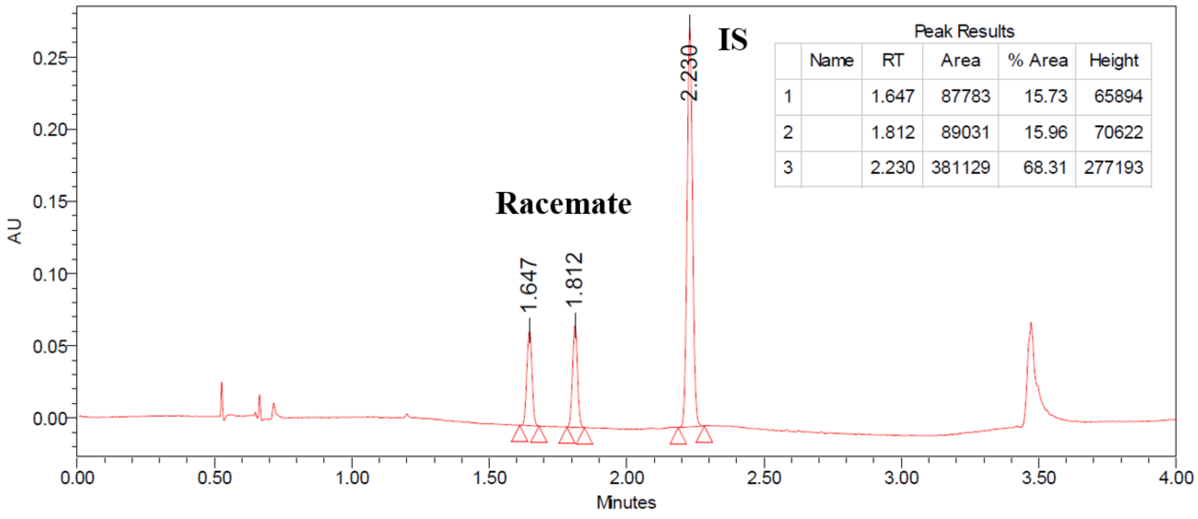

Peak Results

	Name	RT	Area	% Area	Height
1		1.647	87783	15.73	65894
2		1.812	89031	15.96	70622
3		2.230	381129	68.31	277193

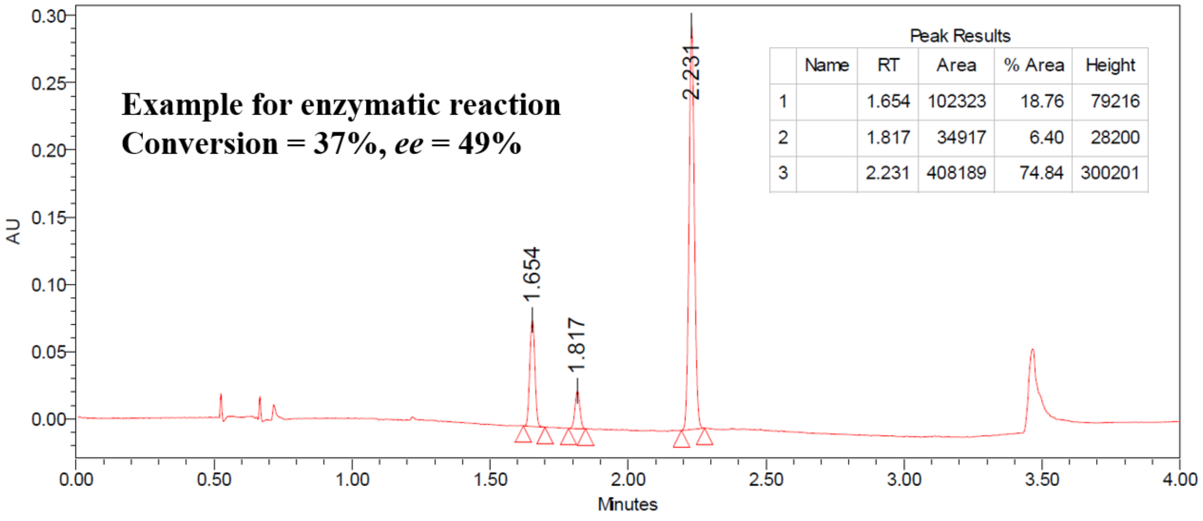

Peak Results

	Name	RT	Area	% Area	Height
1		1.654	102323	18.76	79216
2		1.817	34917	6.40	28200
3		2.231	408189	74.84	300201

1b

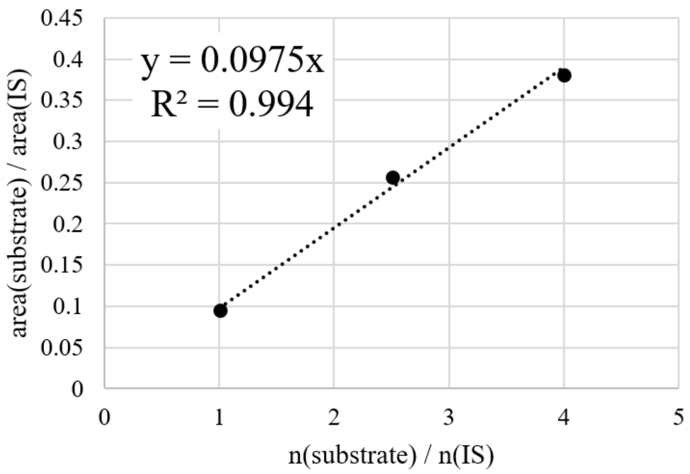

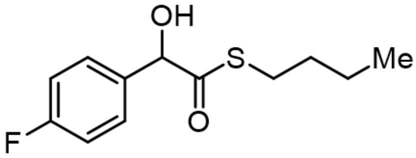

S-butyl 2-(4-fluorophenyl)-2-hydroxyethanethioate
(*R*,*R*)Whelk-01, method 1, 245 nm
enantiomer A = 1.63 min, enantiomer B = 1.83 min

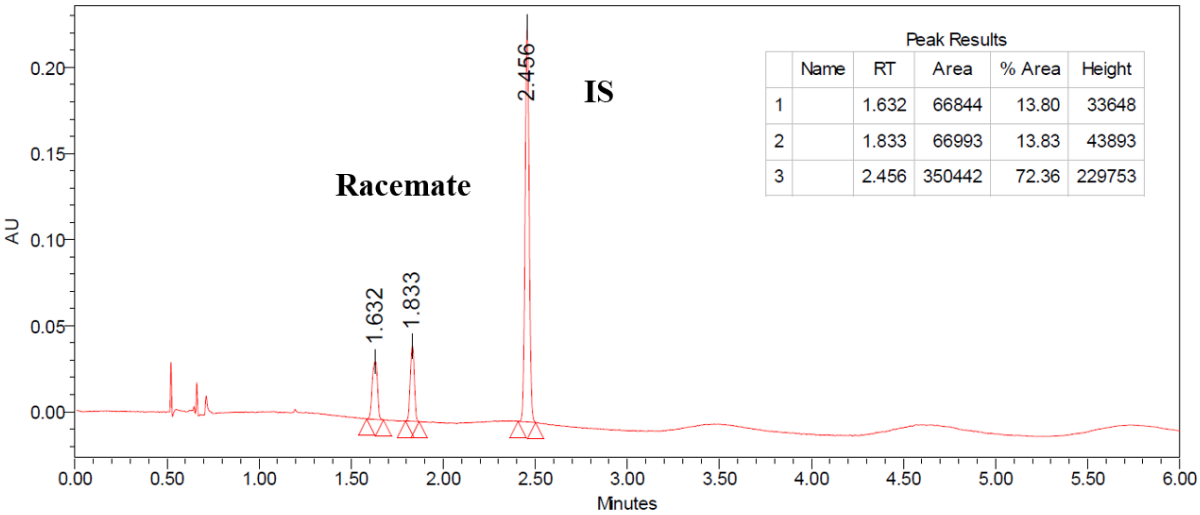

	Name	RT	Area	% Area	Height
1		1.632	66844	13.80	33648
2		1.833	66993	13.83	43893
3		2.456	350442	72.36	229753

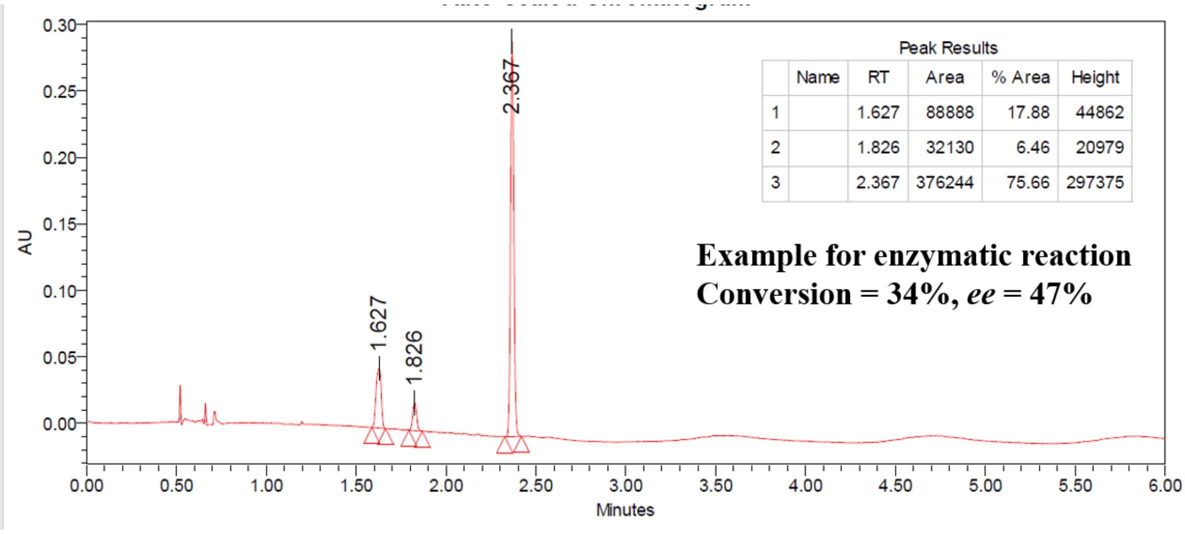

	Name	RT	Area	% Area	Height
1		1.627	88888	17.88	44862
2		1.826	32130	6.46	20979
3		2.367	376244	75.66	297375

1c

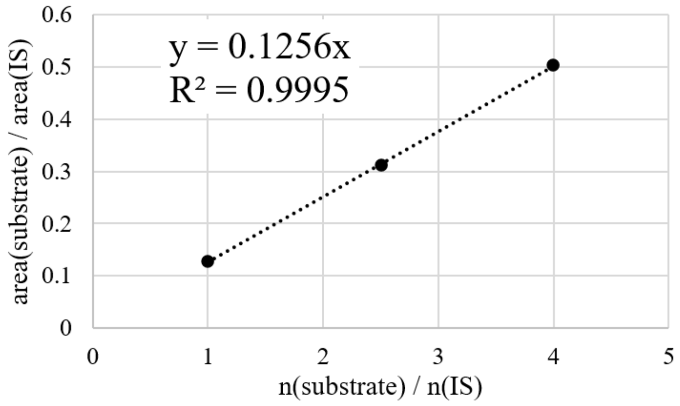

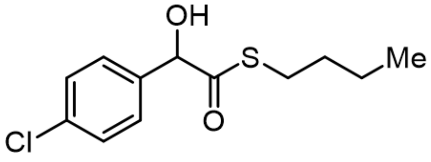

S-butyl 2-(4-chlorophenyl)-2-hydroxyethanethioate
(*R*,*R*)Whelk-01, method 1, 245 nm
enantiomer A = 1.87 min,
enantiomer B = 2.17 min

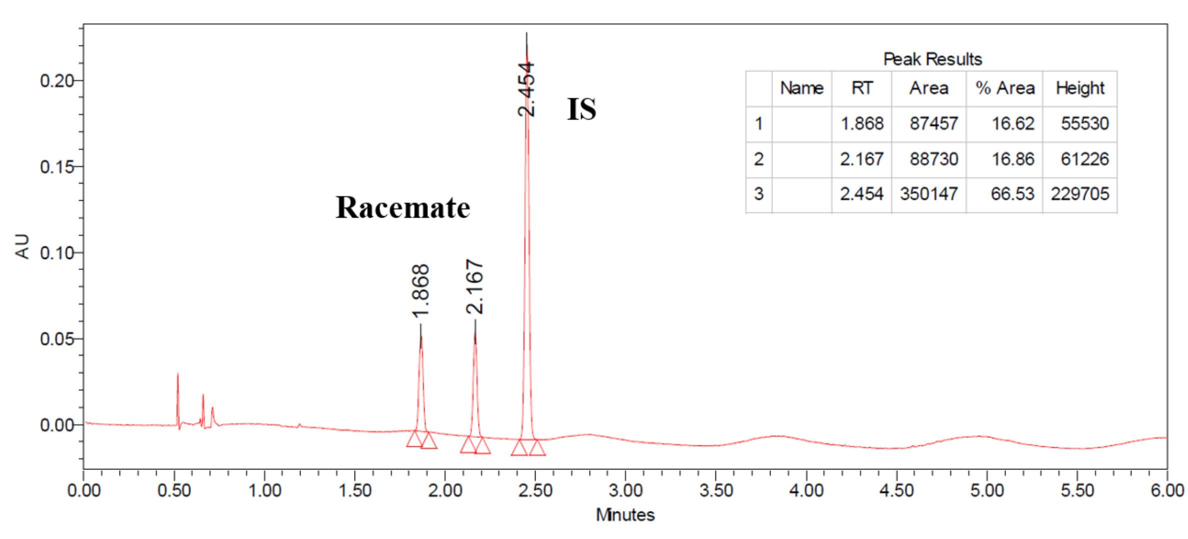

Peak Results

	Name	RT	Area	% Area	Height
1		1.868	87457	16.62	55530
2		2.167	88730	16.86	61226
3		2.454	350147	66.53	229705

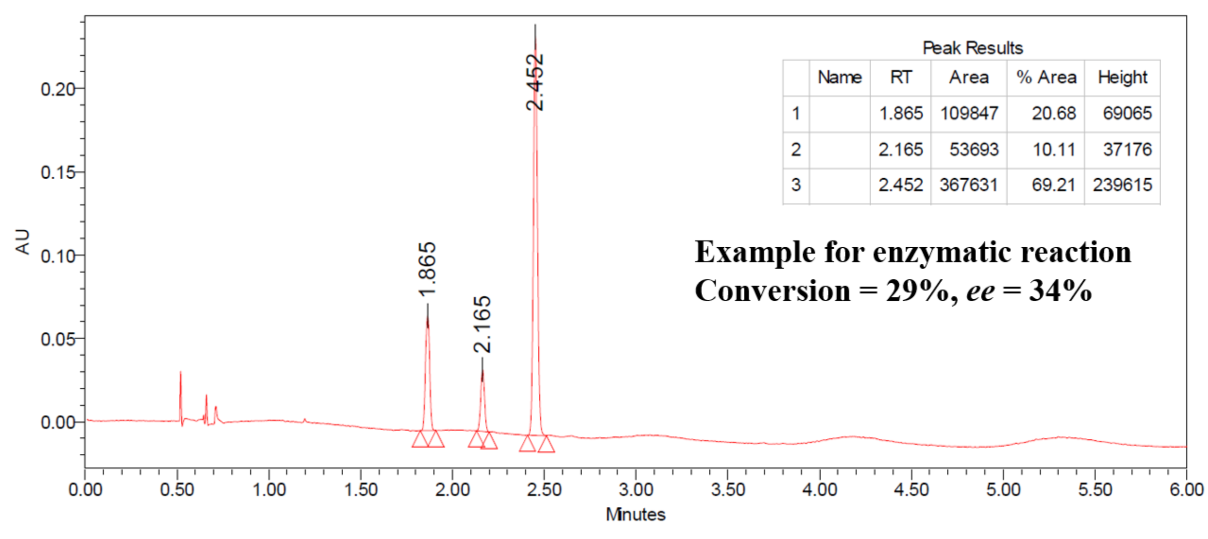

Peak Results

	Name	RT	Area	% Area	Height
1		1.865	109847	20.68	69065
2		2.165	53693	10.11	37176
3		2.452	367631	69.21	239615

Example for enzymatic reaction
Conversion = 29%, *ee* = 34%

1d

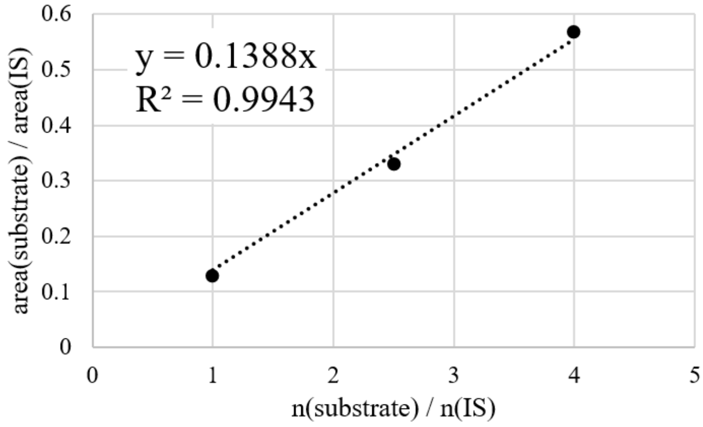

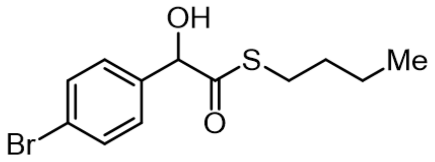

S-butyl 2-(4-bromophenyl)-2-hydroxyethanethioate
(*R*,*R*)Whelk-01, method 1, 245 nm
enantiomer A = 2.00 min, enantiomer B = 2.35 min

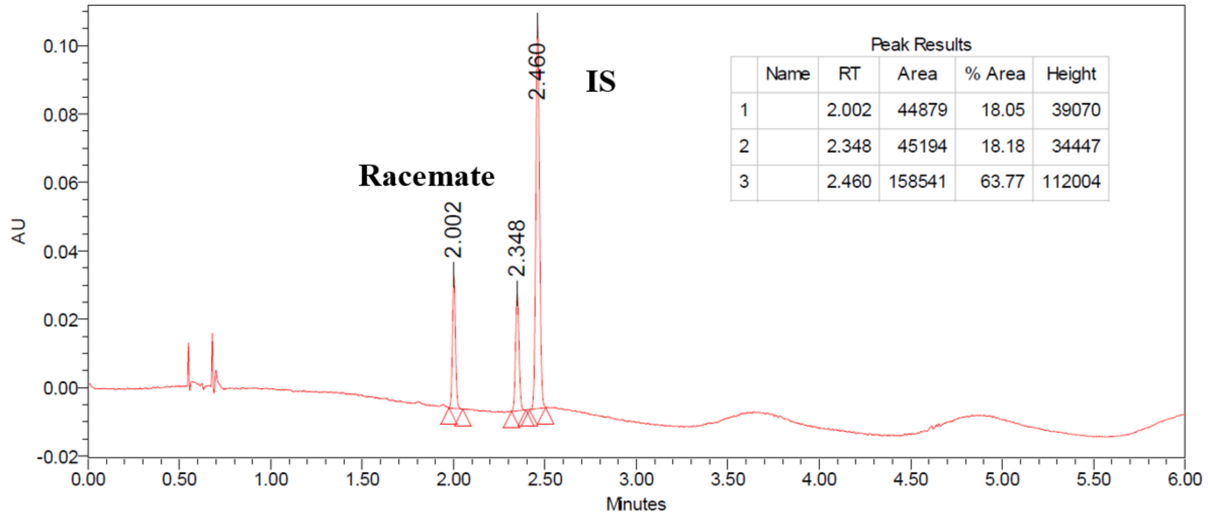

Peak Results

	Name	RT	Area	% Area	Height
1		2.002	44879	18.05	39070
2		2.348	45194	18.18	34447
3		2.460	158541	63.77	112004

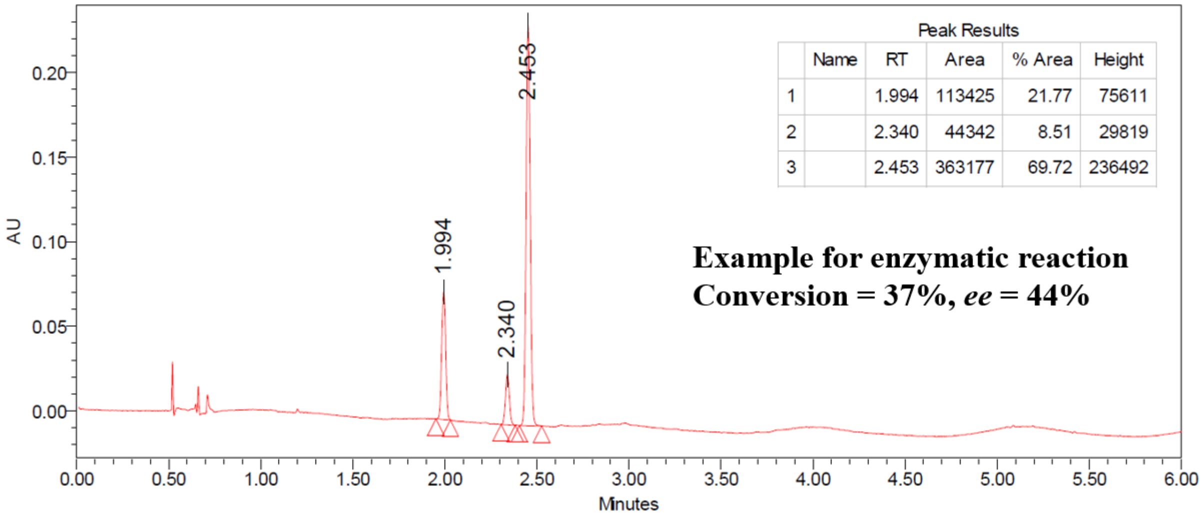

Peak Results

	Name	RT	Area	% Area	Height
1		1.994	113425	21.77	75611
2		2.340	44342	8.51	29819
3		2.453	363177	69.72	236492

1e

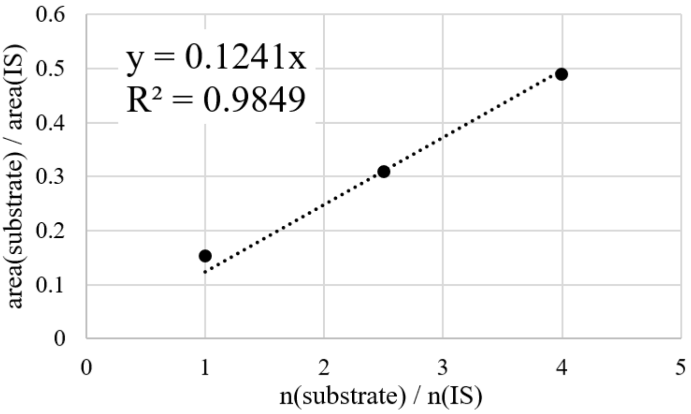

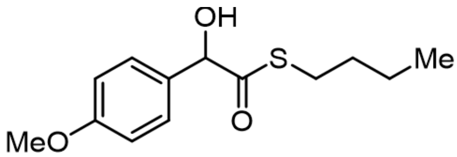

S-butyl 2-hydroxy-2-(4-methoxyphenyl)ethanethioate
(*R*,*R*)Whelk-01, method 2, 245 nm
enantiomer A = 3.12 min,
enantiomer B = 3.99 min

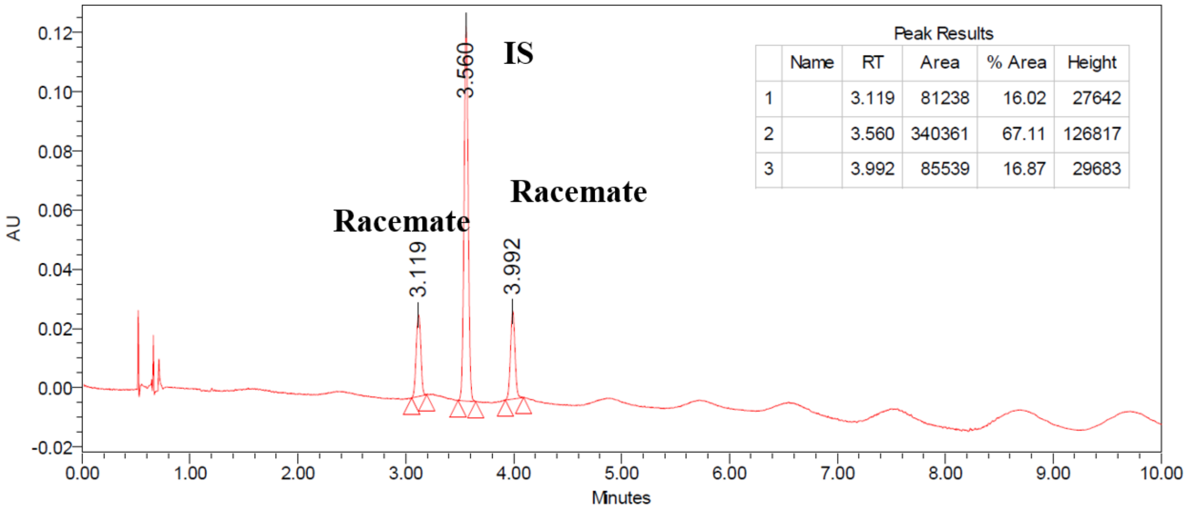

Peak Results

	Name	RT	Area	% Area	Height
1		3.119	81238	16.02	27642
2		3.560	340361	67.11	126817
3		3.992	85539	16.87	29683

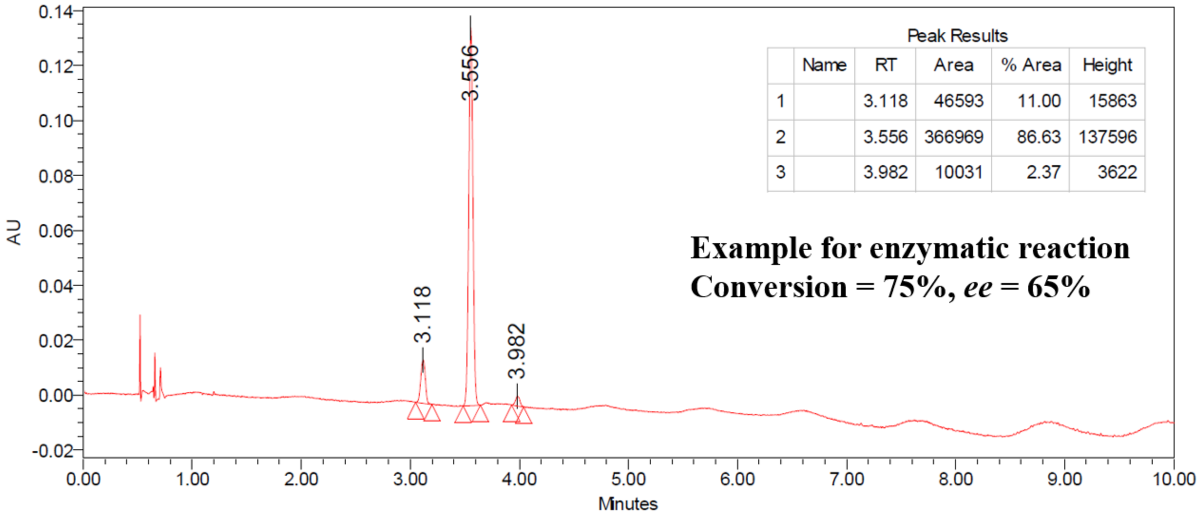

Peak Results

	Name	RT	Area	% Area	Height
1		3.118	46593	11.00	15863
2		3.556	366969	86.63	137596
3		3.982	10031	2.37	3622

Example for enzymatic reaction
Conversion = 75%, *ee* = 65%

1f

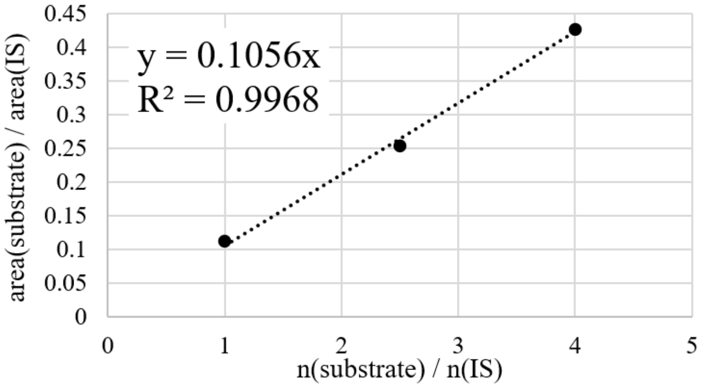

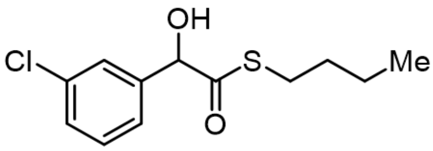

S-butyl 2-(3-chlorophenyl)-2-hydroxyethanethioate
(*R*,*R*)Whelk-01, method 1, 245 nm
enantiomer A = 1.88 min,
enantiomer B = 2.09 min

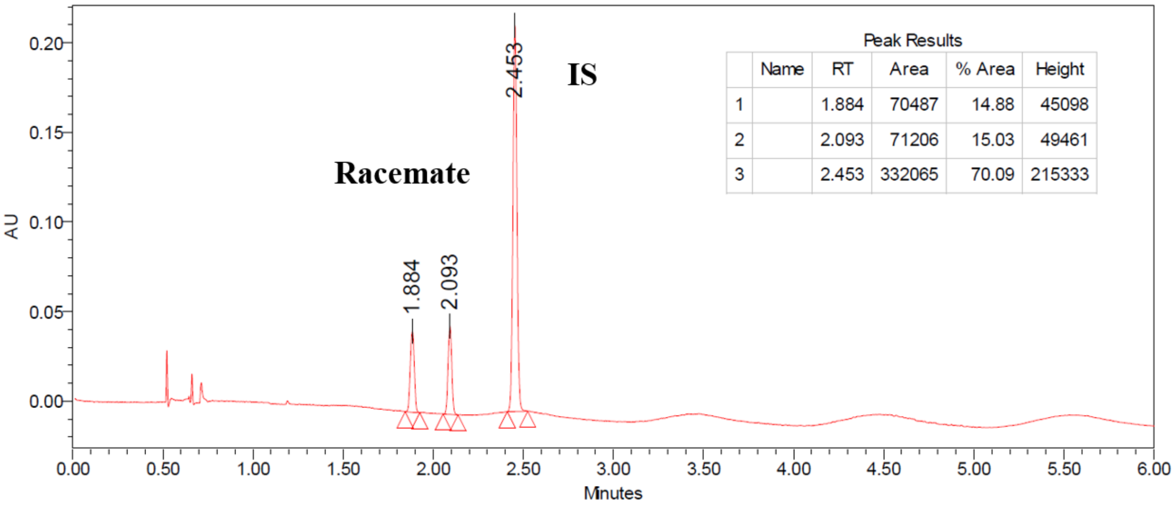

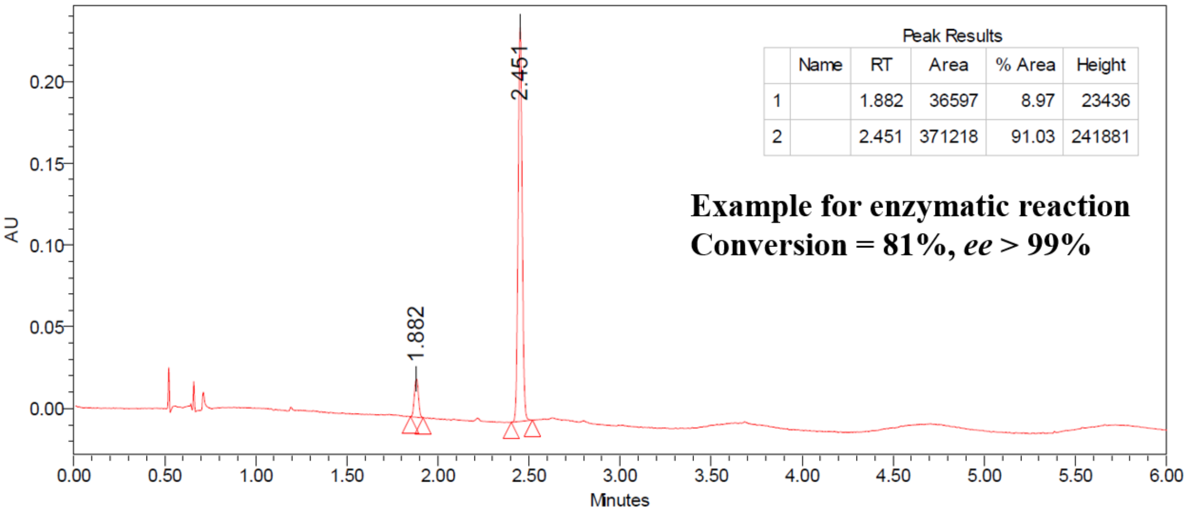

1g

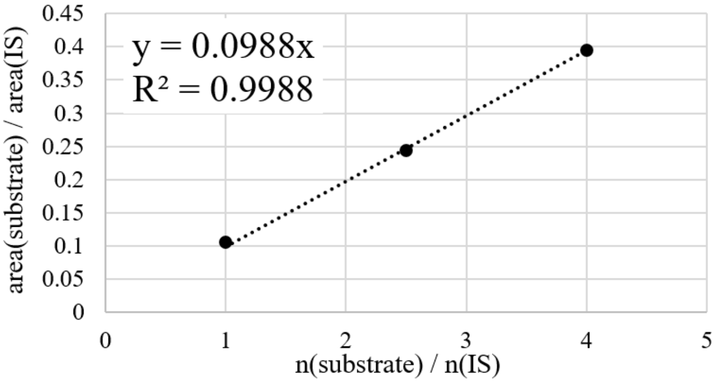

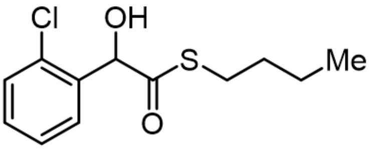

S-butyl 2-(2-chlorophenyl)-2-hydroxyethanethioate
(*R*,*R*)Whelk-01, method 1, 245 nm
enantiomer A = 1.94 min,
enantiomer B = 2.02 min

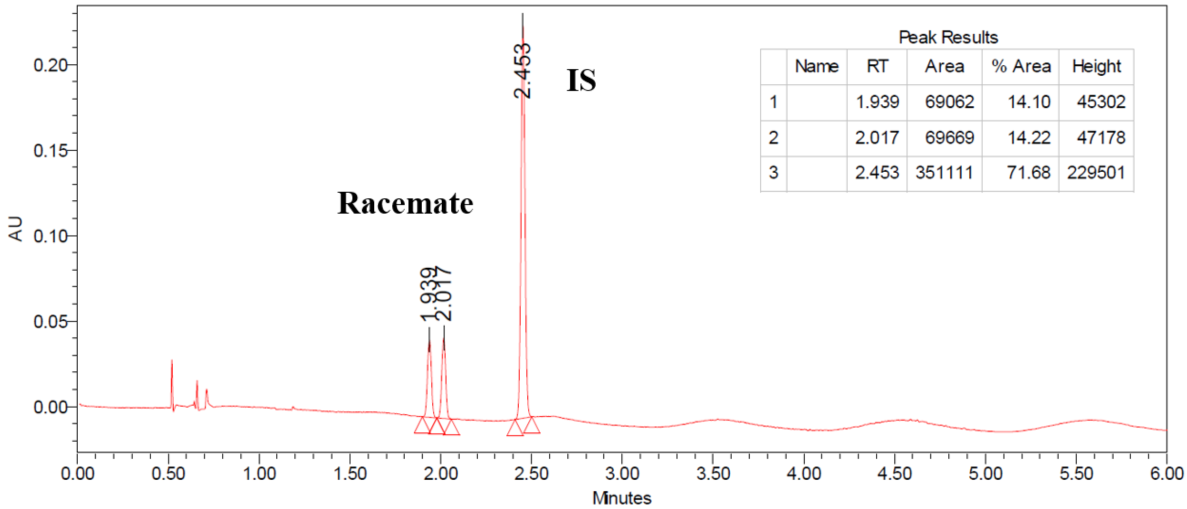

Peak Results

	Name	RT	Area	% Area	Height
1		1.939	69062	14.10	45302
2		2.017	69669	14.22	47178
3		2.453	351111	71.68	229501

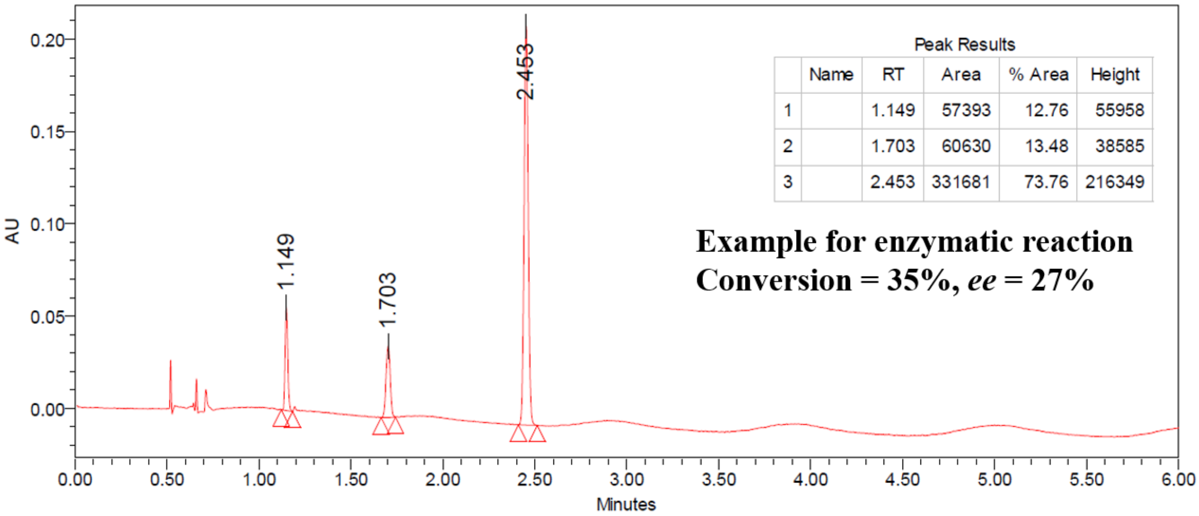

Peak Results

	Name	RT	Area	% Area	Height
1		1.149	57393	12.76	55958
2		1.703	60630	13.48	38585
3		2.453	331681	73.76	216349

Example for enzymatic reaction
Conversion = 35%, *ee* = 27%

1h

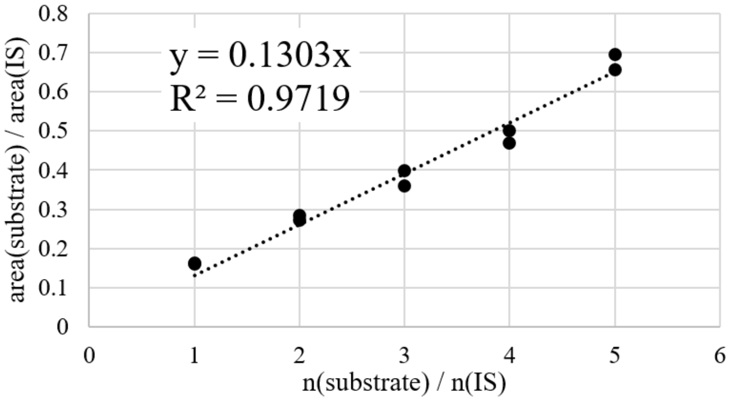

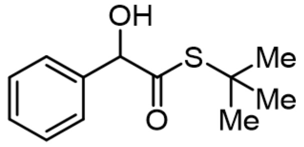

S-(tert-butyl) 2-hydroxy-2-phenylethanethioate
Chiralpak AD-H, heptane:*i*PrOH = 90:10, flow rate 1.0 ml/min, 245 nm
enantiomer A = 6.88 min,
enantiomer B = 7.27 min

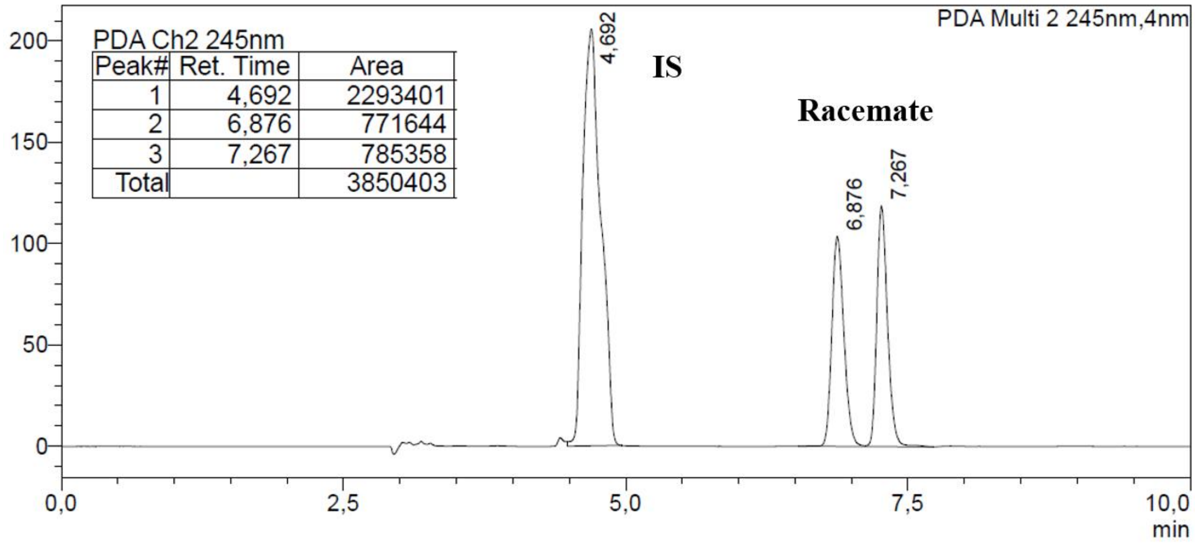

PDA Ch2 245nm

Peak#	Ret. Time	Area
1	4,692	2293401
2	6,876	771644
3	7,267	785358
Total		3850403

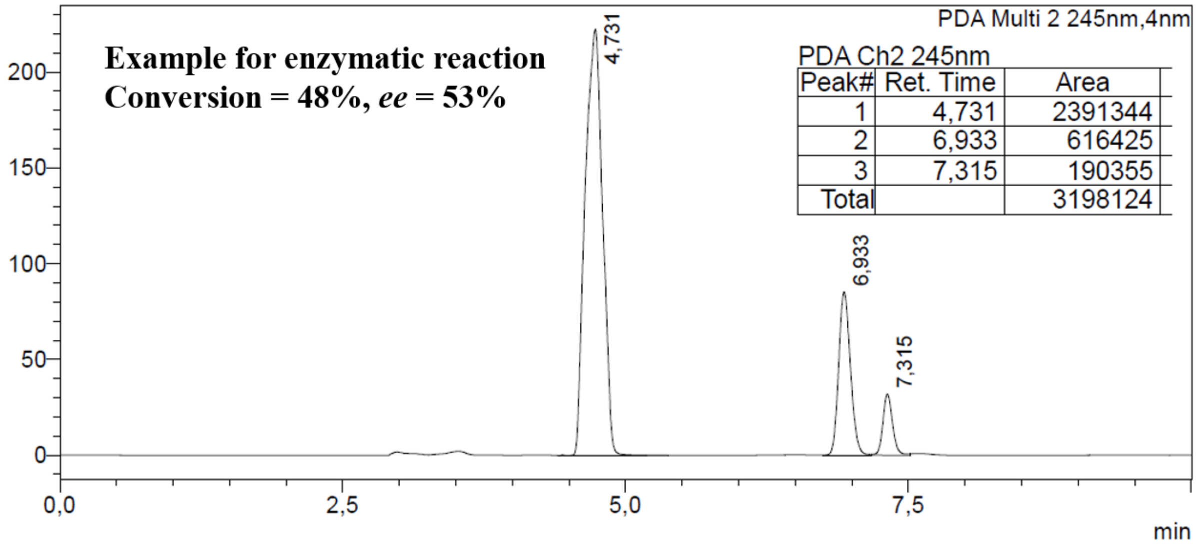

PDA Ch2 245nm

Peak#	Ret. Time	Area
1	4,731	2391344
2	6,933	616425
3	7,315	190355
Total		3198124

1i

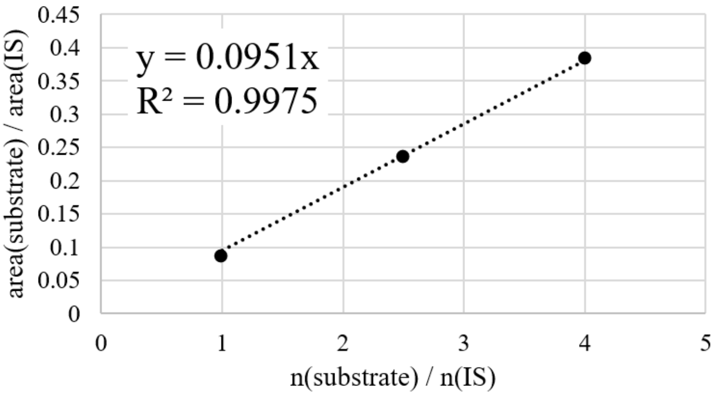

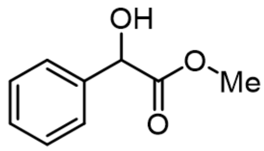

methyl 2-hydroxy-2-phenylacetate
Chiralpak AD-H, heptane:*i*PrOH = 93:7, flow rate 1.0 ml/min, 220 nm
enantiomer A = 10.3 min, enantiomer B = 11.0 min

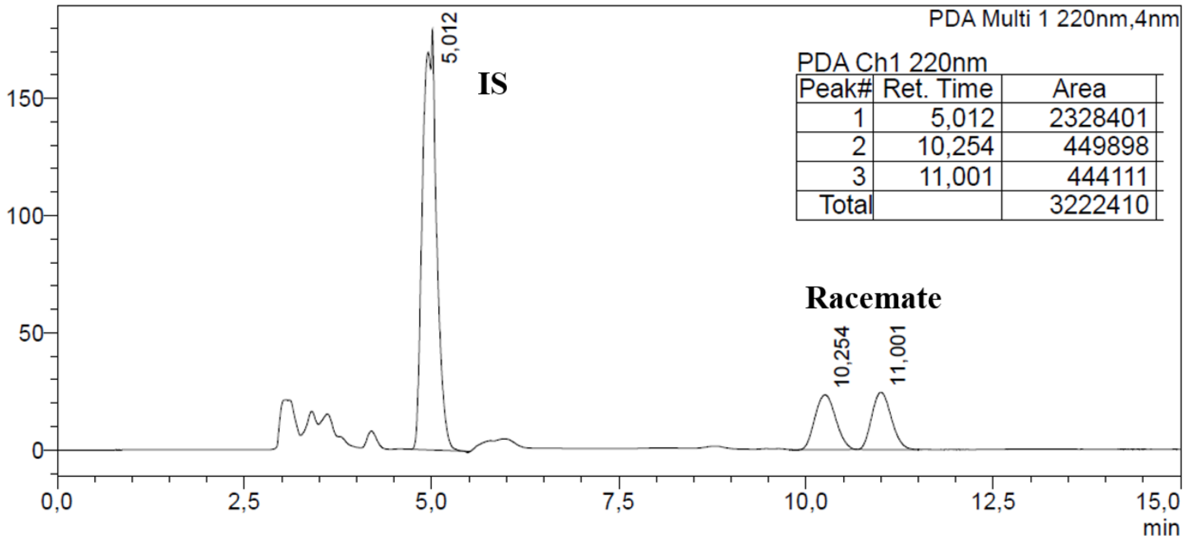

Peak#	Ret. Time	Area
1	5,012	2328401
2	10,254	449898
3	11,001	444111
Total		3222410

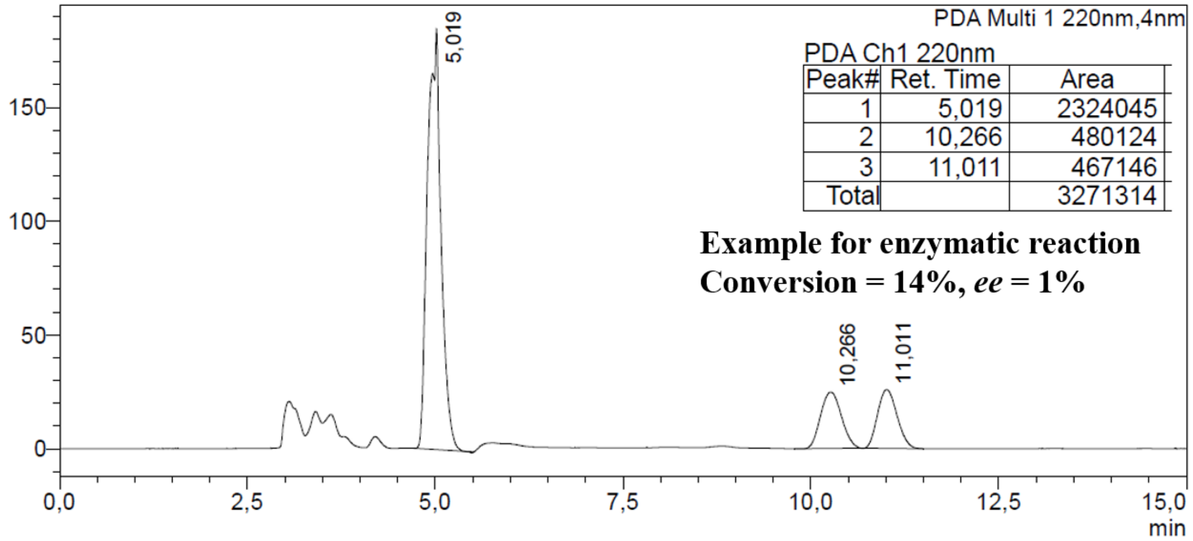

Peak#	Ret. Time	Area
1	5,019	2324045
2	10,266	480124
3	11,011	467146
Total		3271314

1j

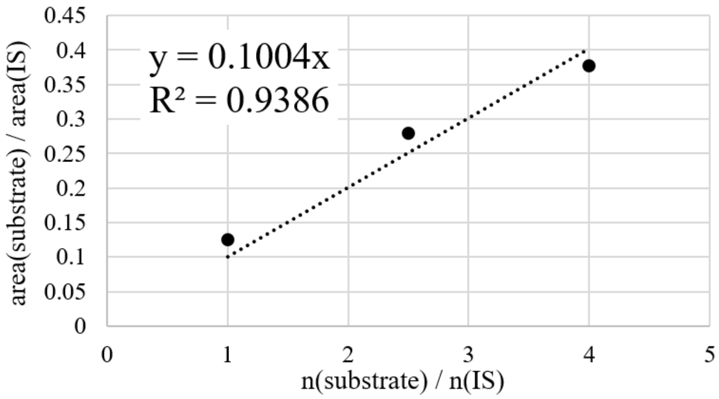

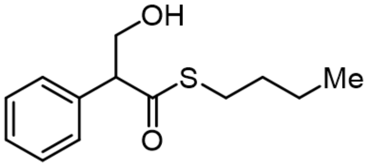

S-butyl 3-hydroxy-2-phenylpropanethioate
(*R*,*R*)Whelk-01, method 1, 245 nm
enantiomer A = 1.86 min, enantiomer B = 1.95 min

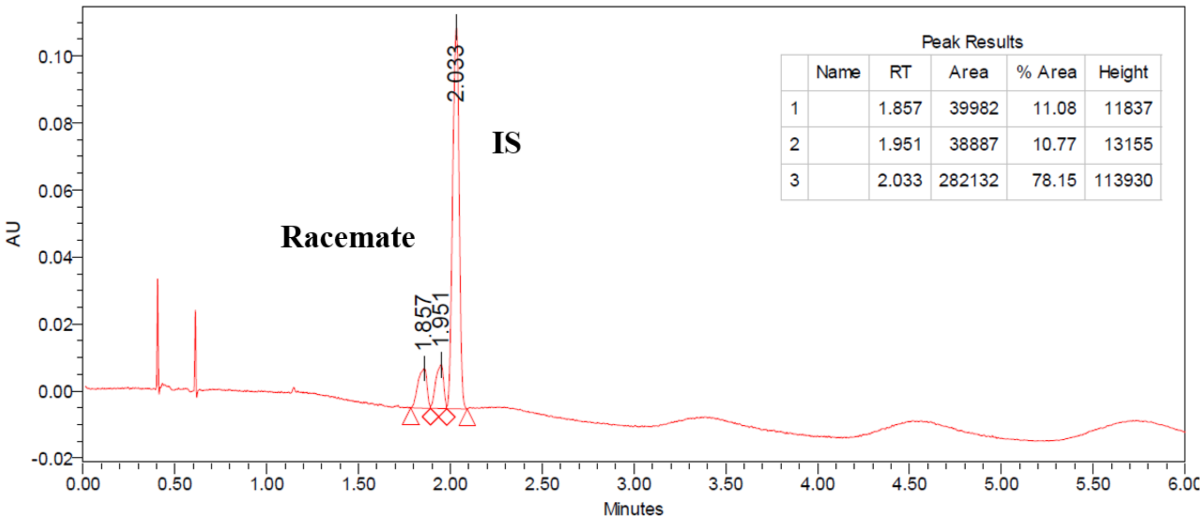

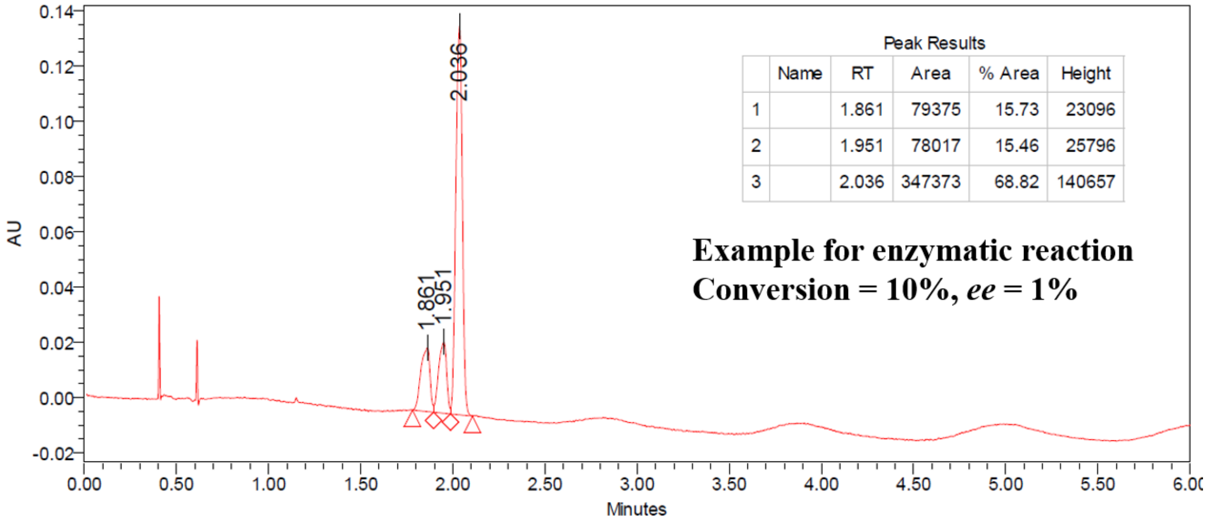

1k

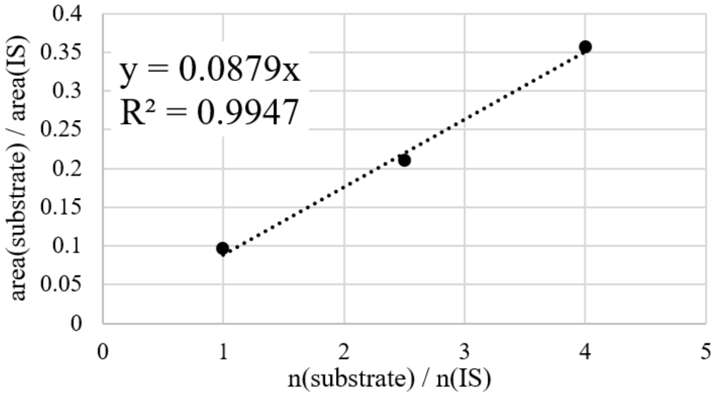

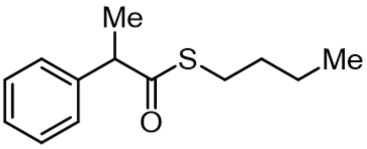

S-butyl 2-phenylpropanethioate
(*R*,*R*)Whelk-01, method 1, 245 nm
enantiomer A = 1.15 min,
enantiomer B = 1.70 min

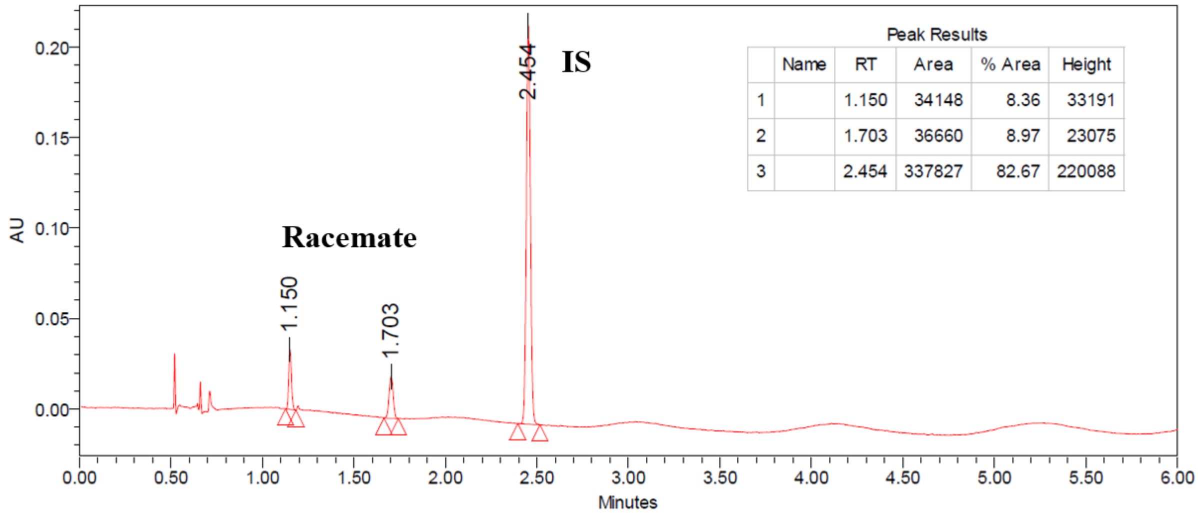

Peak Results

	Name	RT	Area	% Area	Height
1		1.150	34148	8.36	33191
2		1.703	36660	8.97	23075
3		2.454	337827	82.67	220088

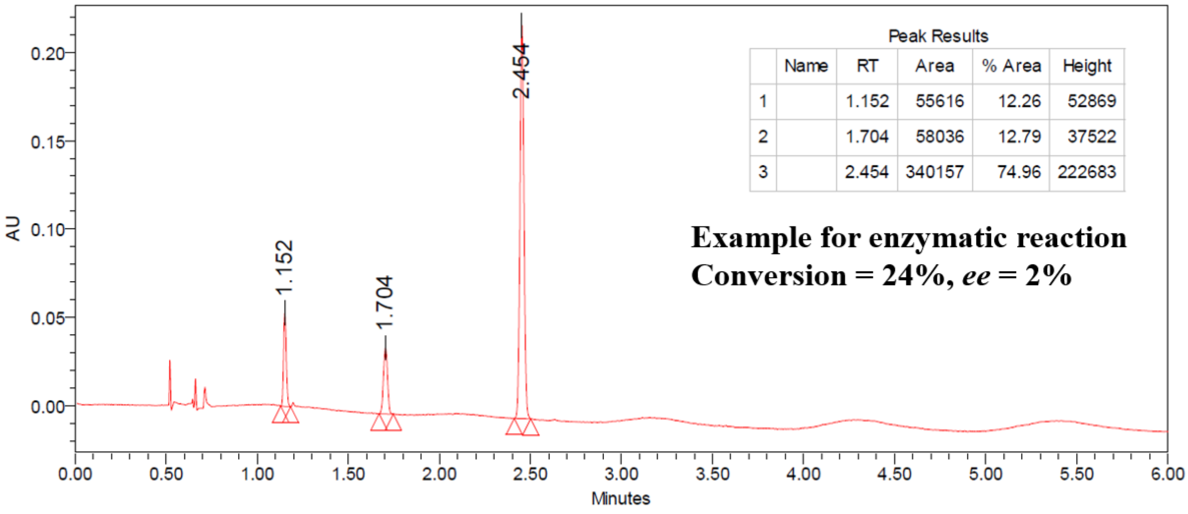

Peak Results

	Name	RT	Area	% Area	Height
1		1.152	55616	12.26	52869
2		1.704	58036	12.79	37522
3		2.454	340157	74.96	222683

Example for enzymatic reaction
Conversion = 24%, *ee* = 2%

2a

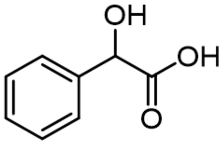

2-hydroxy-2-phenylacetic acid
3126 (D)-penicillamine column (250 × 4 mm, *Chirex*), 0.5 mM $CuSO_4$/*i*PrOH (9/1), 1.0 mL/min, 50 °C, 210 nm
Enantiomer (*S*) = 31.91 min,
enantiomer (*R*) = 34.95 min

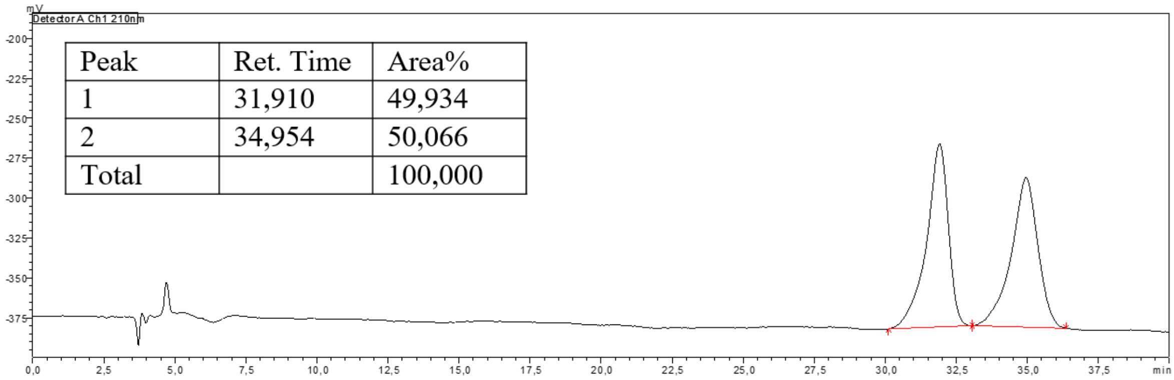

Peak	Ret. Time	Area%
1	31,910	49,934
2	34,954	50,066
Total		100,000

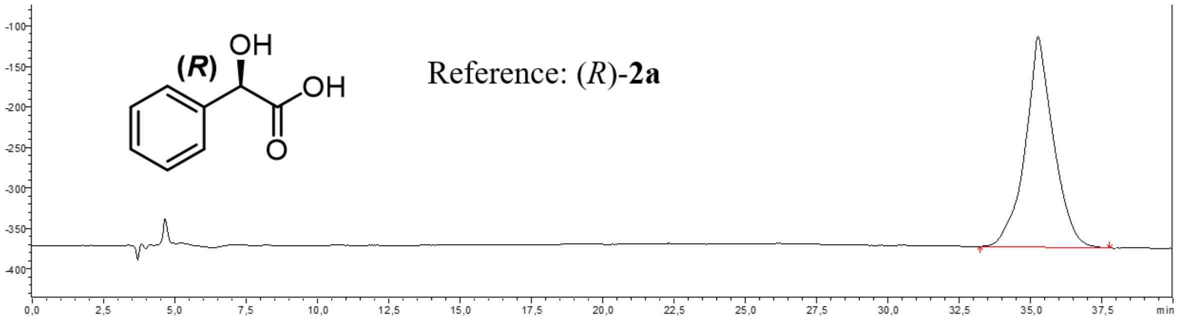

Reference: (*R*)-**2a**

Peak	Ret. Time	Area%
1	31,798	11,740
2	35,544	88,260
Total		100,000

Example for enzymatic reaction
***ee* = 77%**

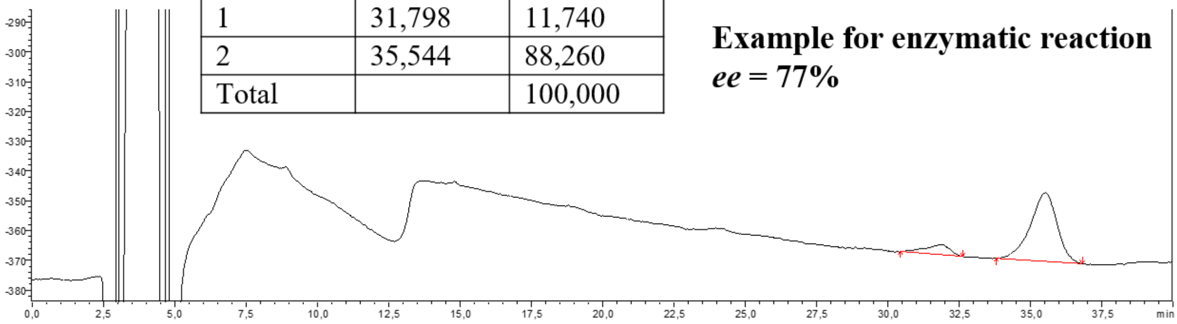

SI.16 NMR spectra

^{1}H NMR of **1a**

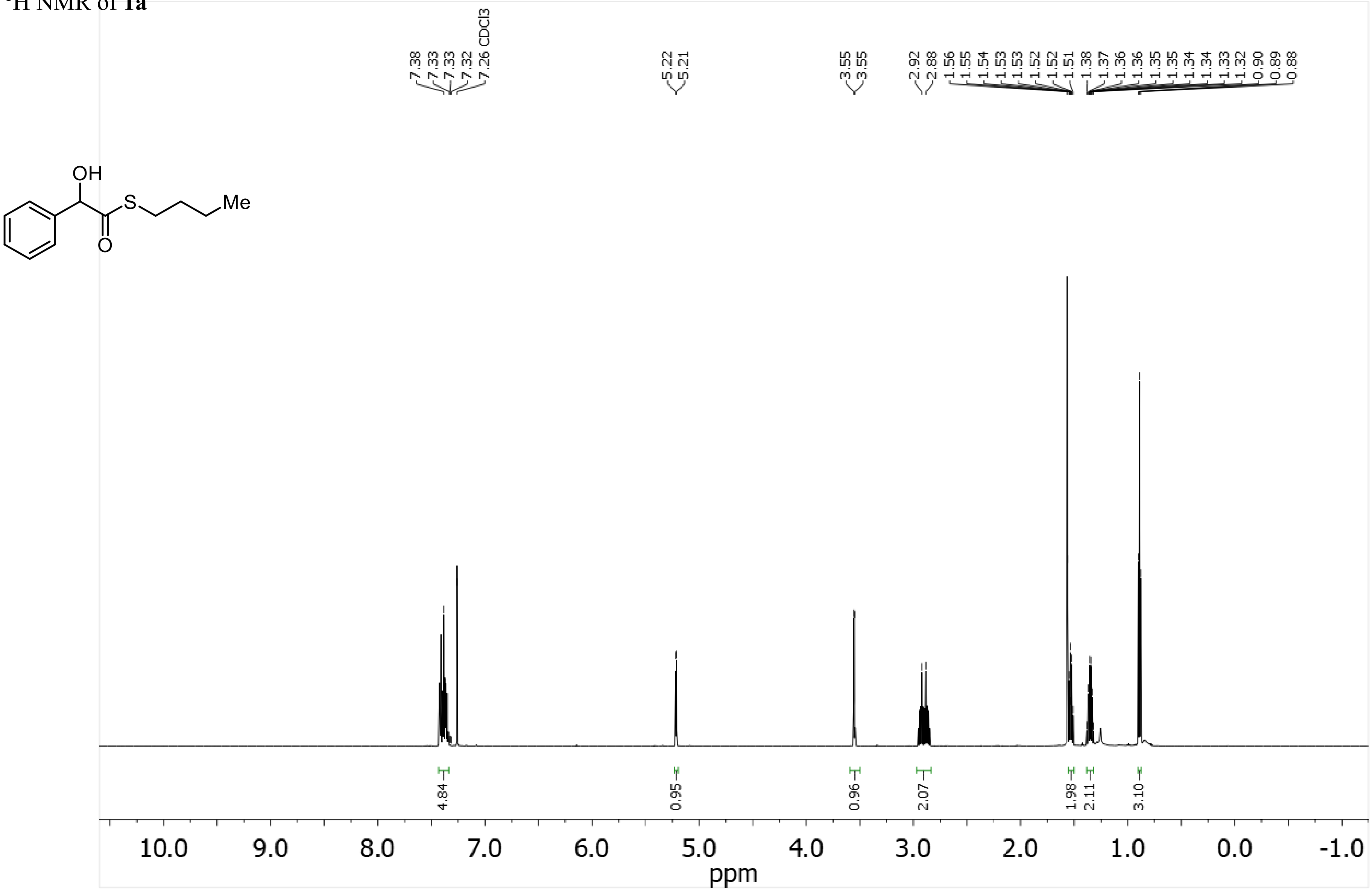

^{1}H NMR of **1b**

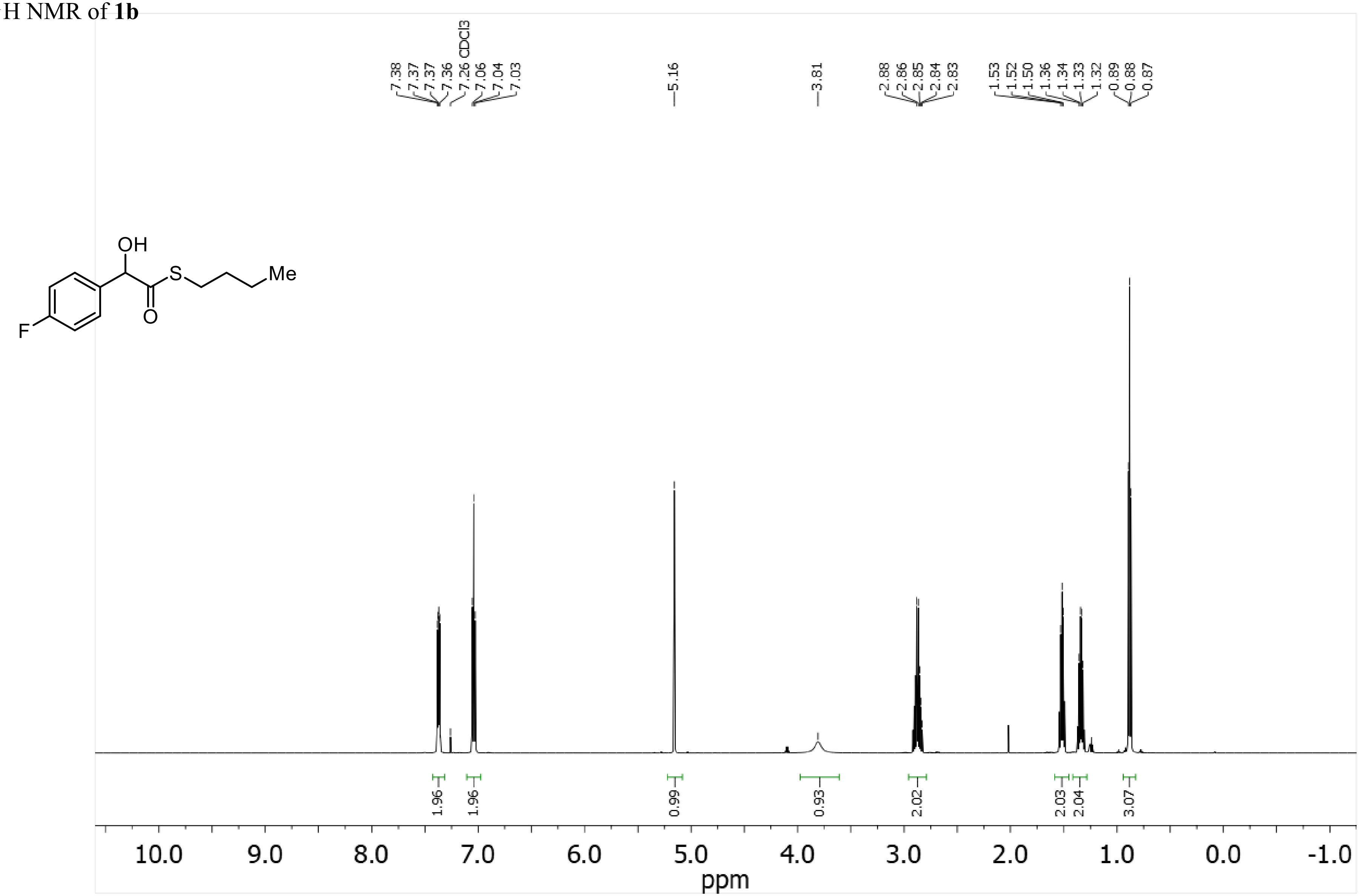

OH
S
Me
F
O
7.38
7.37
7.37
7.36
7.26 CDCl3
7.06
7.04
7.03
5.16
3.81
2.88
2.86
2.85
2.84
2.83
1.53
1.52
1.50
1.36
1.34
1.33
1.32
0.89
0.88
0.87
1.96
1.96
0.99
0.93
2.02
2.03
2.04
3.07
10.0
9.0
8.0
7.0
6.0
5.0
4.0
3.0
2.0
1.0
0.0
-1.0
ppm

^{13}C NMR of **1b**

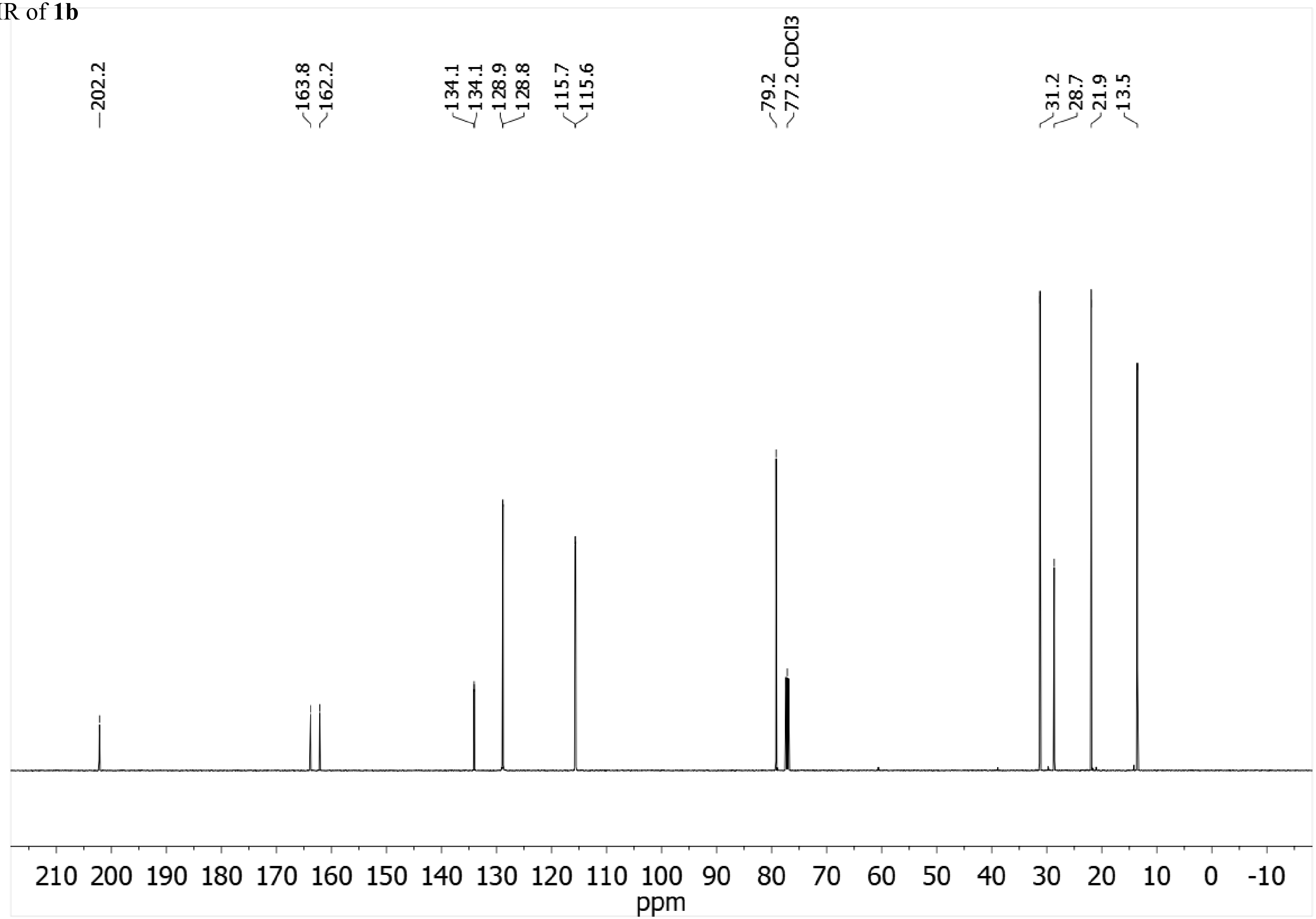

202.2
163.8
162.2
134.1
134.1
128.9
128.8
115.7
115.6
79.2
77.2 CDCl3
31.2
28.7
21.9
13.5
210 200 190 180 170 160 150 140 130 120 110 100 90 80 70 60 50 40 30 20 10 0 -10
ppm

^{1}H NMR of **1c**

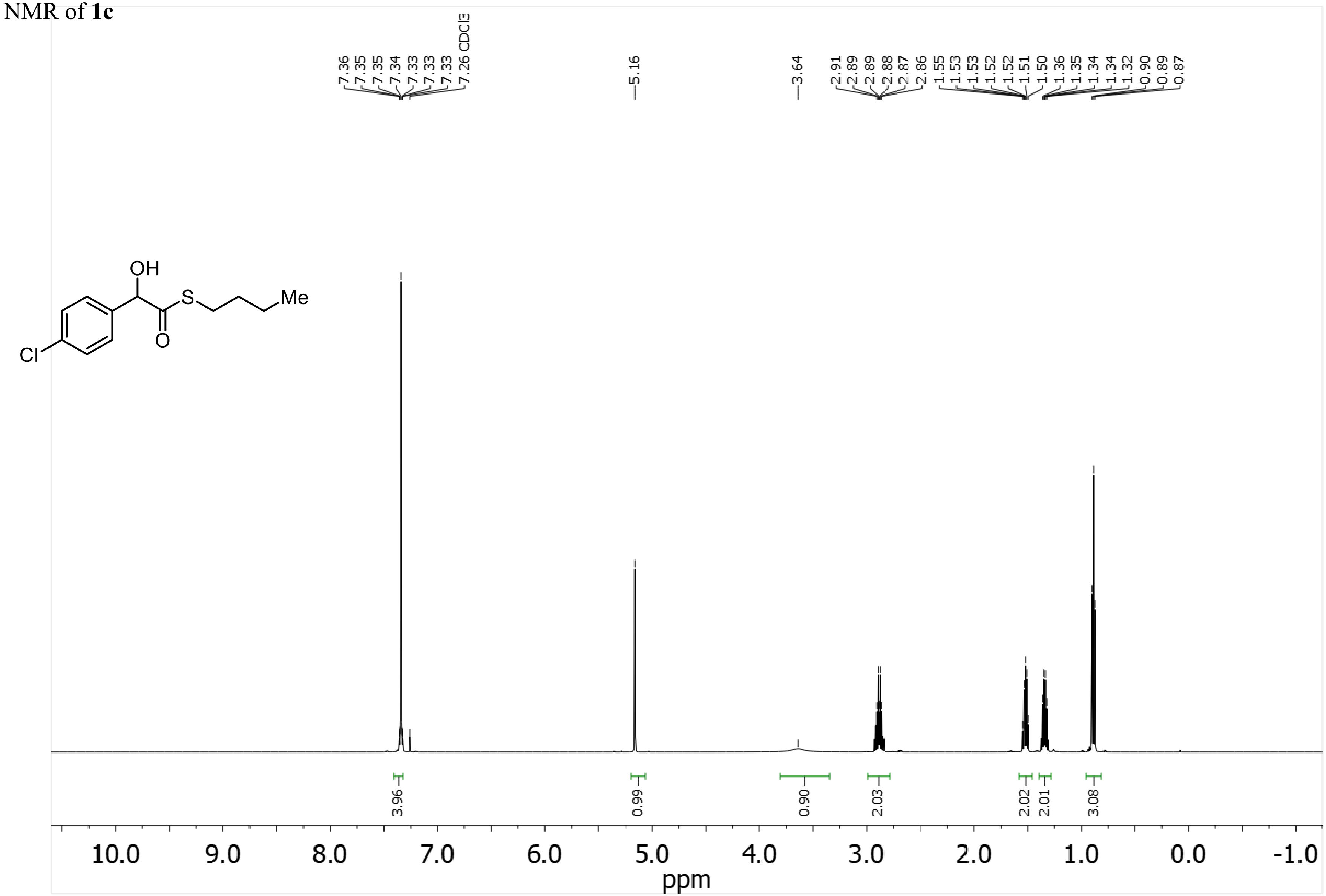
7.36
7.35
7.35
7.34
7.33
7.33
7.33
7.26 CDCl3
5.16
3.64
2.91
2.89
2.89
2.88
2.87
2.86
1.55
1.53
1.53
1.52
1.52
1.51
1.50
1.36
1.35
1.34
1.34
1.32
0.90
0.89
0.87
OH
S
Me
O
Cl
3.96
0.99
0.90
2.03
2.02
2.01
3.08
10.0
9.0
8.0
7.0
6.0
5.0
4.0
3.0
2.0
1.0
0.0
-1.0
ppm

^{13}C NMR of **1c**

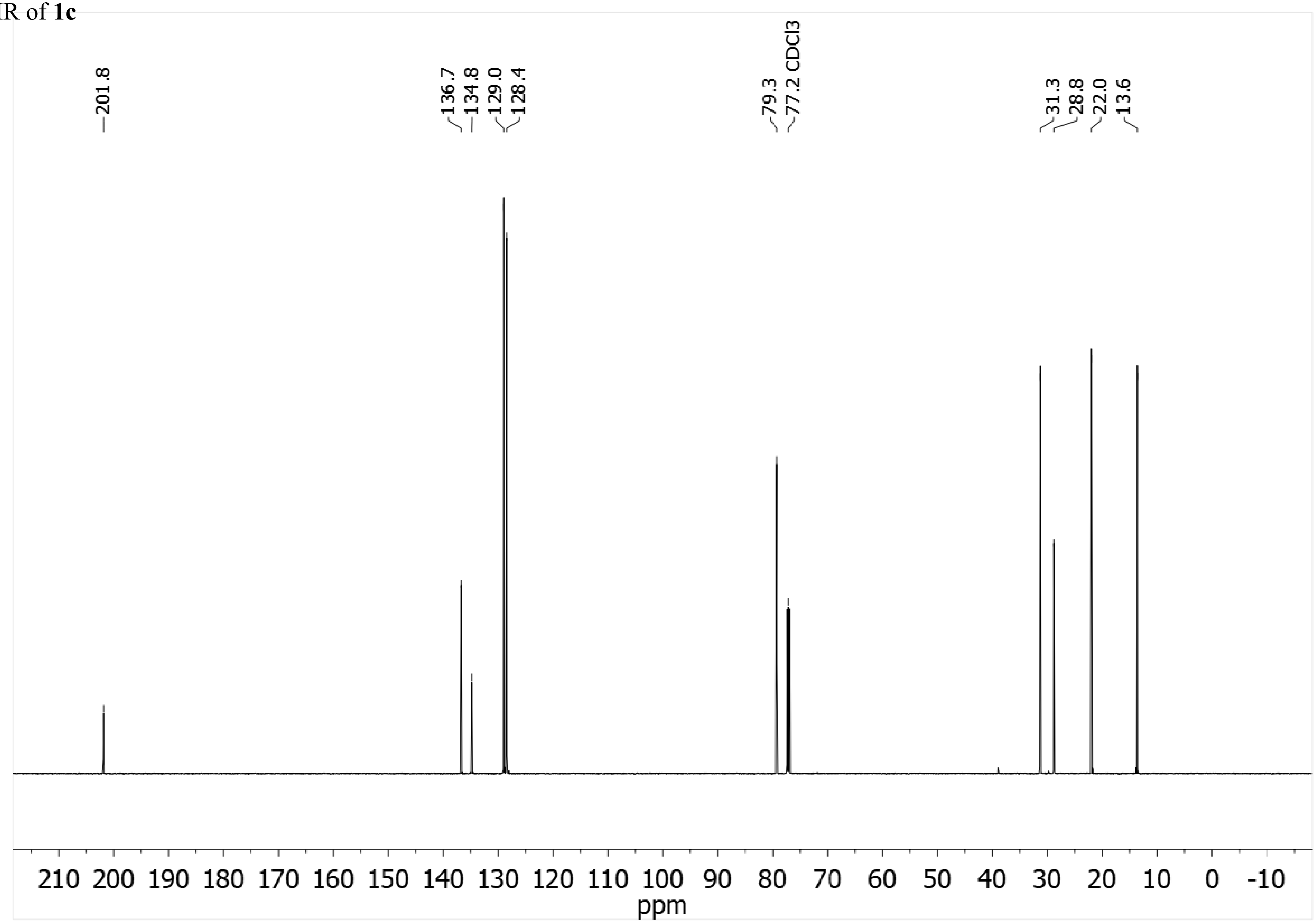

201.8
136.7
134.8
129.0
128.4
79.3
77.2 CDCl3
31.3
28.8
22.0
13.6
210 200 190 180 170 160 150 140 130 120 110 100 90 80 70 60 50 40 30 20 10 0 -10
ppm

^{1}H NMR of **1d**

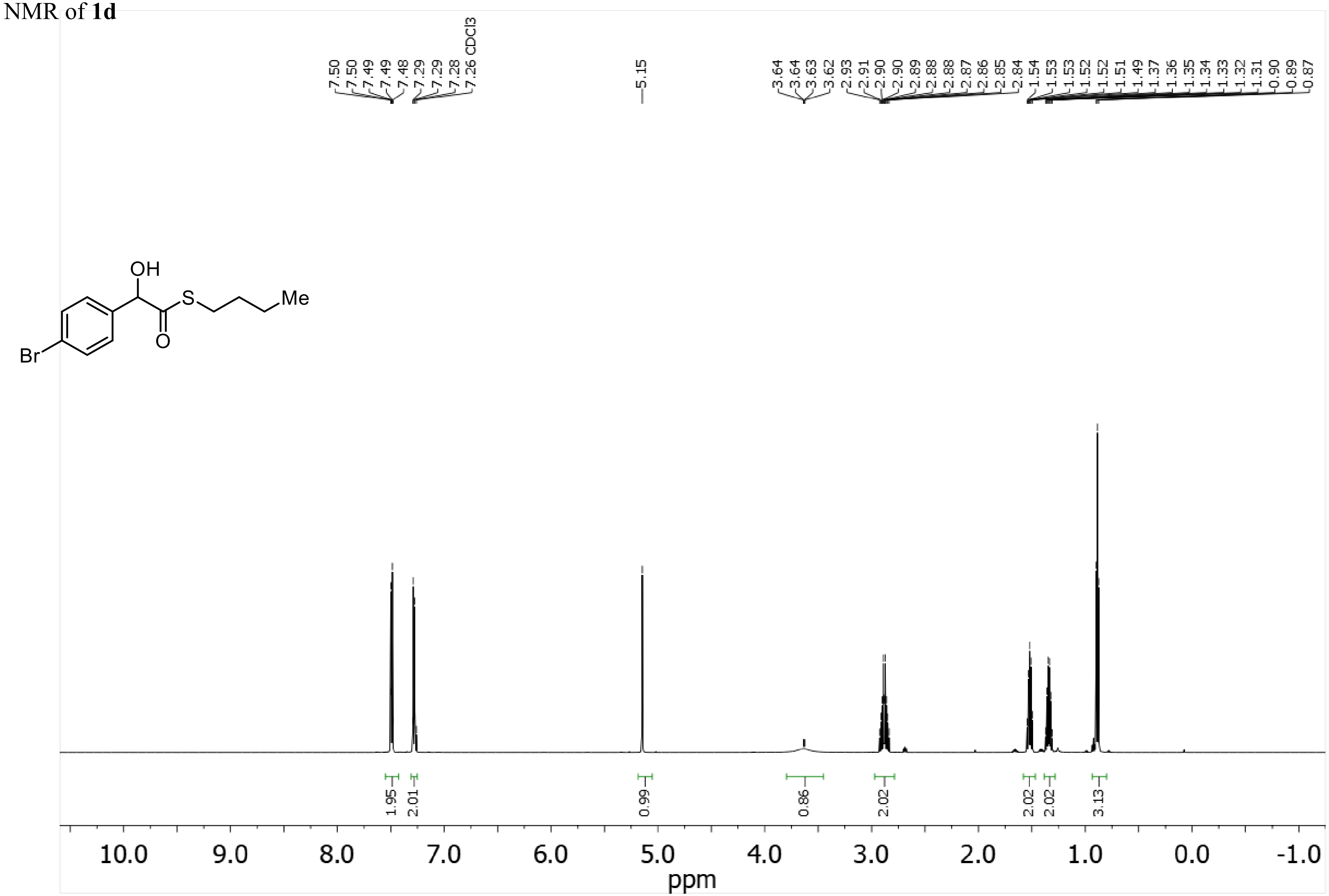

7.50
7.50
7.49
7.49
7.48
7.29
7.29
7.28
7.26 CDCl3
5.15
3.64
3.64
3.63
3.62
2.93
2.91
2.90
2.90
2.89
2.88
2.88
2.87
2.86
2.85
2.84
1.54
1.53
1.53
1.52
1.52
1.51
1.49
1.37
1.36
1.35
1.34
1.33
1.32
1.31
0.90
0.89
0.87
OH
S
Me
Br
O
1.95
2.01
0.99
0.86
2.02
2.02
2.02
3.13
10.0
9.0
8.0
7.0
6.0
5.0
4.0
3.0
2.0
1.0
0.0
-1.0
ppm

^{13}C NMR of **1d**

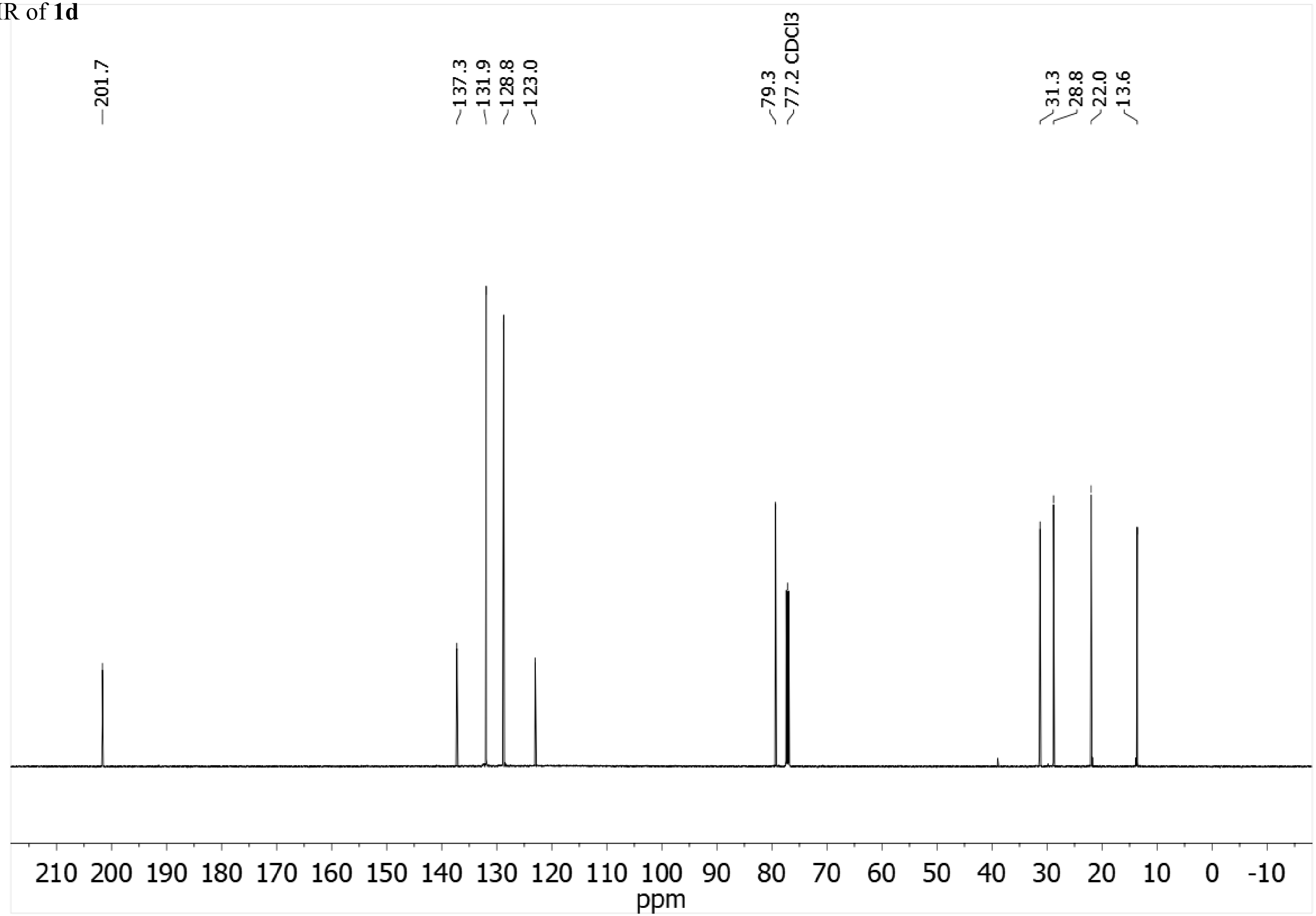
201.7
137.3
131.9
128.8
123.0
79.3
77.2 CDCl3
31.3
28.8
22.0
13.6
210 200 190 180 170 160 150 140 130 120 110 100 90 80 70 60 50 40 30 20 10 0 -10
ppm

^{1}H NMR of **1e**

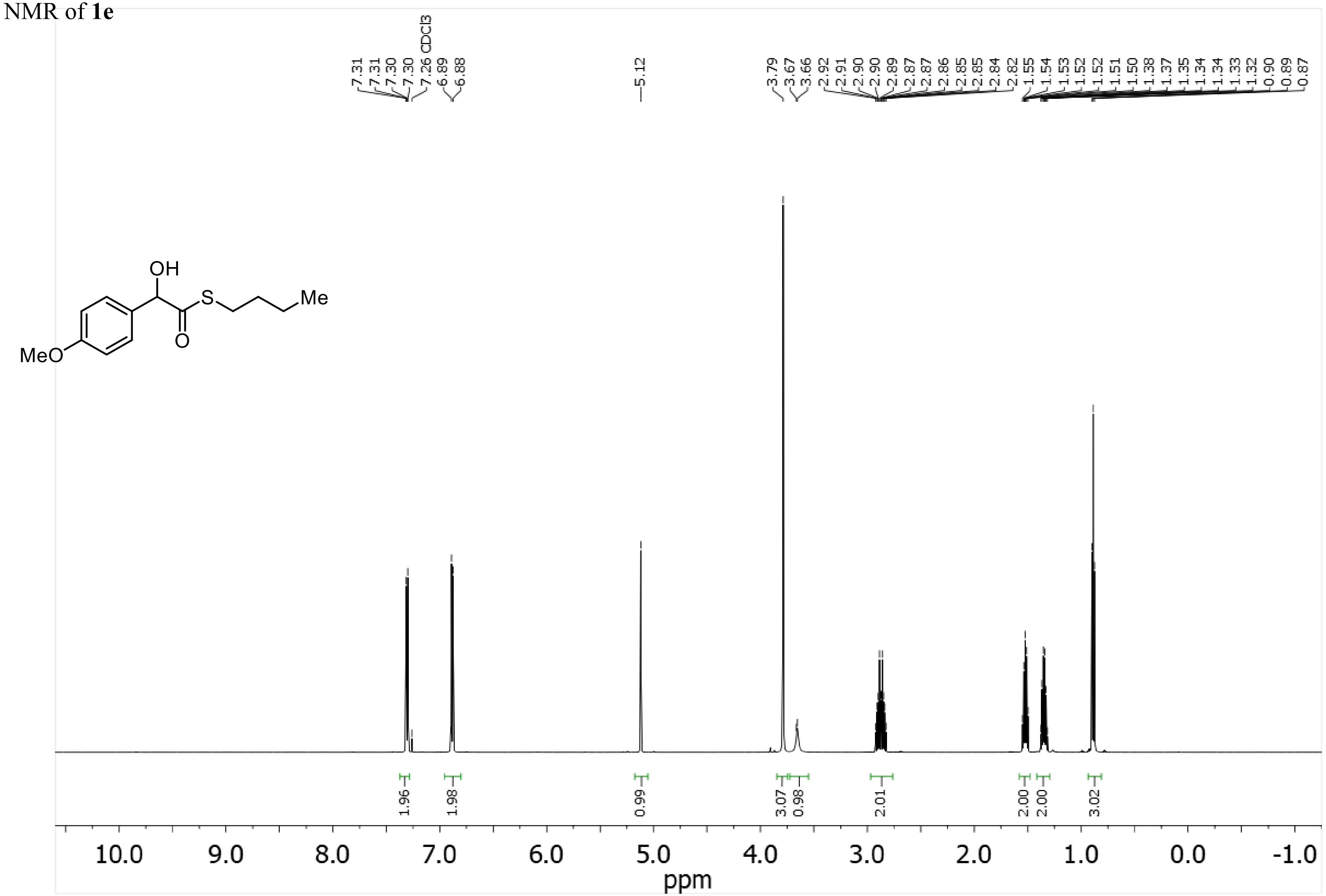

7.31
7.31
7.30
7.30
7.26 CDCl3
6.89
6.88
5.12
3.79
3.67
3.66
2.92
2.91
2.90
2.90
2.89
2.87
2.87
2.86
2.85
2.85
2.84
2.82
1.55
1.54
1.53
1.52
1.52
1.51
1.50
1.38
1.37
1.35
1.34
1.34
1.33
1.32
0.90
0.89
0.87
OH
S
Me
MeO
O
1.96
1.98
0.99
3.07
0.98
2.01
2.00
2.00
3.02
10.0
9.0
8.0
7.0
6.0
5.0
4.0
3.0
2.0
1.0
0.0
-1.0
ppm

^{13}C NMR of **1e**

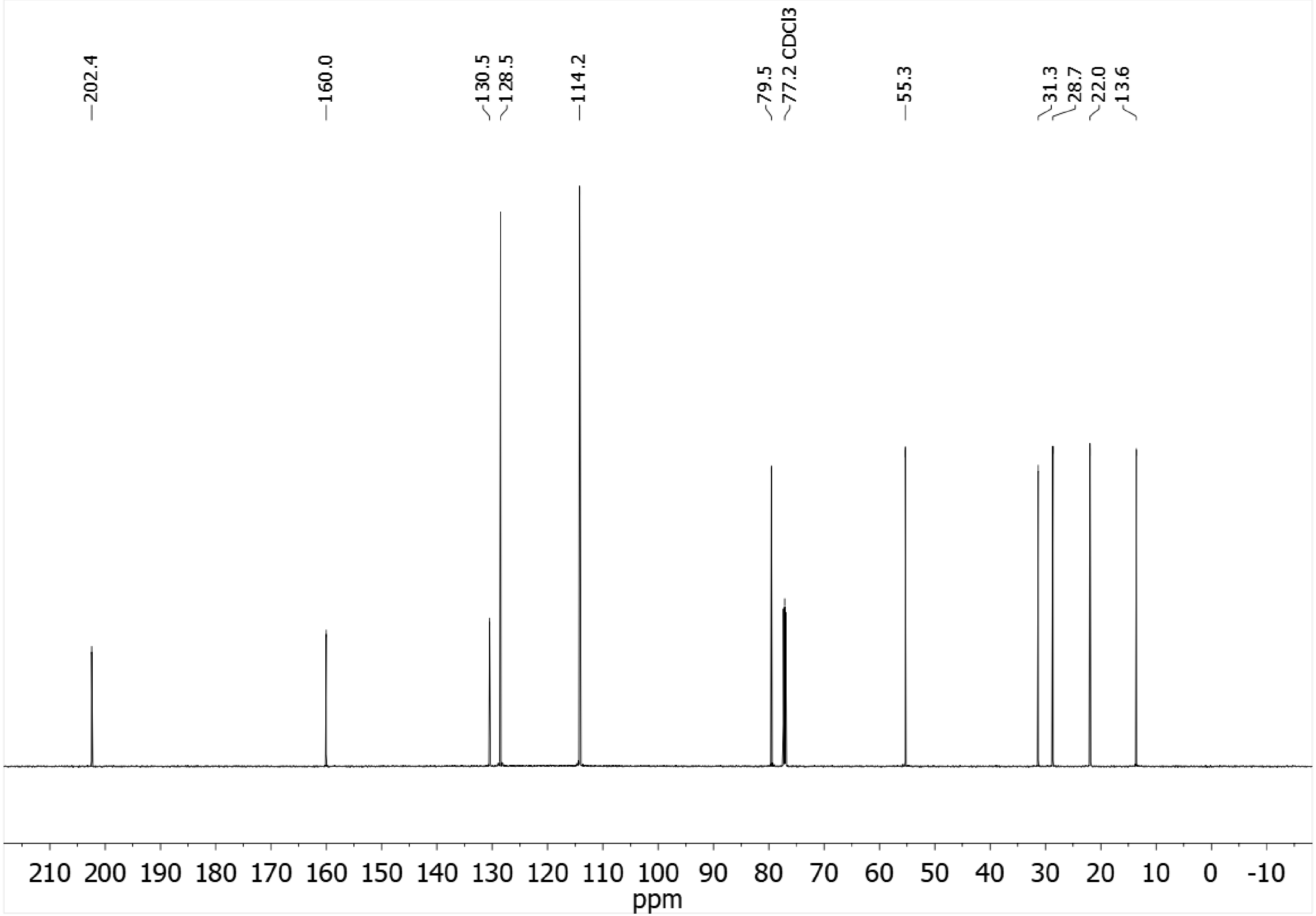

202.4
160.0
130.5
128.5
114.2
79.5
77.2 CDCl3
55.3
31.3
28.7
22.0
13.6
210 200 190 180 170 160 150 140 130 120 110 100 90 80 70 60 50 40 30 20 10 0 -10
ppm

^{1}H NMR of **1f**

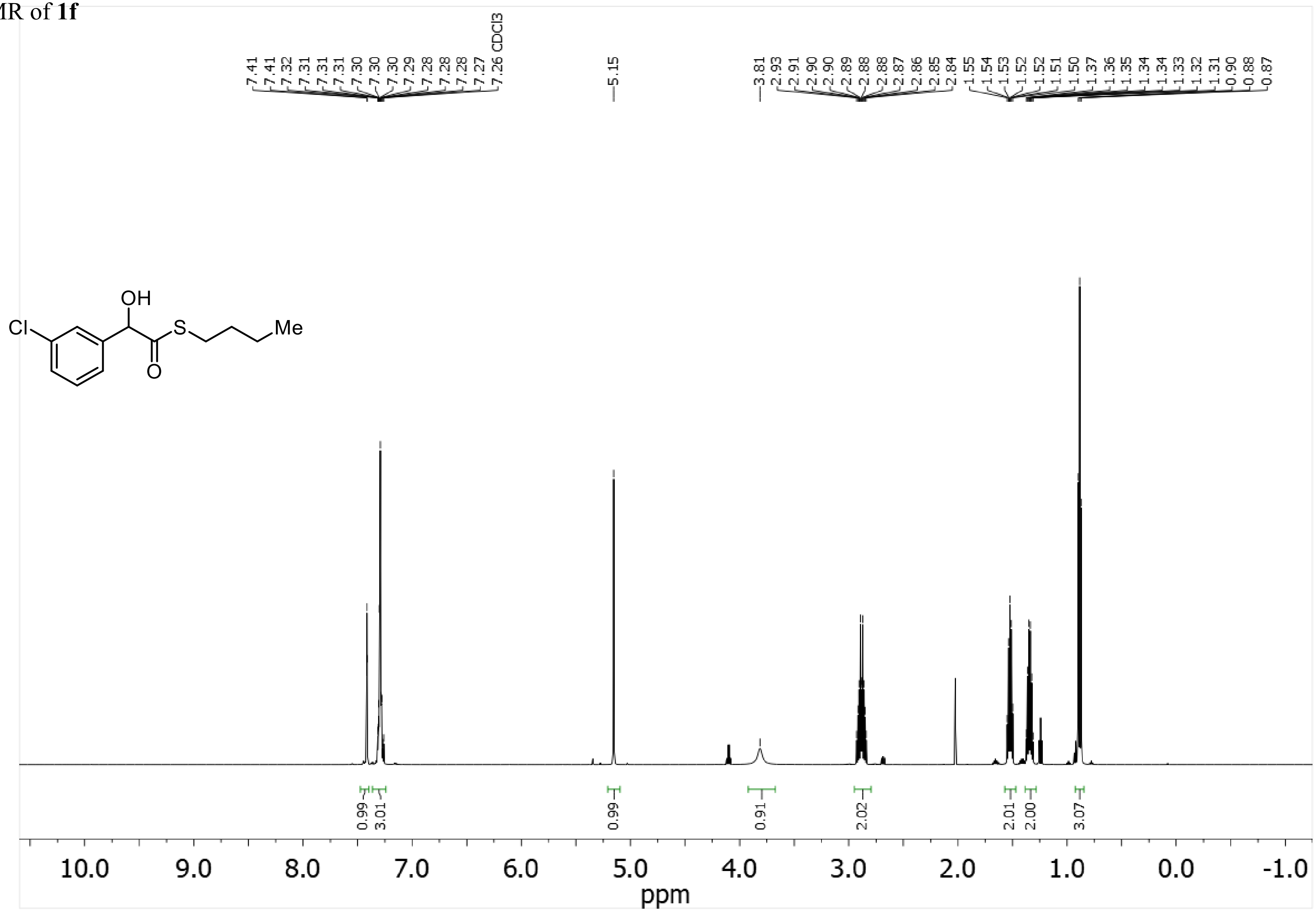

7.41
7.41
7.32
7.31
7.31
7.31
7.30
7.30
7.30
7.29
7.28
7.28
7.28
7.27
7.26 CDCl3
5.15
3.81
2.93
2.91
2.90
2.90
2.89
2.88
2.88
2.87
2.86
2.85
2.84
1.55
1.54
1.53
1.52
1.52
1.51
1.50
1.37
1.36
1.35
1.34
1.34
1.33
1.32
1.31
0.90
0.88
0.87
OH
Cl
S
Me
O
0.99
3.01
0.99
0.91
2.02
2.01
2.00
3.07
10.0
9.0
8.0
7.0
6.0
5.0
4.0
3.0
2.0
1.0
0.0
-1.0
ppm

^{13}C NMR of **1f**

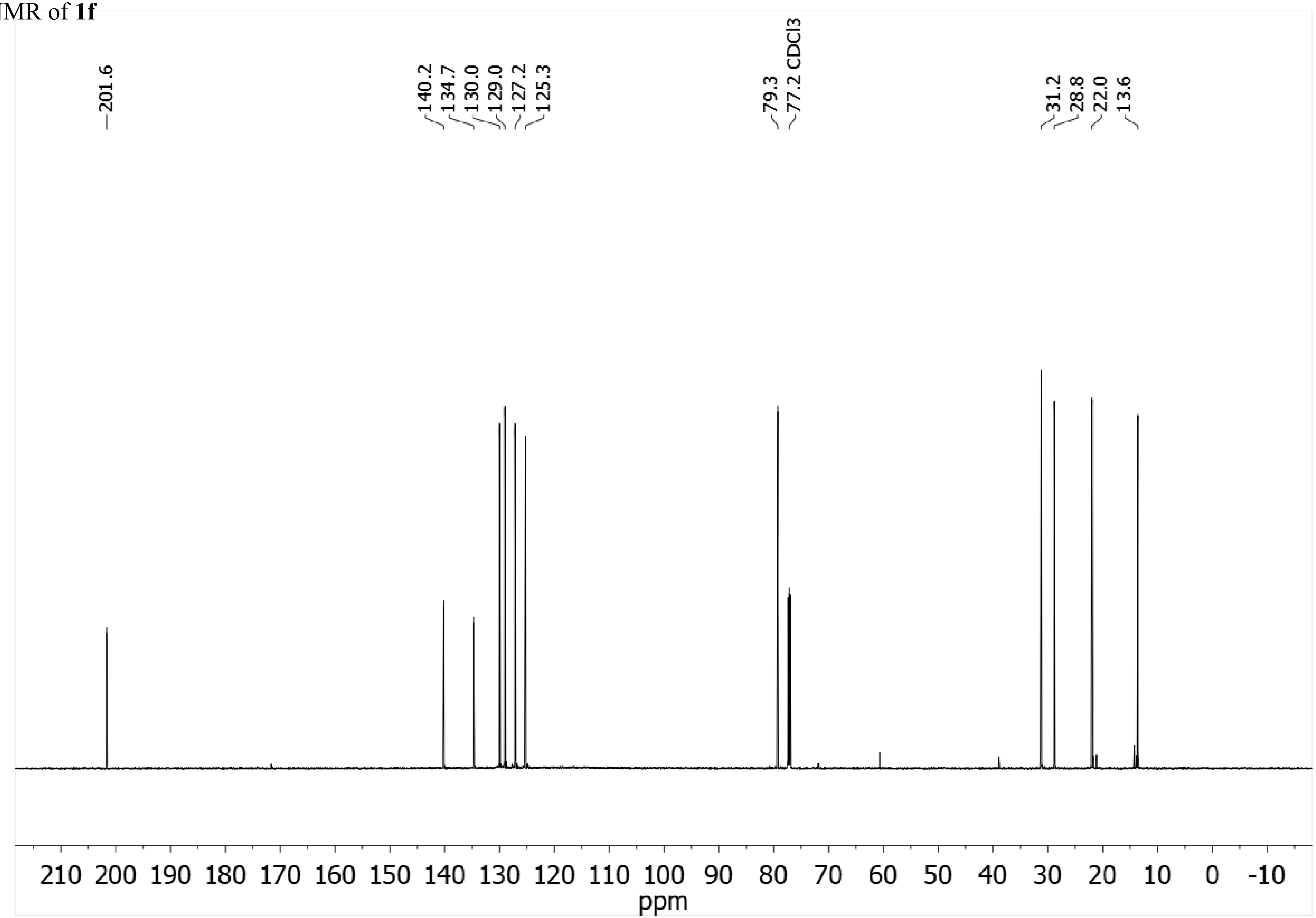

201.6
140.2
134.7
130.0
129.0
127.2
125.3
79.3
77.2 CDCl3
31.2
28.8
22.0
13.6
210 200 190 180 170 160 150 140 130 120 110 100 90 80 70 60 50 40 30 20 10 0 -10
ppm

^{1}H NMR of **1g**

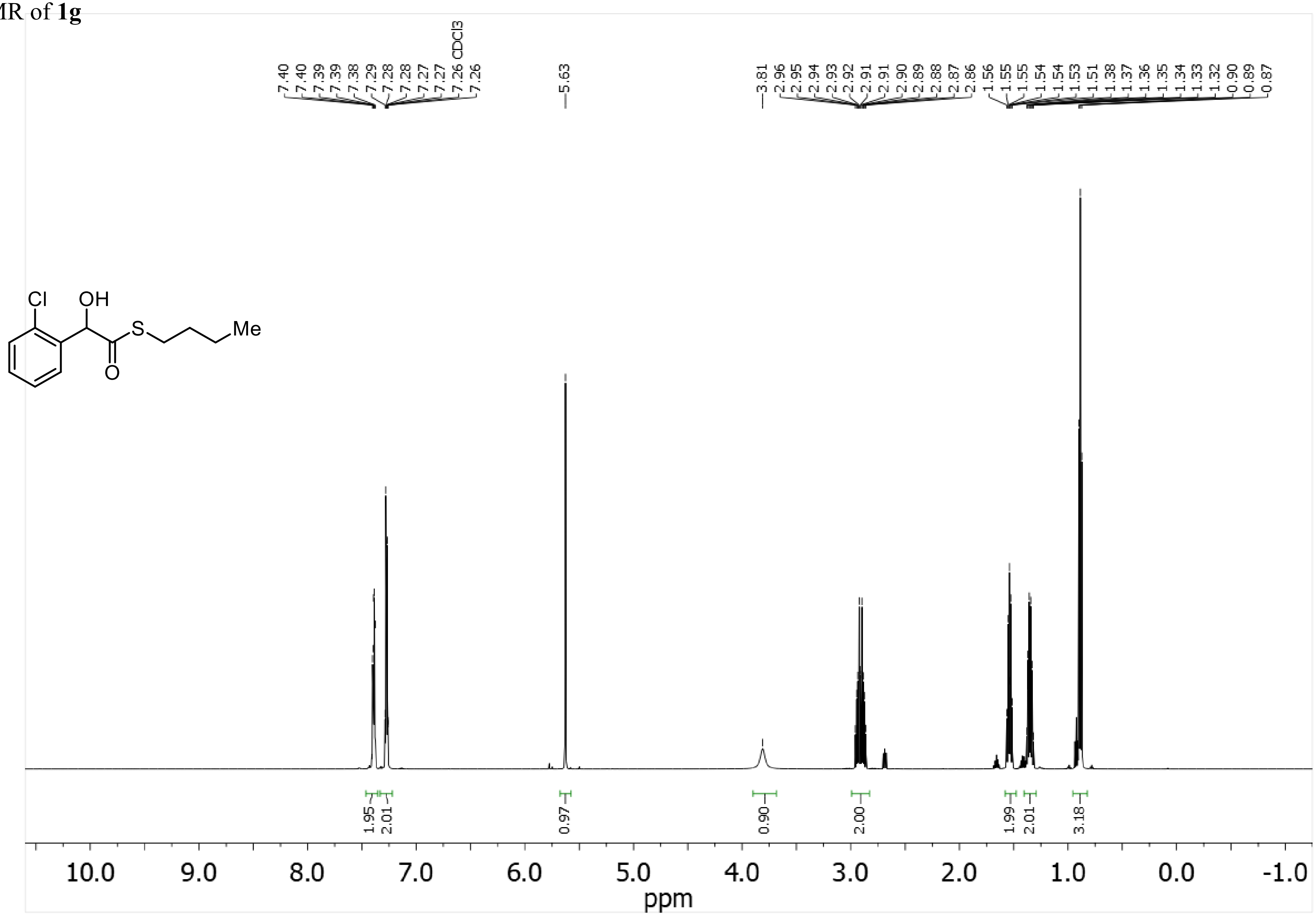

^{13}C NMR of **1g**

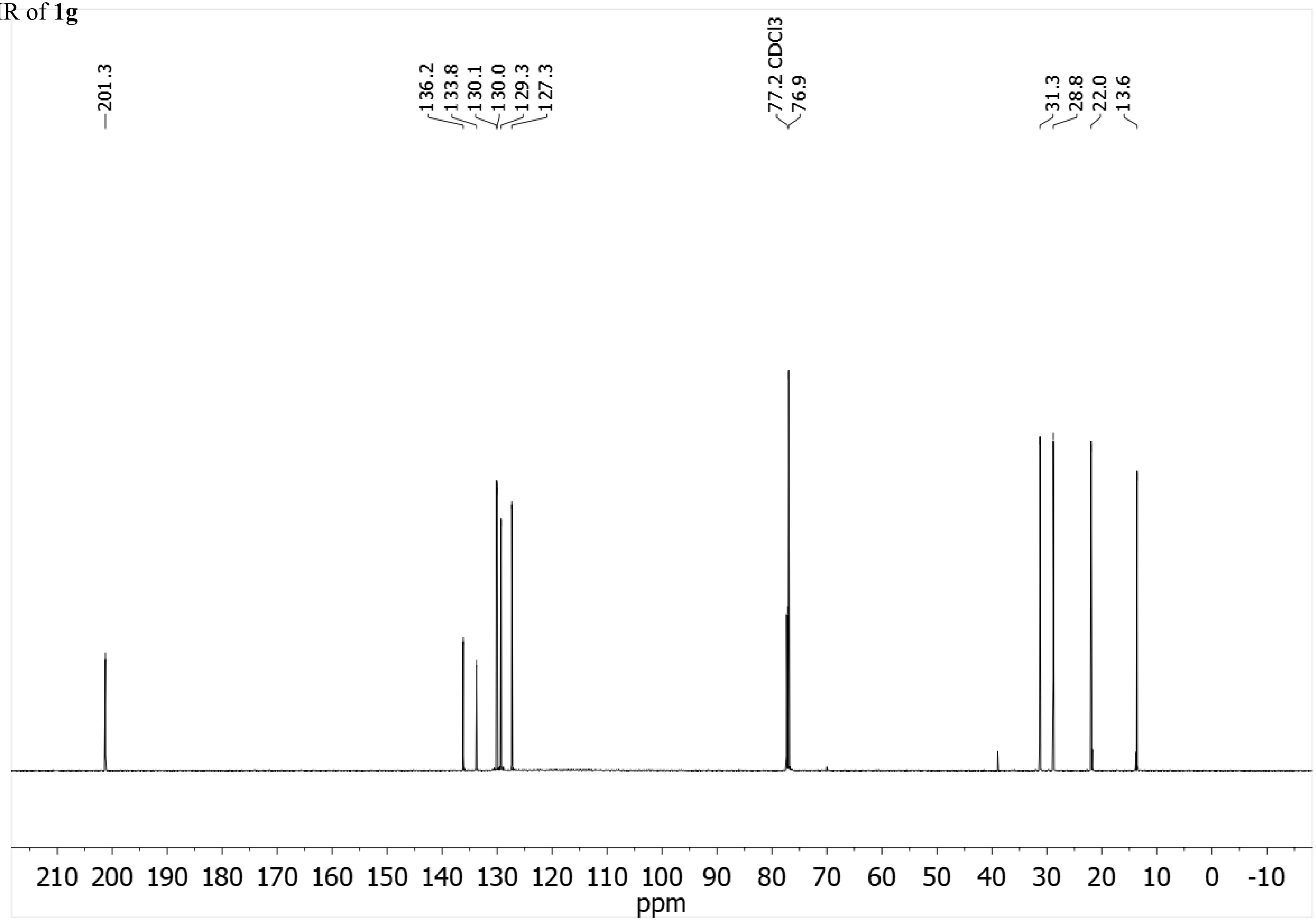

201.3
136.2
133.8
130.1
130.0
129.3
127.3
77.2 CDCl3
76.9
31.3
28.8
22.0
13.6
210 200 190 180 170 160 150 140 130 120 110 100 90 80 70 60 50 40 30 20 10 0 -10
ppm

^{1}H NMR of **1h**

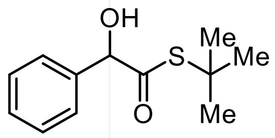
OH
S
Me
Me
O
Me

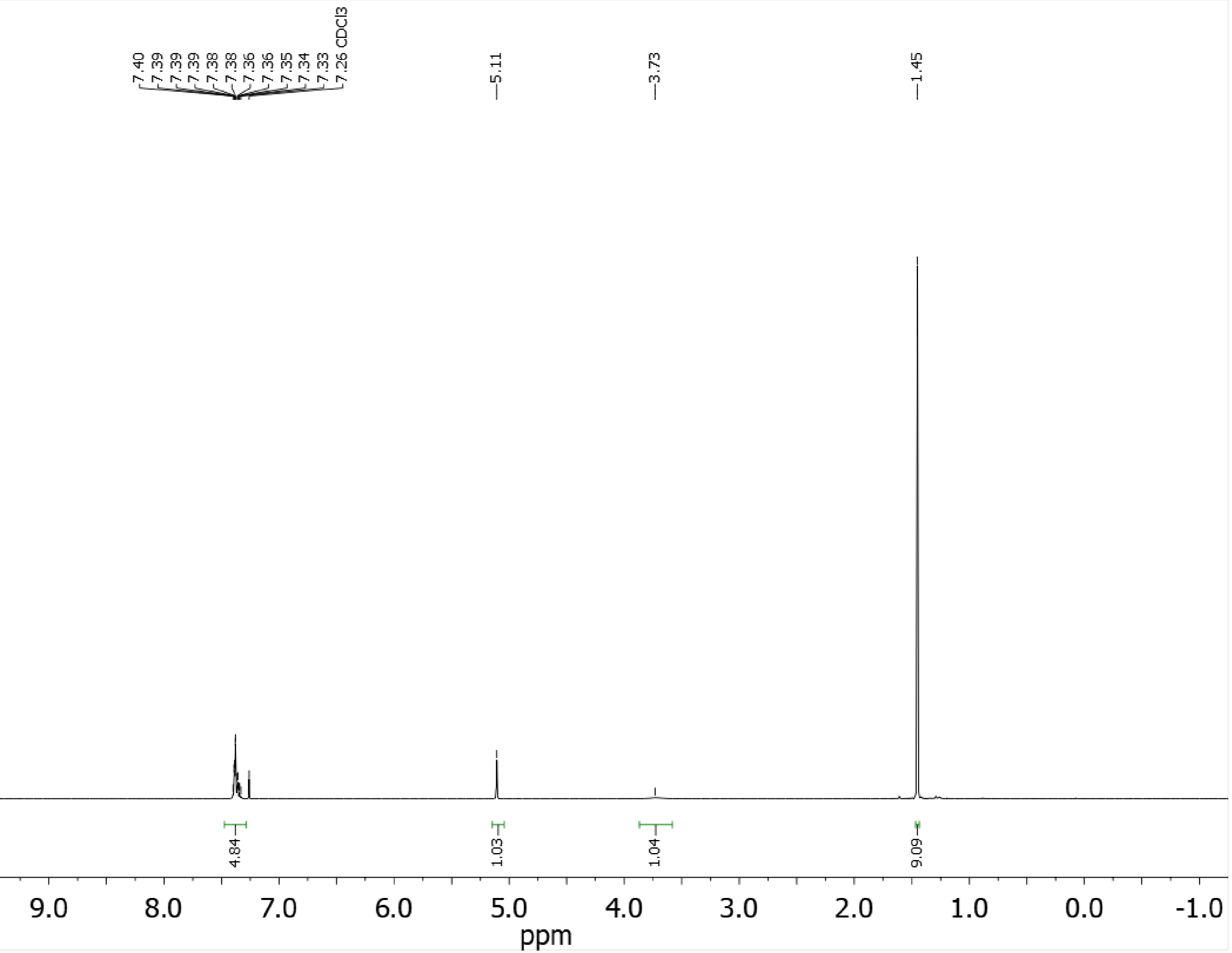
7.40
7.39
7.39
7.39
7.38
7.38
7.36
7.36
7.35
7.34
7.33
7.26 CDCl3
5.11
3.73
1.45
4.84
1.03
1.04
9.09
10.0
9.0
8.0
7.0
6.0
5.0
4.0
3.0
2.0
1.0
0.0
-1.0
ppm

^{1}H NMR of **1j**

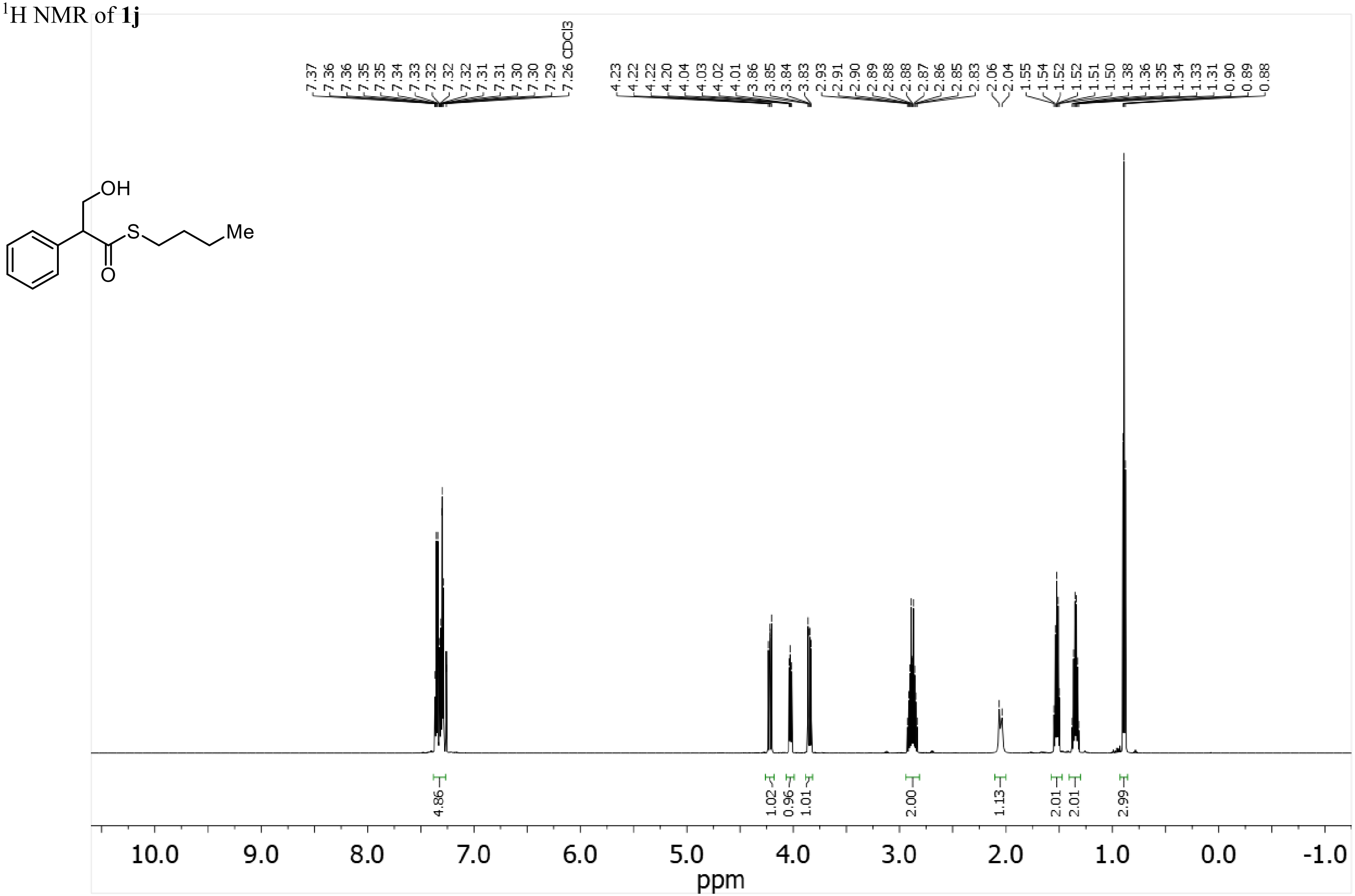

7.37
7.36
7.36
7.35
7.35
7.34
7.33
7.32
7.32
7.32
7.31
7.31
7.30
7.30
7.29
7.26 CDCl3
4.23
4.22
4.22
4.20
4.04
4.03
4.02
4.01
3.86
3.85
3.84
3.83
2.93
2.91
2.90
2.89
2.88
2.88
2.87
2.86
2.85
2.83
2.06
2.04
1.55
1.54
1.52
1.52
1.51
1.50
1.38
1.36
1.35
1.34
1.33
1.31
0.90
0.89
0.88
4.86
1.02
0.96
1.01
2.00
1.13
2.01
2.01
2.99
10.0
9.0
8.0
7.0
6.0
5.0
4.0
3.0
2.0
1.0
0.0
-1.0
ppm

^{13}C NMR of **1j**

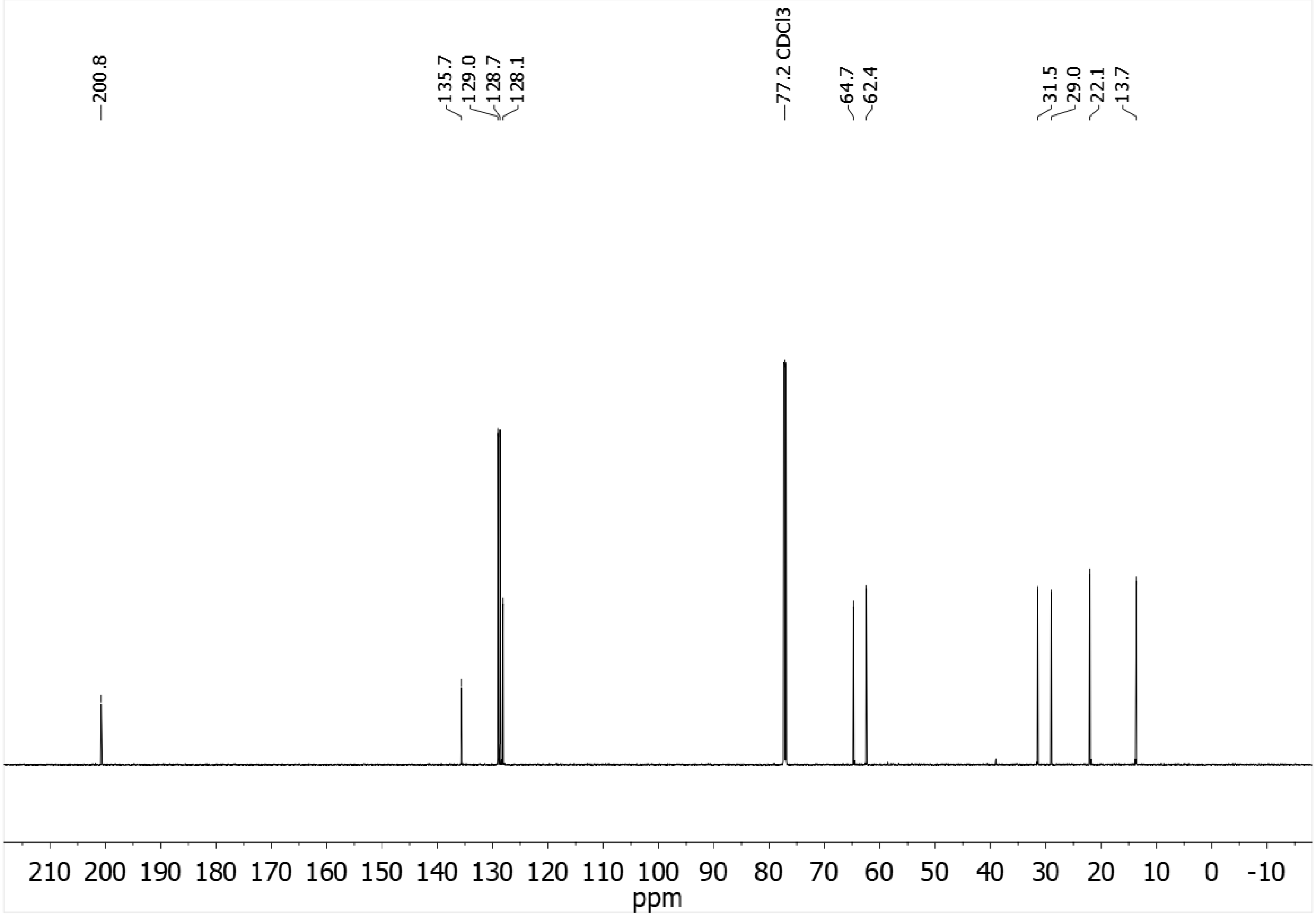

200.8
135.7
129.0
128.7
128.1
77.2 CDCl3
64.7
62.4
31.5
29.0
22.1
13.7
210 200 190 180 170 160 150 140 130 120 110 100 90 80 70 60 50 40 30 20 10 0 -10
ppm

^{1}H NMR of **1k**

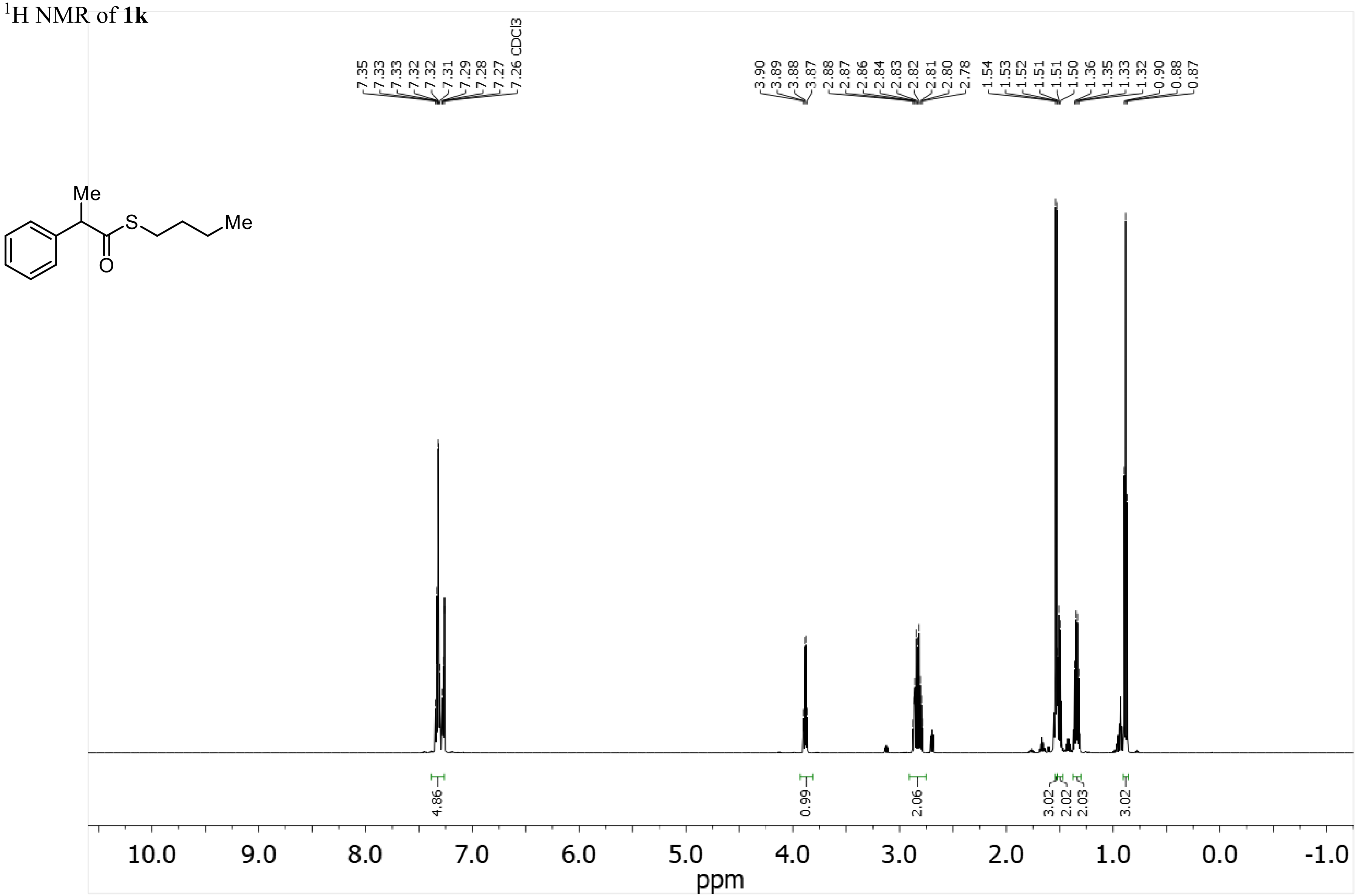

7.35
7.33
7.33
7.32
7.32
7.31
7.29
7.28
7.27
7.26 CDCl3
3.90
3.89
3.88
3.87
2.88
2.87
2.86
2.84
2.83
2.82
2.81
2.80
2.78
1.54
1.53
1.52
1.51
1.51
1.50
1.36
1.35
1.33
1.32
0.90
0.88
0.87
4.86
0.99
2.06
3.02
2.02
2.03
3.02
10.0
9.0
8.0
7.0
6.0
5.0
4.0
3.0
2.0
1.0
0.0
-1.0
ppm

^{13}C NMR of **1k**

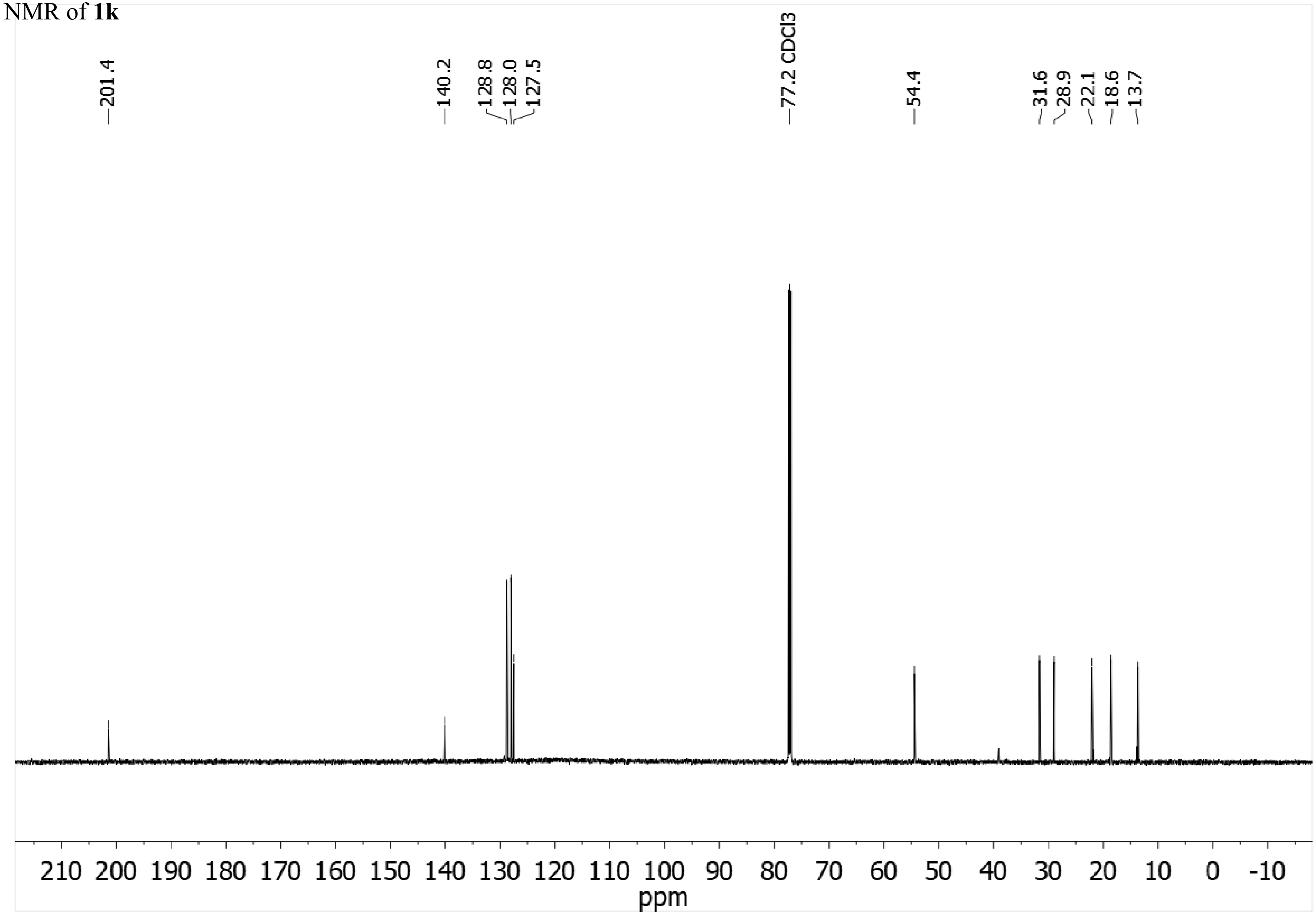

201.4
140.2
128.8
128.0
127.5
77.2 CDCl3
54.4
31.6
28.9
22.1
18.6
13.7
210 200 190 180 170 160 150 140 130 120 110 100 90 80 70 60 50 40 30 20 10 0 -10
ppm

SI.17 MS spectra of purified proteins

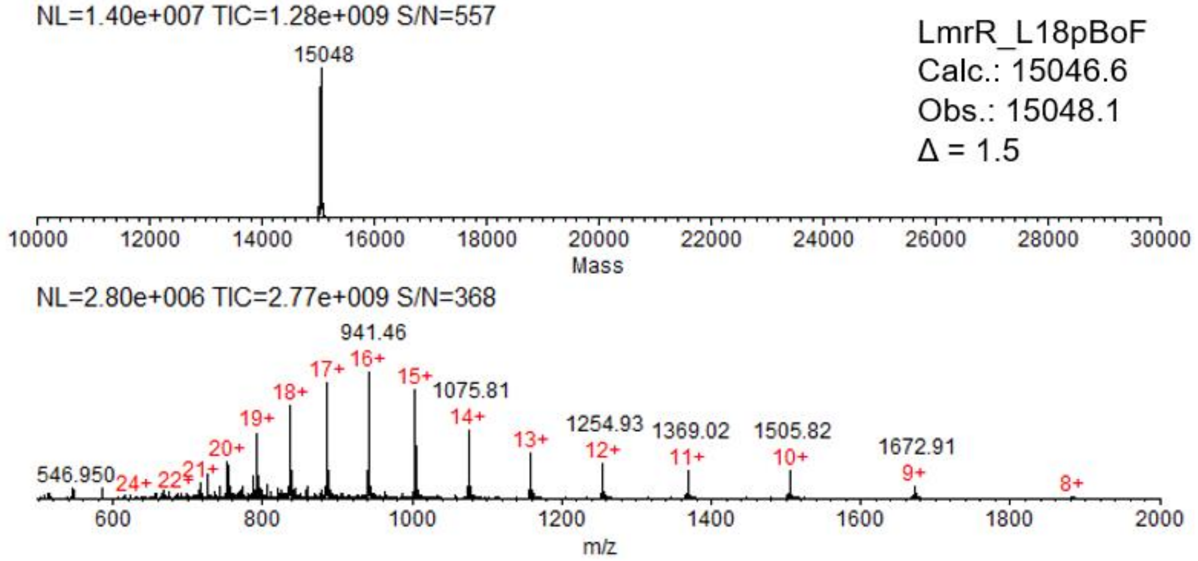

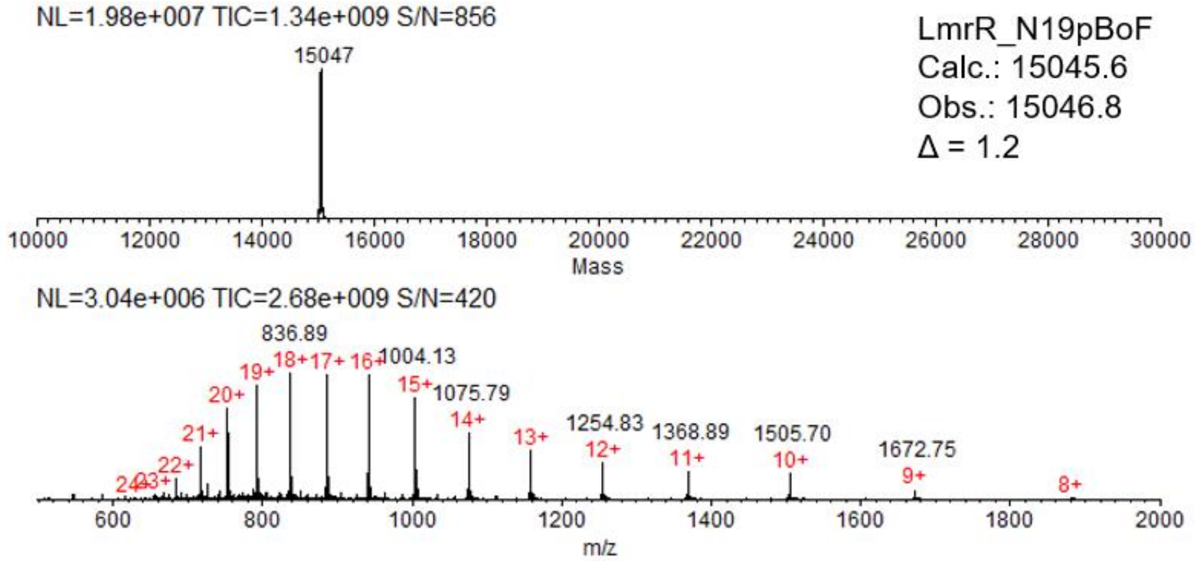

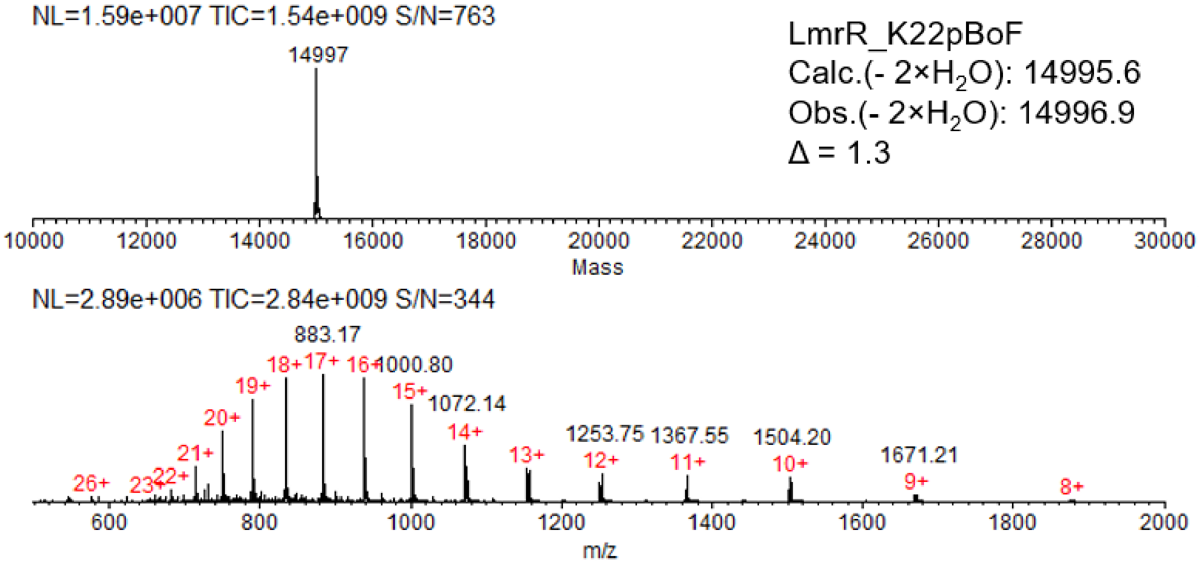

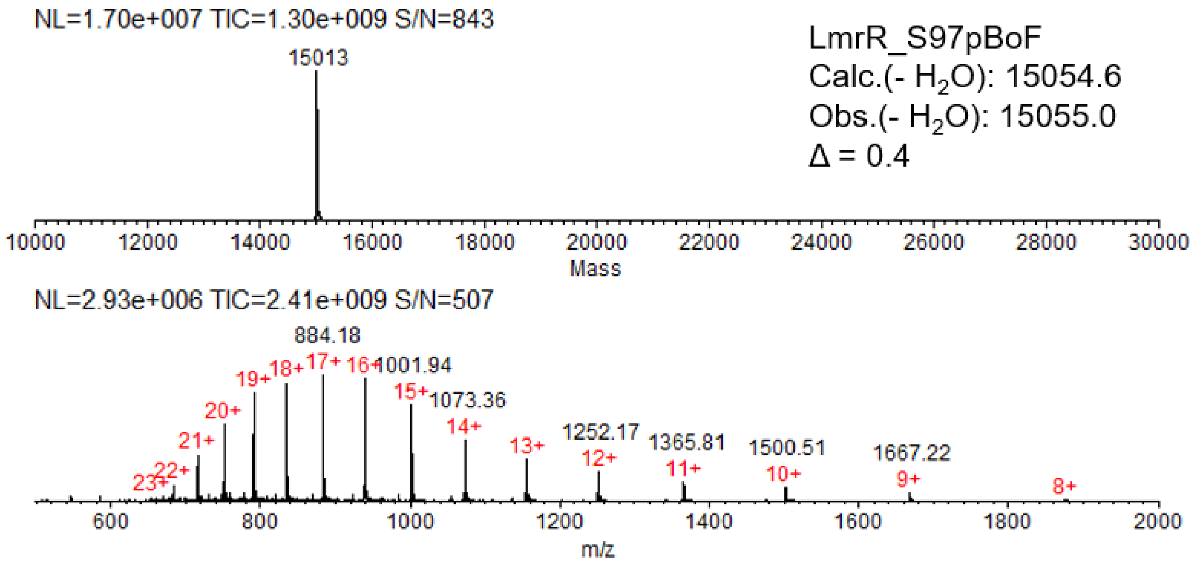
NL=1.70e+007 TIC=1.30e+009 S/N=843
15013
LmrR_S97pBoF
Calc.(- H_2O): 15054.6
Obs.(- H_2O): 15055.0
Δ = 0.4
Mass
NL=2.93e+006 TIC=2.41e+009 S/N=507
884.18
1001.94
1073.36
1252.17
1365.81
1500.51
1667.22
23+
22+
21+
20+
19+
18+
17+
16+
15+
14+
13+
12+
11+
10+
9+
8+
m/z

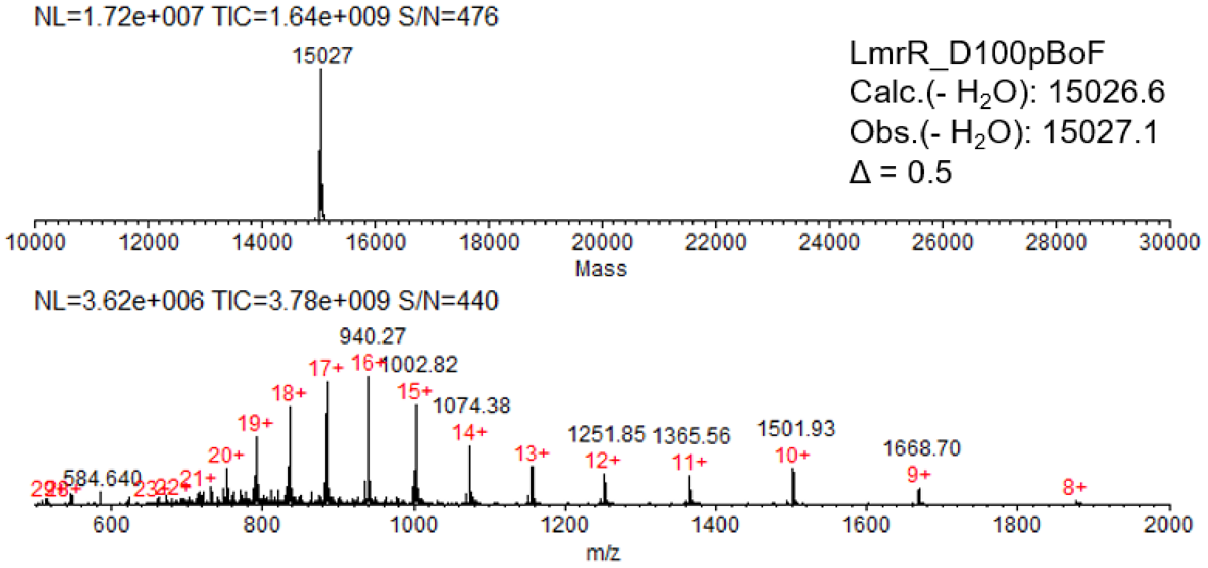
NL=1.72e+007 TIC=1.64e+009 S/N=476
15027
LmrR_D100pBoF
Calc.(- H_2O): 15026.6
Obs.(- H_2O): 15027.1
Δ = 0.5
Mass
NL=3.62e+006 TIC=3.78e+009 S/N=440
940.27
1002.82
1074.38
1251.85
1365.56
1501.93
1668.70
584.640
21+
20+
19+
18+
17+
16+
15+
14+
13+
12+
11+
10+
9+
8+
m/z

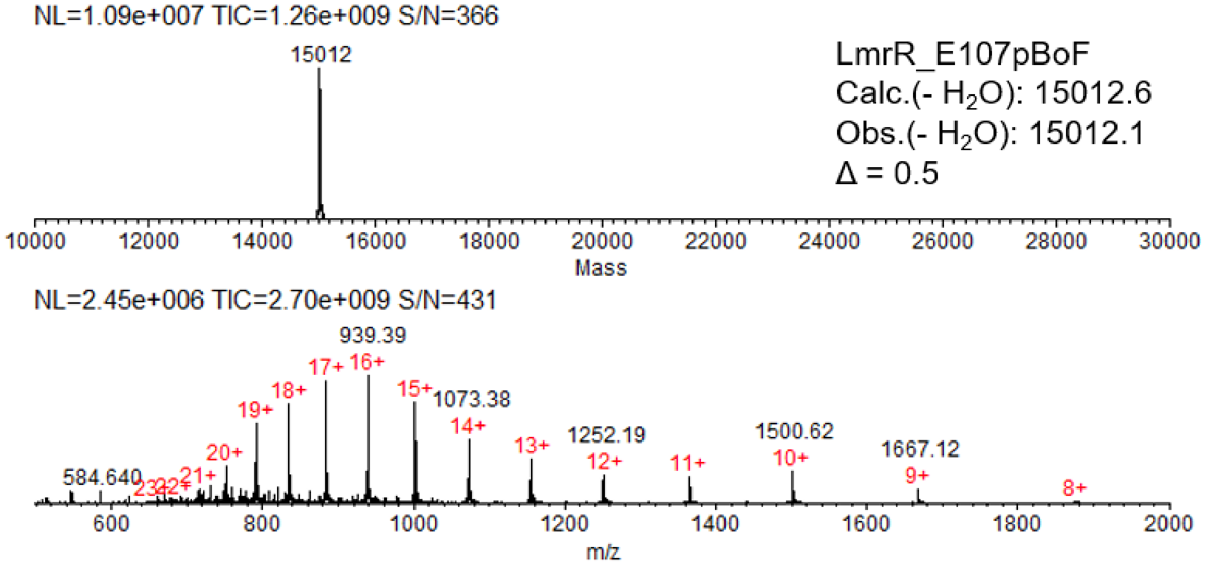
NL=1.09e+007 TIC=1.26e+009 S/N=366
15012
LmrR_E107pBoF
Calc.(- H_2O): 15012.6
Obs.(- H_2O): 15012.1
Δ = 0.5
Mass
NL=2.45e+006 TIC=2.70e+009 S/N=431
939.39
1073.38
1252.19
1500.62
1667.12
584.640
21+
20+
19+
18+
17+
16+
15+
14+
13+
12+
11+
10+
9+
8+
m/z

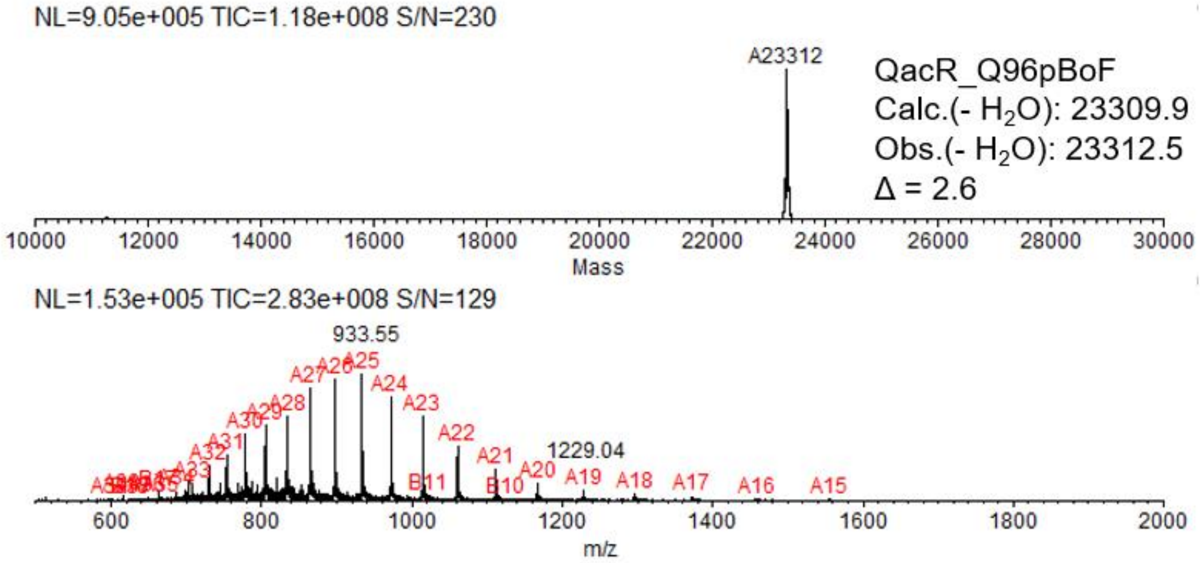
NL=9.05e+005 TIC=1.18e+008 S/N=230
A23312
QacR_Q96pBoF
Calc.(- H_2O): 23309.9
Obs.(- H_2O): 23312.5
Δ = 2.6
Mass
NL=1.53e+005 TIC=2.83e+008 S/N=129
933.55
1229.04
m/z

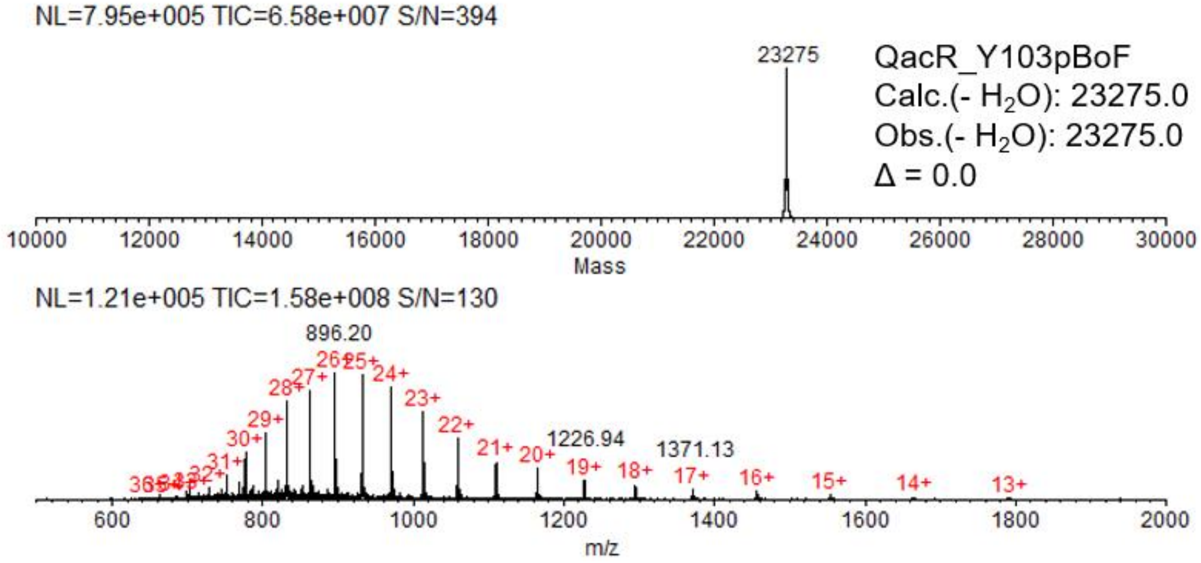
NL=7.95e+005 TIC=6.58e+007 S/N=394
23275
QacR_Y103pBoF
Calc.(- H_2O): 23275.0
Obs.(- H_2O): 23275.0
Δ = 0.0
Mass
NL=1.21e+005 TIC=1.58e+008 S/N=130
896.20
1226.94
1371.13
m/z

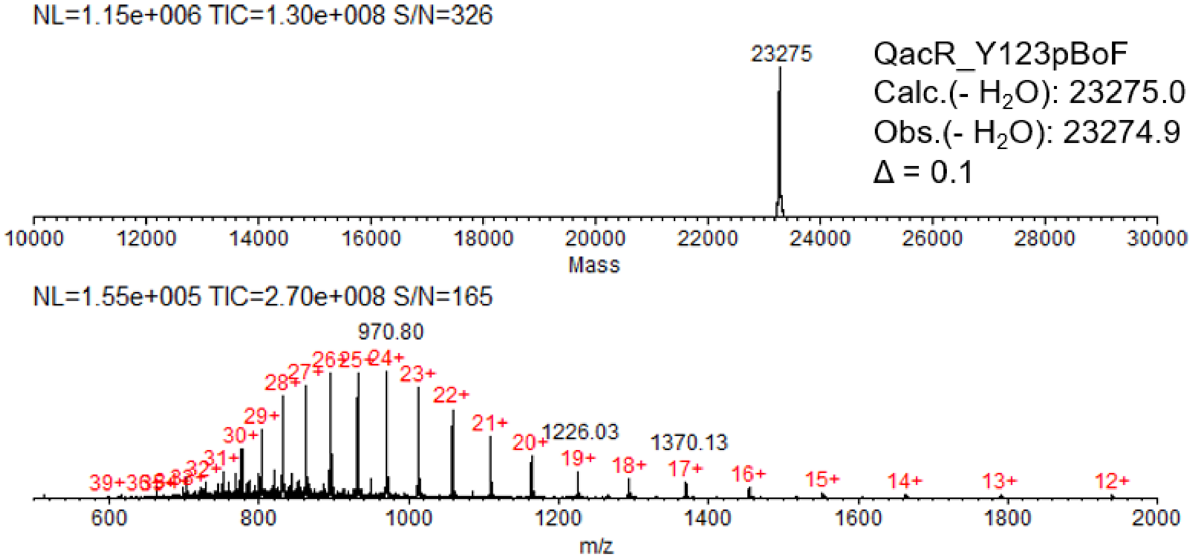
NL=1.15e+006 TIC=1.30e+008 S/N=326
23275
QacR_Y123pBoF
Calc.(- H_2O): 23275.0
Obs.(- H_2O): 23274.9
Δ = 0.1
Mass
NL=1.55e+005 TIC=2.70e+008 S/N=165
970.80
1226.03
1370.13
m/z

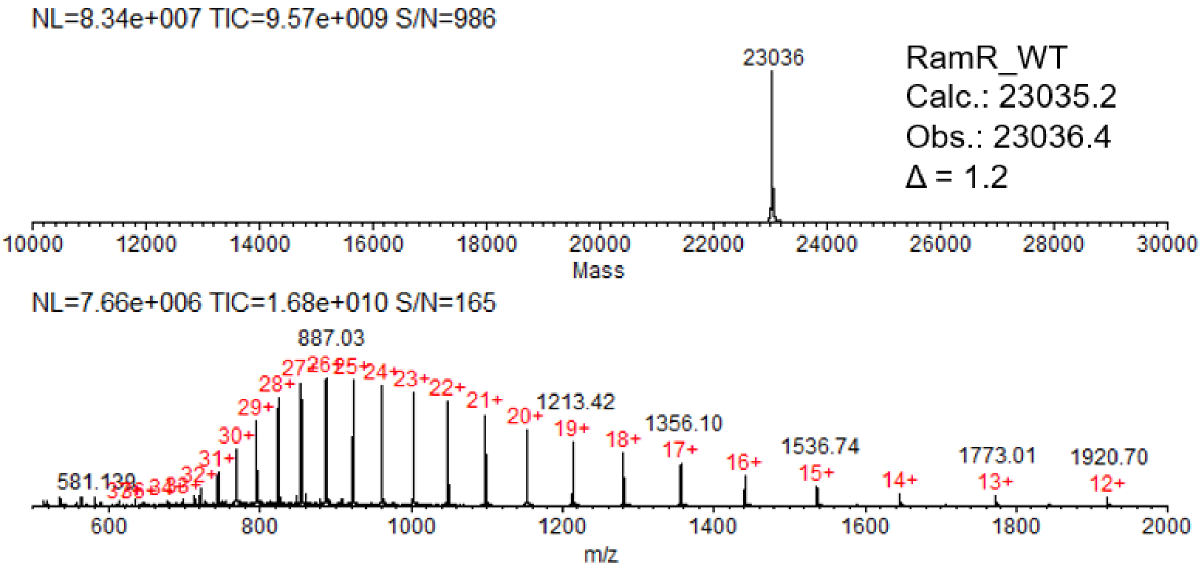
NL=8.34e+007 TIC=9.57e+009 S/N=986
23036
RamR_WT
Calc.: 23035.2
Obs.: 23036.4
Δ = 1.2
Mass
NL=7.66e+006 TIC=1.68e+010 S/N=165
887.03
1213.42
1356.10
1536.74
1773.01
1920.70
581.139
m/z

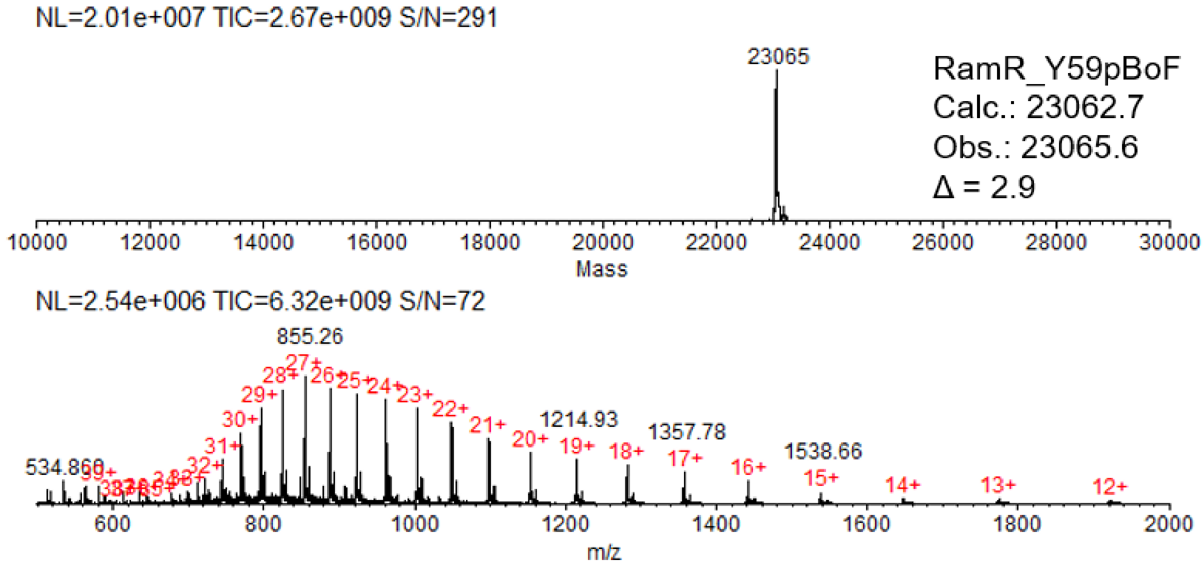
NL=2.01e+007 TIC=2.67e+009 S/N=291
23065
RamR_Y59pBoF
Calc.: 23062.7
Obs.: 23065.6
Δ = 2.9
Mass
NL=2.54e+006 TIC=6.32e+009 S/N=72
855.26
1214.93
1357.78
1538.66
m/z

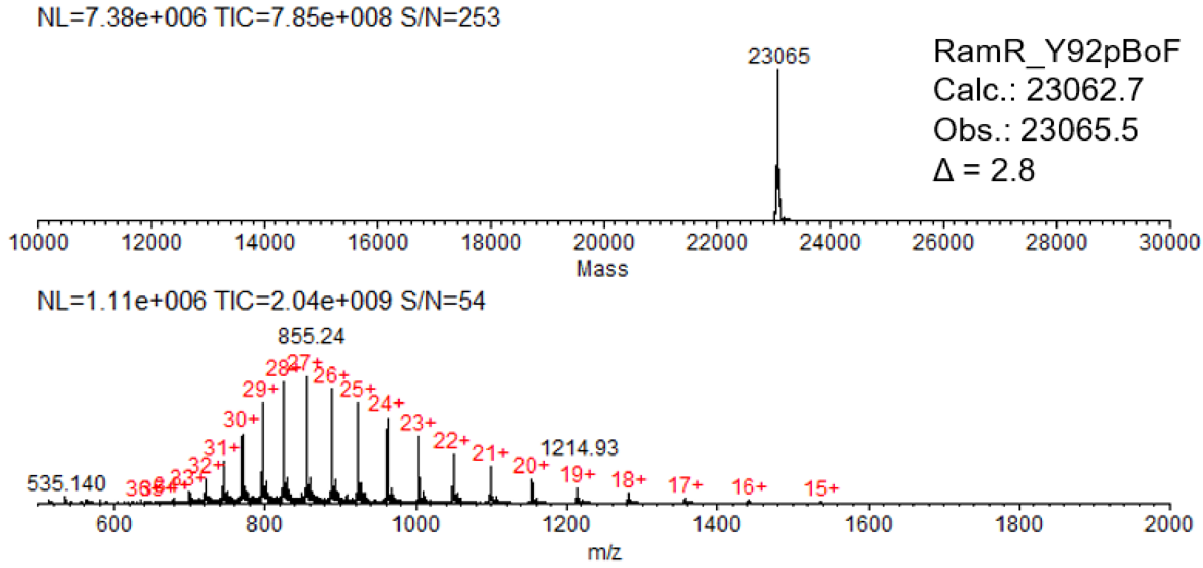
NL=7.38e+006 TIC=7.85e+008 S/N=253
23065
RamR_Y92pBoF
Calc.: 23062.7
Obs.: 23065.5
Δ = 2.8
Mass
NL=1.11e+006 TIC=2.04e+009 S/N=54
855.24
1214.93
535.140
m/z

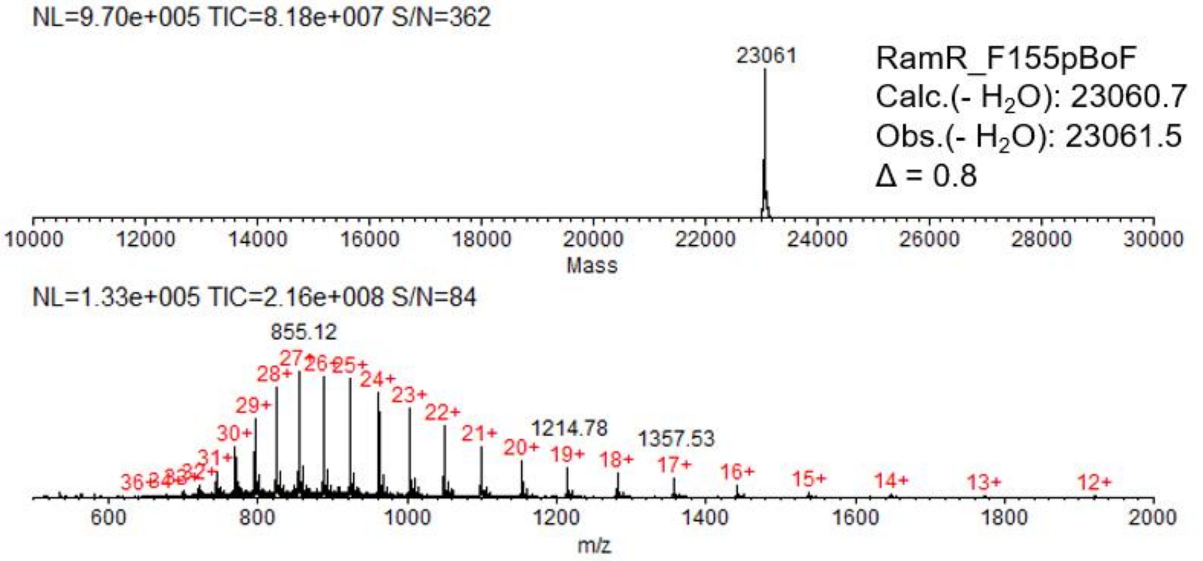
NL=9.70e+005 TIC=8.18e+007 S/N=362
23061
RamR_F155pBoF
Calc.(- H_2O): 23060.7
Obs.(- H_2O): 23061.5
Δ = 0.8
10000
12000
14000
16000
18000
20000
22000
24000
26000
28000
30000
Mass
NL=1.33e+005 TIC=2.16e+008 S/N=84
855.12
28+
27+
26+
25+
24+
23+
22+
21+
20+
19+
18+
17+
16+
15+
14+
13+
12+
29+
30+
31+
1214.78
1357.53
600
800
1000
1200
1400
1600
1800
2000
m/z

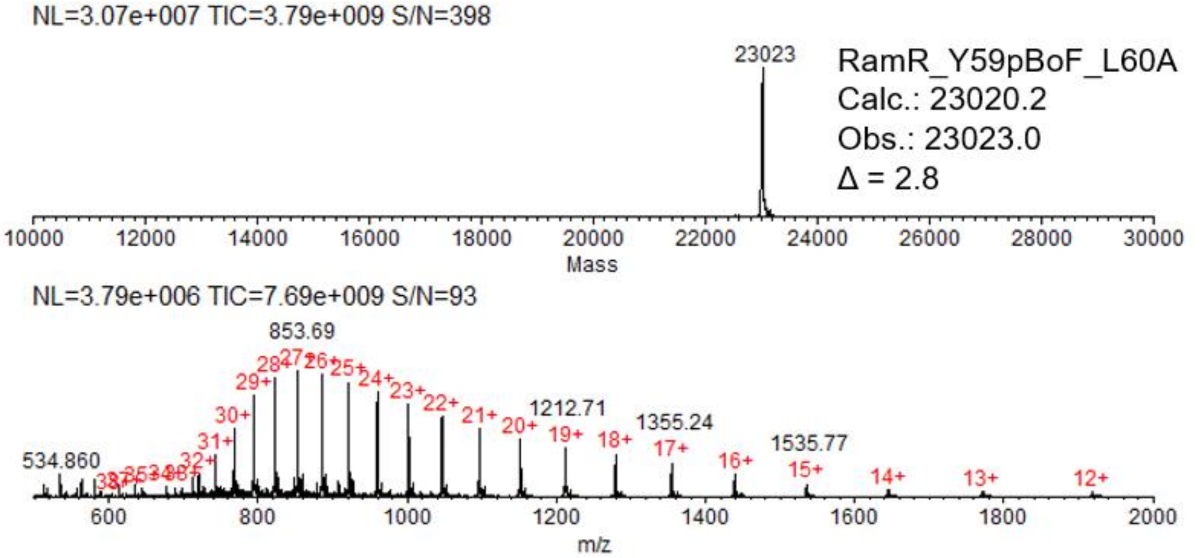
NL=3.07e+007 TIC=3.79e+009 S/N=398
23023
RamR_Y59pBoF_L60A
Calc.: 23020.2
Obs.: 23023.0
Δ = 2.8
10000
12000
14000
16000
18000
20000
22000
24000
26000
28000
30000
Mass
NL=3.79e+006 TIC=7.69e+009 S/N=93
853.69
534.860
1212.71
1355.24
1535.77
28+
27+
26+
25+
24+
23+
22+
21+
20+
19+
18+
17+
16+
15+
14+
13+
12+
29+
30+
31+
32+
600
800
1000
1200
1400
1600
1800
2000
m/z

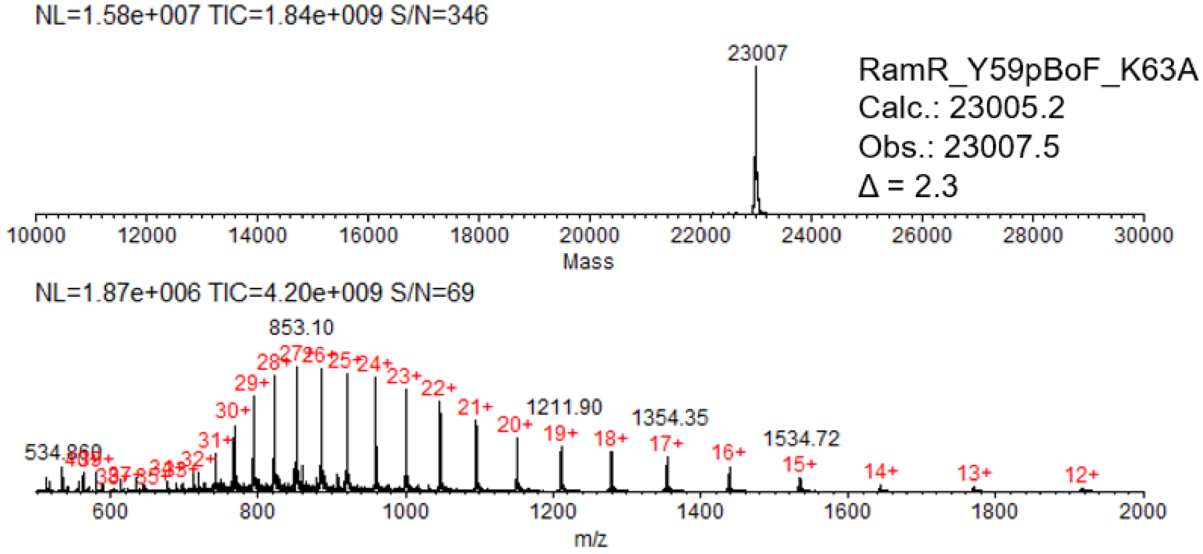
NL=1.58e+007 TIC=1.84e+009 S/N=346
23007
RamR_Y59pBoF_K63A
Calc.: 23005.2
Obs.: 23007.5
Δ = 2.3
10000
12000
14000
16000
18000
20000
22000
24000
26000
28000
30000
Mass
NL=1.87e+006 TIC=4.20e+009 S/N=69
853.10
534.860
1211.90
1354.35
1534.72
28+
27+
26+
25+
24+
23+
22+
21+
20+
19+
18+
17+
16+
15+
14+
13+
12+
29+
30+
31+
32+
600
800
1000
1200
1400
1600
1800
2000
m/z

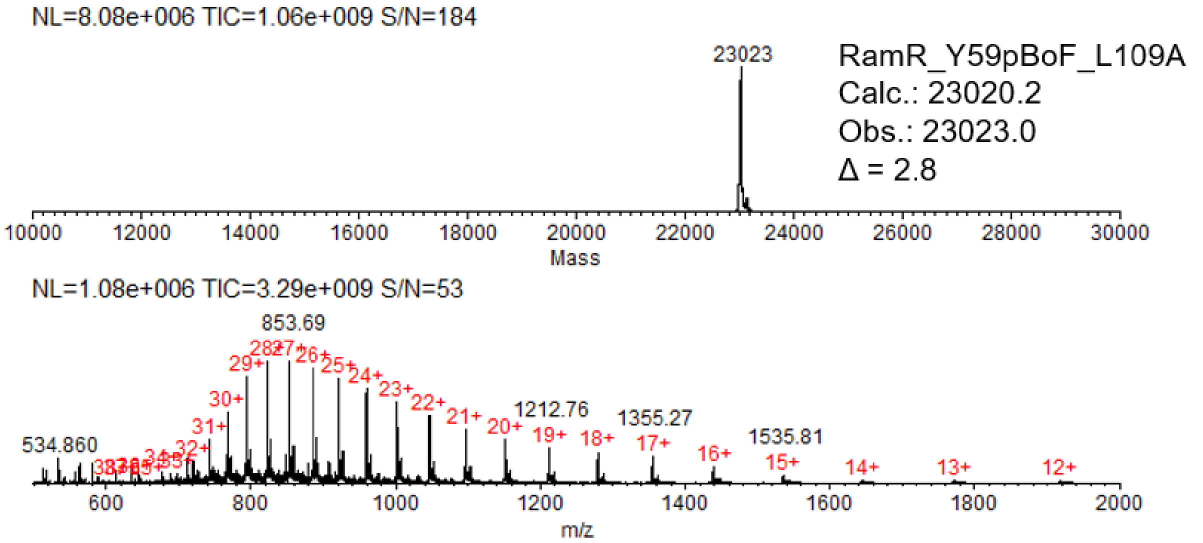
NL=8.08e+006 TIC=1.06e+009 S/N=184
23023
RamR_Y59pBoF_L109A
Calc.: 23020.2
Obs.: 23023.0
Δ = 2.8
10000 12000 14000 16000 18000 20000 22000 24000 26000 28000 30000
Mass
NL=1.08e+006 TIC=3.29e+009 S/N=53
853.69
1212.76
1355.27
1535.81
534.860
29+ 26+ 25+ 24+ 23+ 22+ 21+ 20+ 19+ 18+ 17+ 16+ 15+ 14+ 13+ 12+
30+ 31+ 32+
600 800 1000 1200 1400 1600 1800 2000
m/z

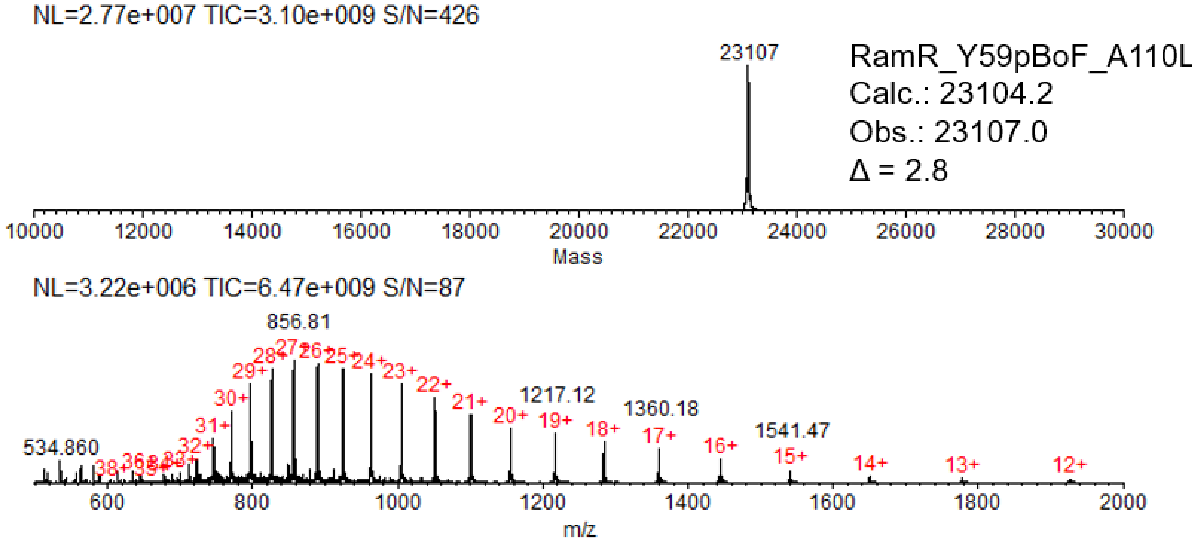
NL=2.77e+007 TIC=3.10e+009 S/N=426
23107
RamR_Y59pBoF_A110L
Calc.: 23104.2
Obs.: 23107.0
Δ = 2.8
10000 12000 14000 16000 18000 20000 22000 24000 26000 28000 30000
Mass
NL=3.22e+006 TIC=6.47e+009 S/N=87
856.81
1217.12
1360.18
1541.47
534.860
28+ 29+ 25+ 24+ 23+ 22+ 21+ 20+ 19+ 18+ 17+ 16+ 15+ 14+ 13+ 12+
30+ 31+ 32+
600 800 1000 1200 1400 1600 1800 2000
m/z

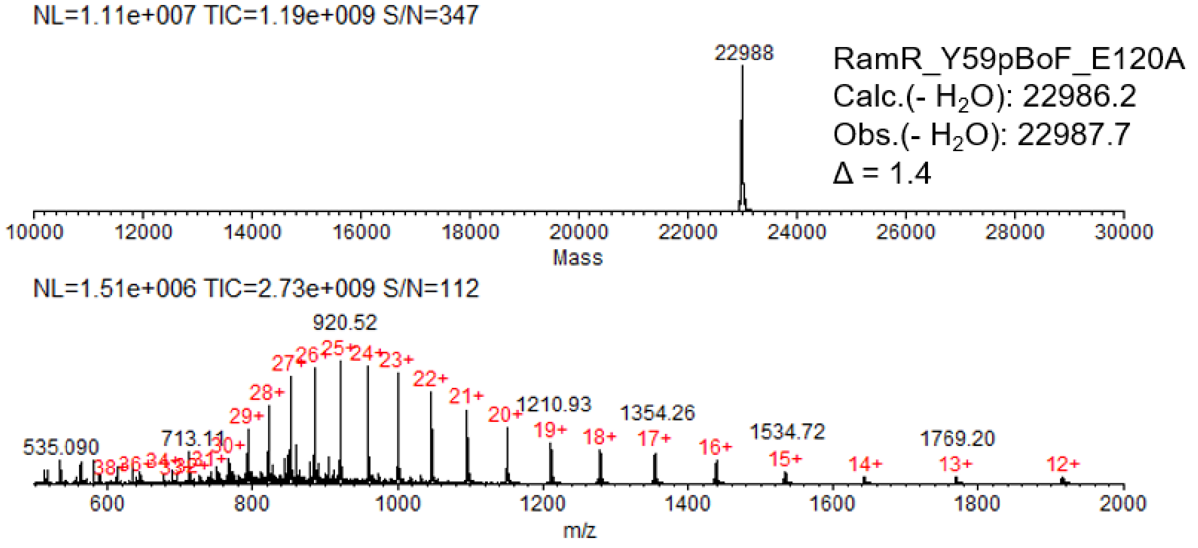
NL=1.11e+007 TIC=1.19e+009 S/N=347
22988
RamR_Y59pBoF_E120A
Calc.(- H_2O): 22986.2
Obs.(- H_2O): 22987.7
Δ = 1.4
10000 12000 14000 16000 18000 20000 22000 24000 26000 28000 30000
Mass
NL=1.51e+006 TIC=2.73e+009 S/N=112
920.52
1210.93
1354.26
1534.72
1769.20
535.090
713.11
25+ 24+ 23+ 22+ 21+ 20+ 19+ 18+ 17+ 16+ 15+ 14+ 13+ 12+
28+ 29+
600 800 1000 1200 1400 1600 1800 2000
m/z

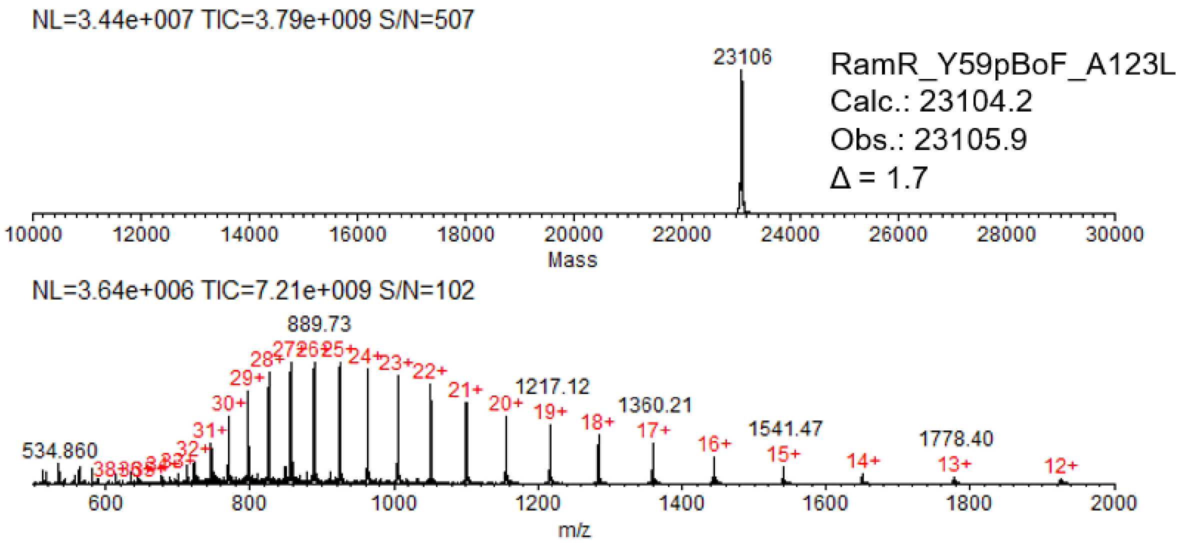
NL=3.44e+007 TIC=3.79e+009 S/N=507
23106
RamR_Y59pBoF_A123L
Calc.: 23104.2
Obs.: 23105.9
Δ = 1.7
Mass
NL=3.64e+006 TIC=7.21e+009 S/N=102
889.73
1217.12
1360.21
1541.47
1778.40
534.860
m/z

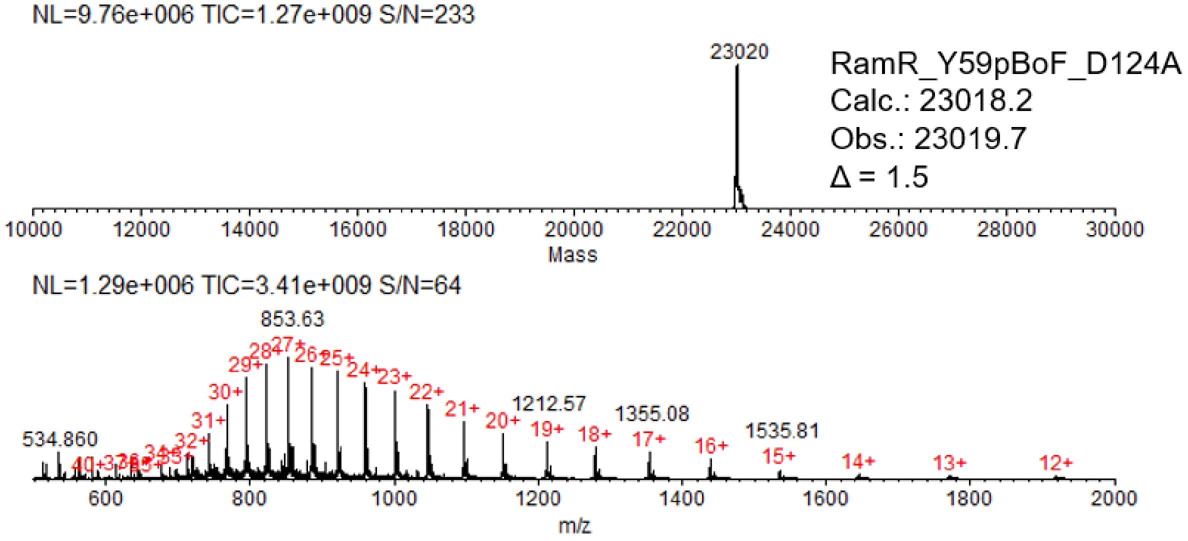
NL=9.76e+006 TIC=1.27e+009 S/N=233
23020
RamR_Y59pBoF_D124A
Calc.: 23018.2
Obs.: 23019.7
Δ = 1.5
Mass
NL=1.29e+006 TIC=3.41e+009 S/N=64
853.63
1212.57
1355.08
1535.81
534.860
m/z

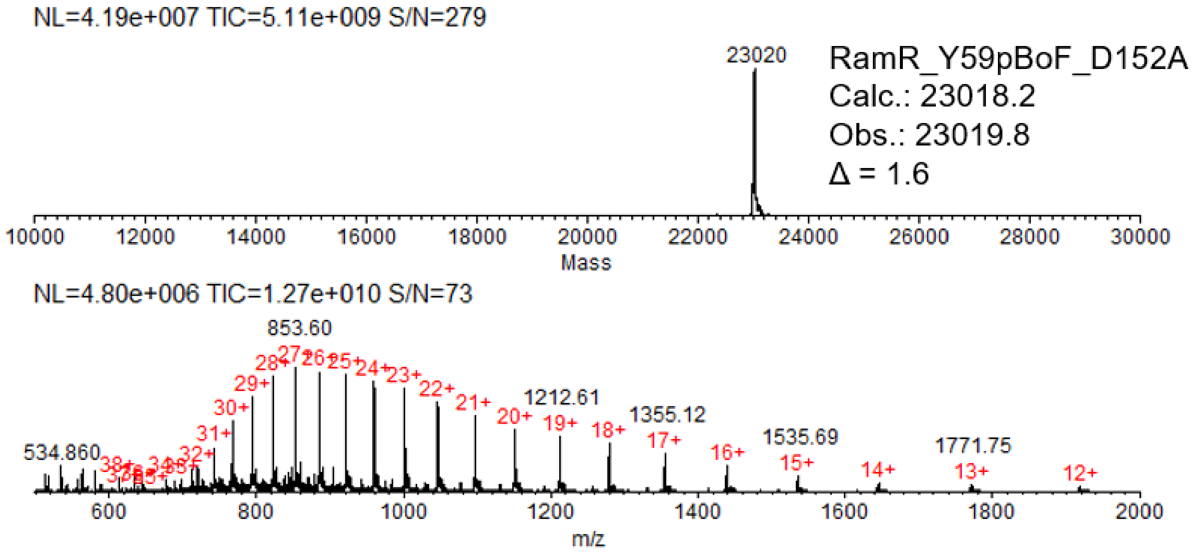
NL=4.19e+007 TIC=5.11e+009 S/N=279
23020
RamR_Y59pBoF_D152A
Calc.: 23018.2
Obs.: 23019.8
Δ = 1.6
Mass
NL=4.80e+006 TIC=1.27e+010 S/N=73
853.60
1212.61
1355.12
1535.69
1771.75
534.860
m/z

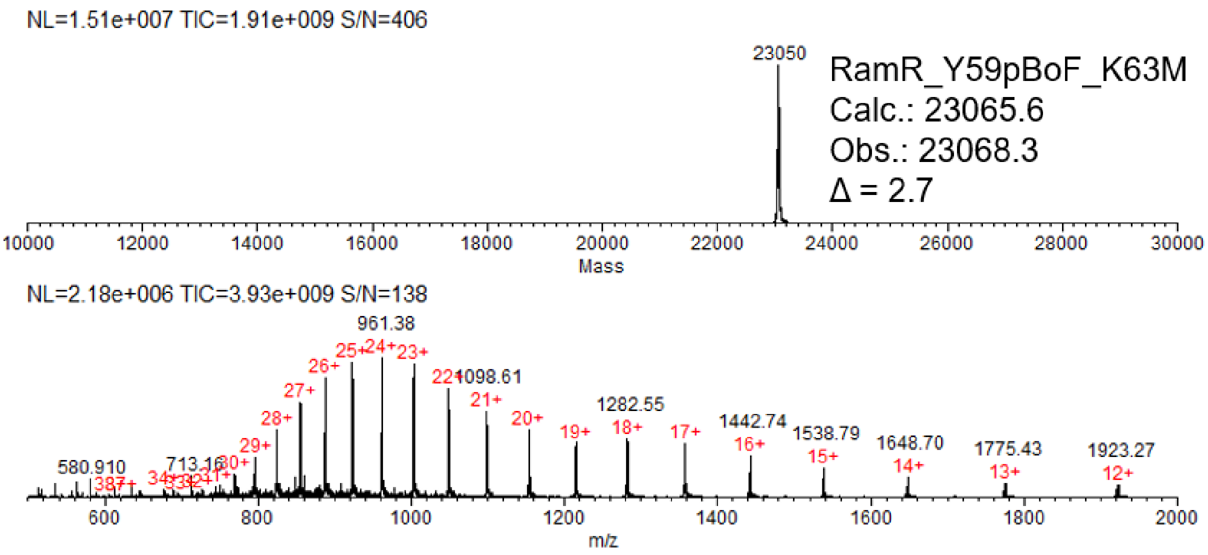
NL=1.51e+007 TIC=1.91e+009 S/N=406
23050
RamR_Y59pBoF_K63M
Calc.: 23065.6
Obs.: 23068.3
Δ = 2.7
Mass
NL=2.18e+006 TIC=3.93e+009 S/N=138
961.38
1098.61
1282.55
1442.74
1538.79
1648.70
1775.43
1923.27
580.910
m/z

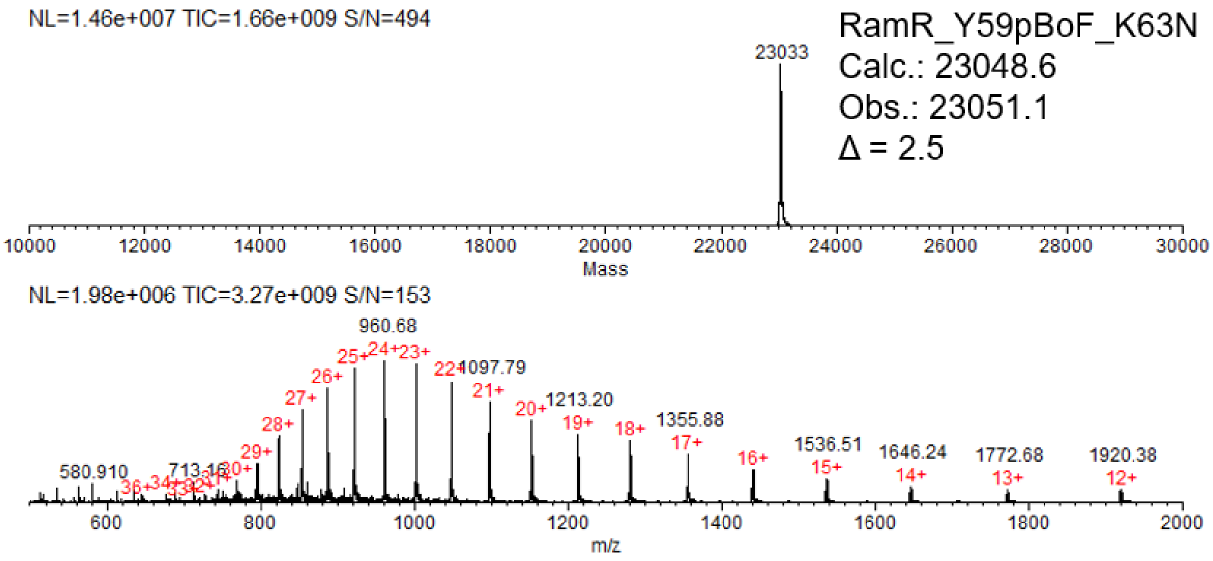
NL=1.46e+007 TIC=1.66e+009 S/N=494
23033
RamR_Y59pBoF_K63N
Calc.: 23048.6
Obs.: 23051.1
Δ = 2.5
Mass
NL=1.98e+006 TIC=3.27e+009 S/N=153
960.68
1097.79
1213.20
1355.88
1536.51
1646.24
1772.68
1920.38
580.910
m/z

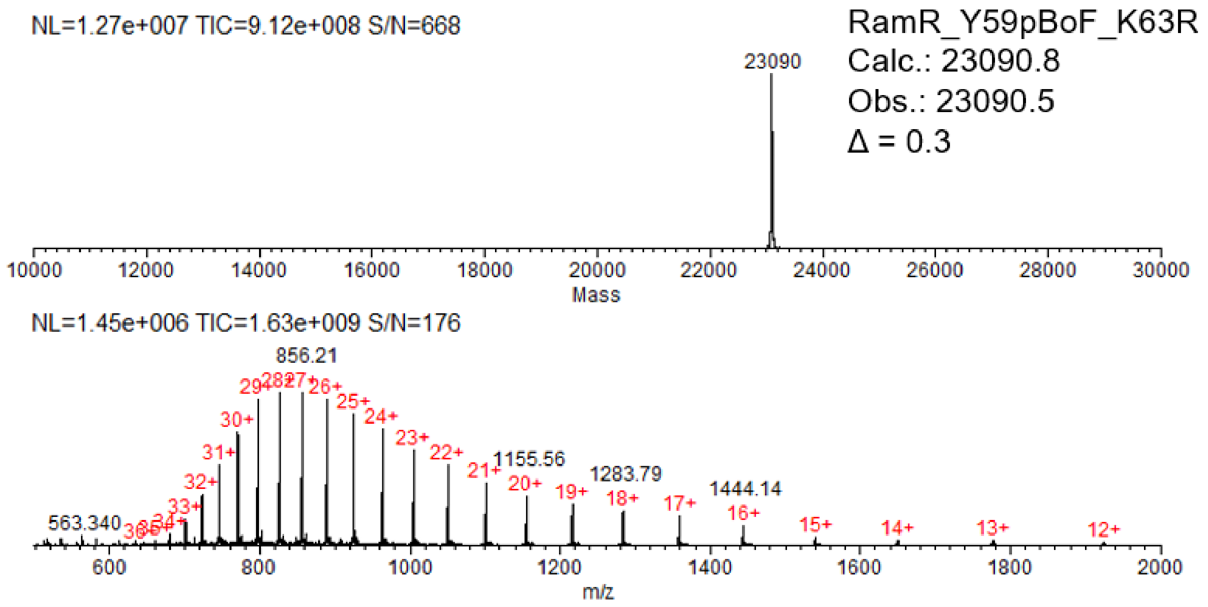
NL=1.27e+007 TIC=9.12e+008 S/N=668
23090
RamR_Y59pBoF_K63R
Calc.: 23090.8
Obs.: 23090.5
Δ = 0.3
Mass
NL=1.45e+006 TIC=1.63e+009 S/N=176
856.21
1155.56
1283.79
1444.14
563.340
m/z

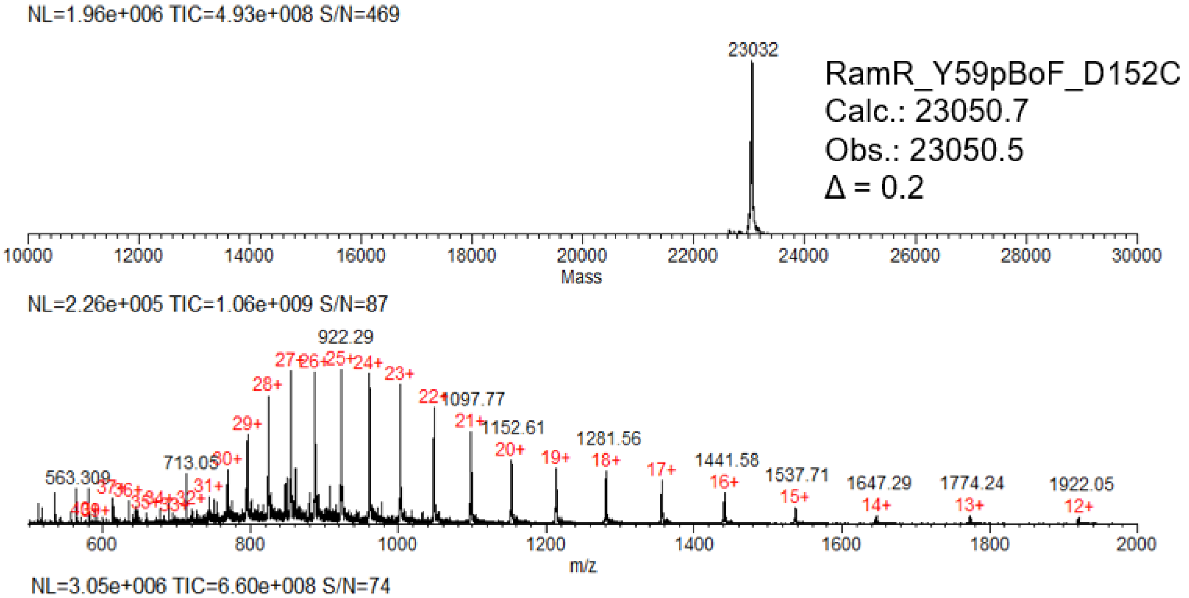
NL=1.96e+006 TIC=4.93e+008 S/N=469
23032
RamR_Y59pBoF_D152C
Calc.: 23050.7
Obs.: 23050.5
Δ = 0.2
Mass
NL=2.26e+005 TIC=1.06e+009 S/N=87
922.29
1097.77
1152.61
1281.56
1441.58
1537.71
1647.29
1774.24
1922.05
563.309
713.05
m/z

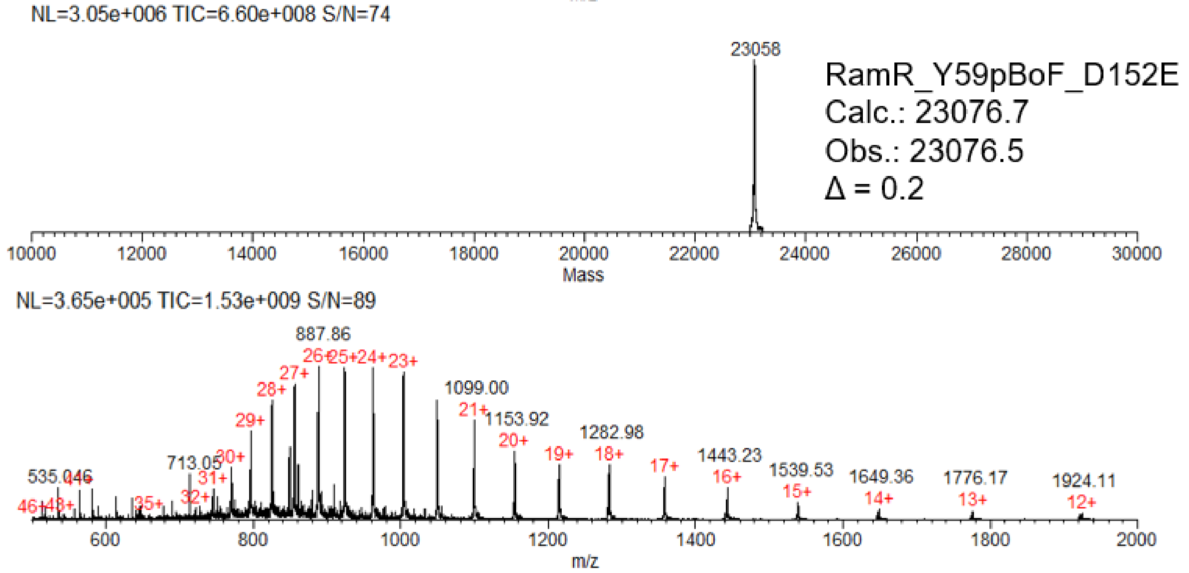
NL=3.05e+006 TIC=6.60e+008 S/N=74
23058
RamR_Y59pBoF_D152E
Calc.: 23076.7
Obs.: 23076.5
Δ = 0.2
Mass
NL=3.65e+005 TIC=1.53e+009 S/N=89
887.86
1099.00
1153.92
1282.98
1443.23
1539.53
1649.36
1776.17
1924.11
535.046
713.05
m/z

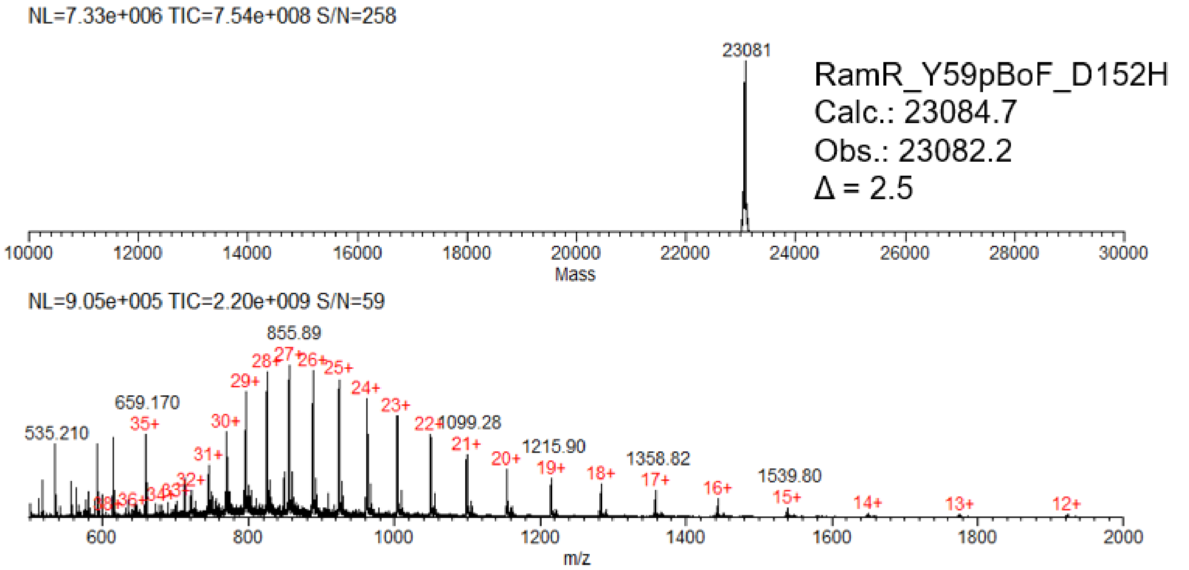
NL=7.33e+006 TIC=7.54e+008 S/N=258
23081
RamR_Y59pBoF_D152H
Calc.: 23084.7
Obs.: 23082.2
Δ = 2.5
Mass
NL=9.05e+005 TIC=2.20e+009 S/N=59
855.89
659.170
535.210
1099.28
1215.90
1358.82
1539.80
m/z

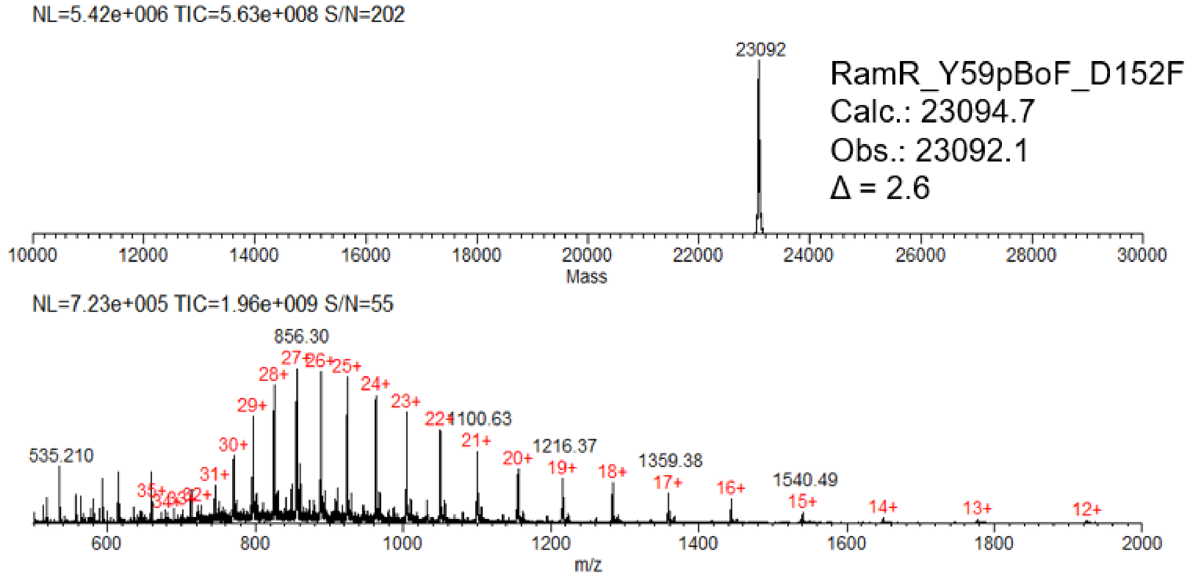
NL=5.42e+006 TIC=5.63e+008 S/N=202
23092
RamR_Y59pBoF_D152F
Calc.: 23094.7
Obs.: 23092.1
Δ = 2.6
Mass
NL=7.23e+005 TIC=1.96e+009 S/N=55
856.30
535.210
1100.63
1216.37
1359.38
1540.49
m/z

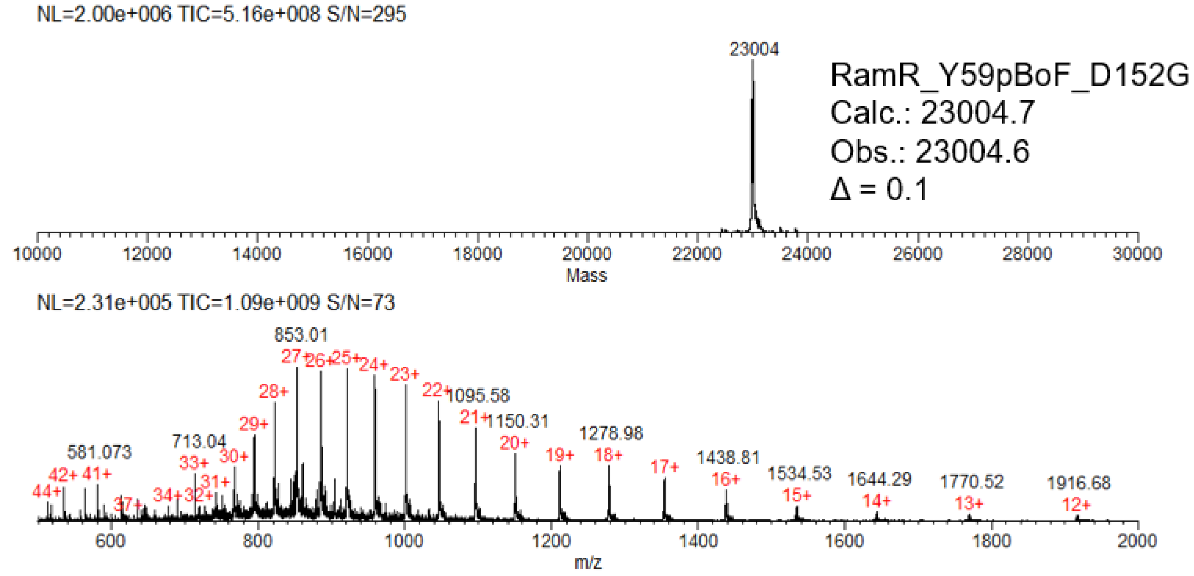
NL=2.00e+006 TIC=5.16e+008 S/N=295
23004
RamR_Y59pBoF_D152G
Calc.: 23004.7
Obs.: 23004.6
Δ = 0.1
Mass
NL=2.31e+005 TIC=1.09e+009 S/N=73
853.01
581.073
713.04
1095.58
1150.31
1278.98
1438.81
1534.53
1644.29
1770.52
1916.68
m/z

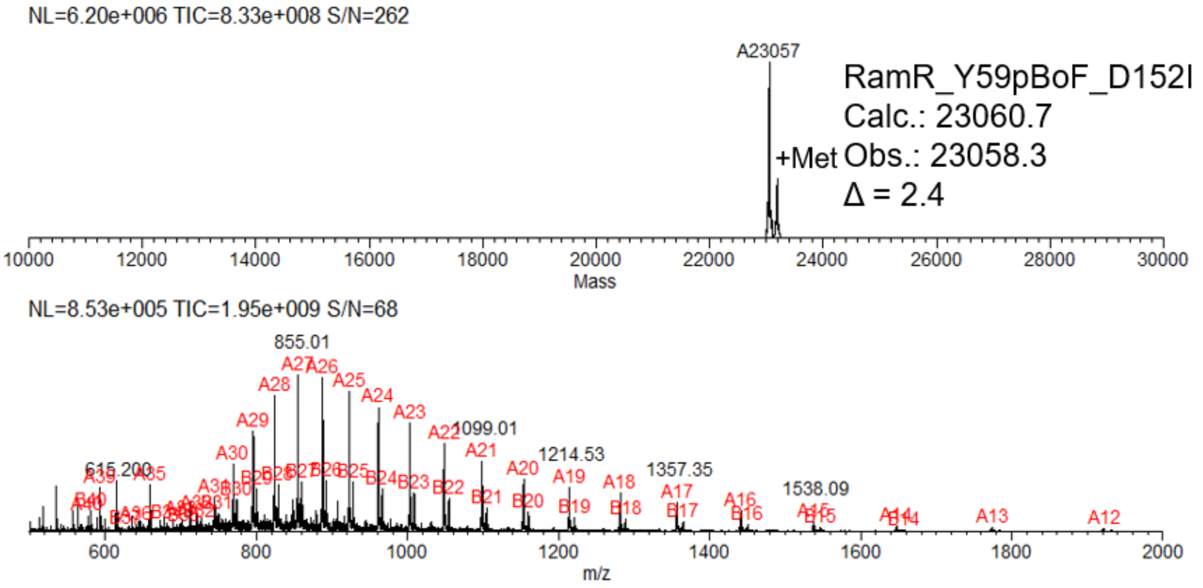
NL=6.20e+006 TIC=8.33e+008 S/N=262
A23057
RamR_Y59pBoF_D152I
Calc.: 23060.7
+Met
Obs.: 23058.3
Δ = 2.4
Mass
NL=8.53e+005 TIC=1.95e+009 S/N=68
855.01
1099.01
1214.53
1357.35
1538.09
m/z

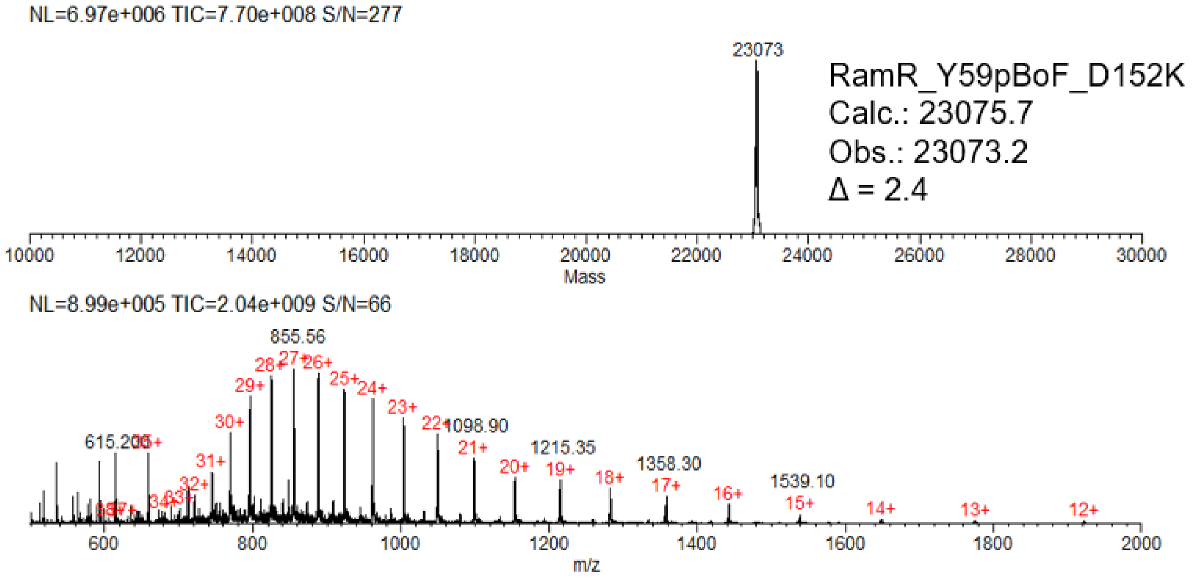
NL=6.97e+006 TIC=7.70e+008 S/N=277
23073
RamR_Y59pBoF_D152K
Calc.: 23075.7
Obs.: 23073.2
Δ = 2.4
Mass
NL=8.99e+005 TIC=2.04e+009 S/N=66
855.56
1098.90
1215.35
1358.30
1539.10
m/z

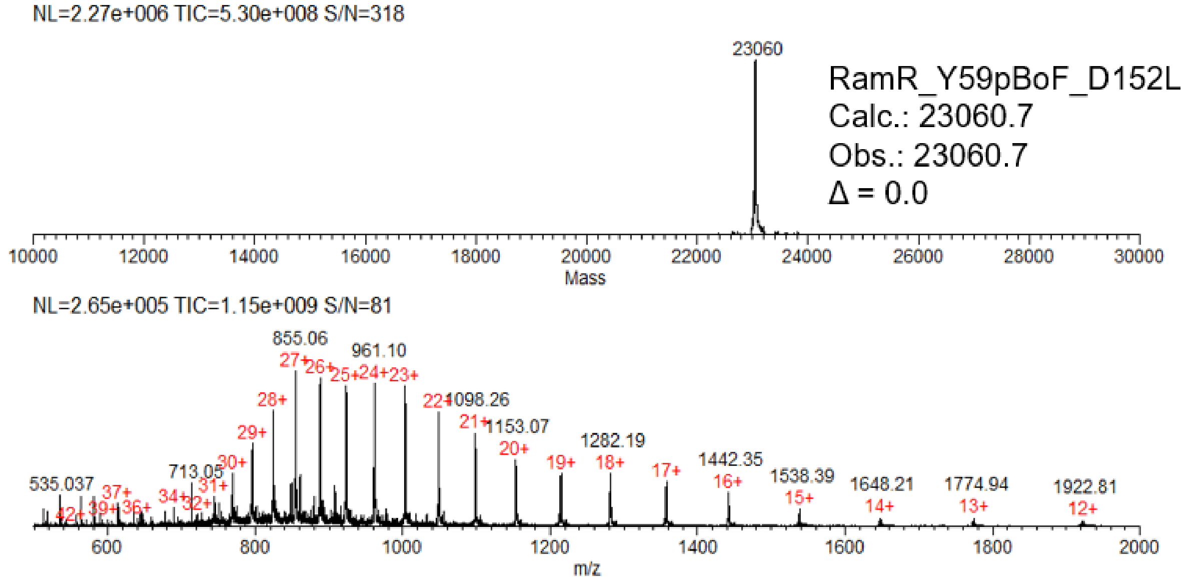
NL=2.27e+006 TIC=5.30e+008 S/N=318
23060
RamR_Y59pBoF_D152L
Calc.: 23060.7
Obs.: 23060.7
Δ = 0.0
Mass
NL=2.65e+005 TIC=1.15e+009 S/N=81
855.06
961.10
1098.26
1153.07
1282.19
1442.35
1538.39
1648.21
1774.94
1922.81
535.037
713.05
m/z

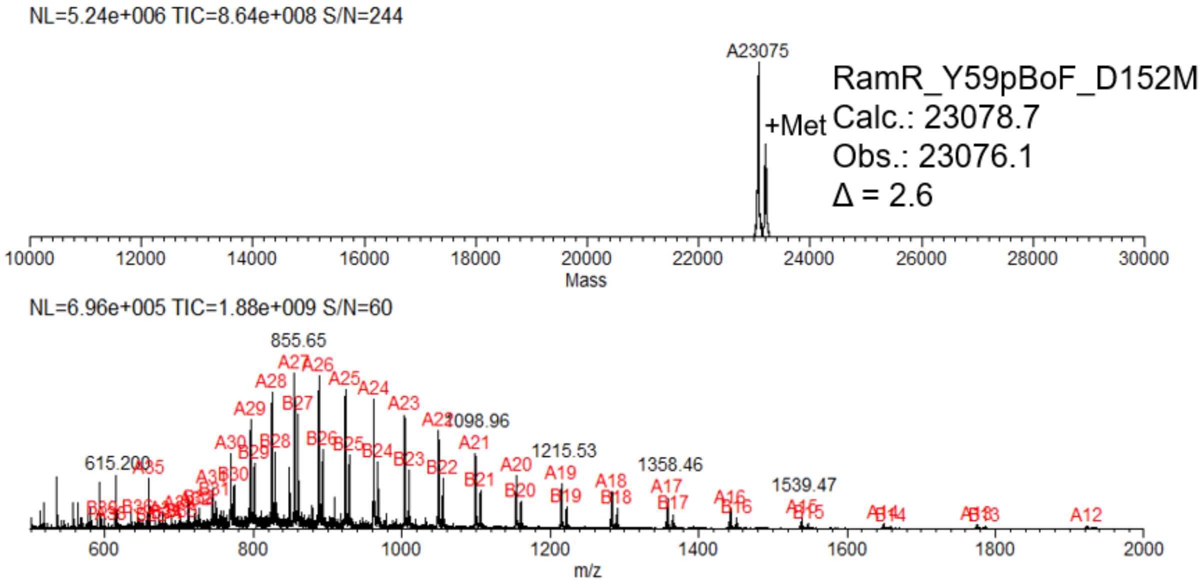
NL=5.24e+006 TIC=8.64e+008 S/N=244
A23075
+Met
RamR_Y59pBoF_D152M
Calc.: 23078.7
Obs.: 23076.1
Δ = 2.6
Mass
NL=6.96e+005 TIC=1.88e+009 S/N=60
855.65
1215.53
1358.46
1539.47
m/z

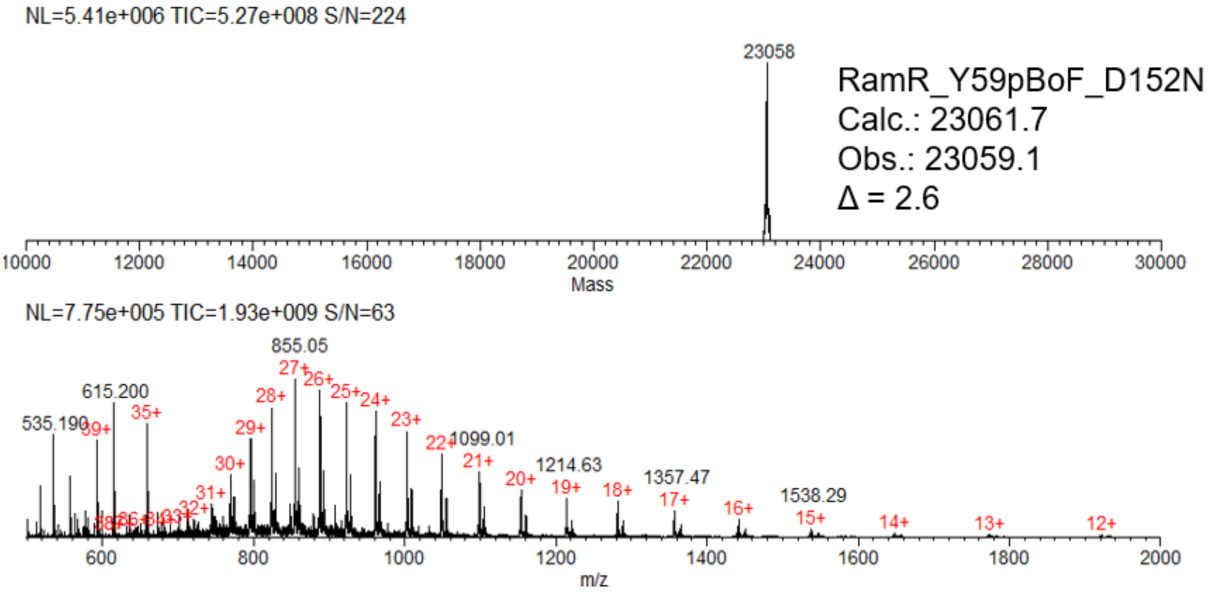
NL=5.41e+006 TIC=5.27e+008 S/N=224
23058
RamR_Y59pBoF_D152N
Calc.: 23061.7
Obs.: 23059.1
Δ = 2.6
Mass
NL=7.75e+005 TIC=1.93e+009 S/N=63
855.05
615.200
535.190
1099.01
1214.63
1357.47
1538.29
m/z

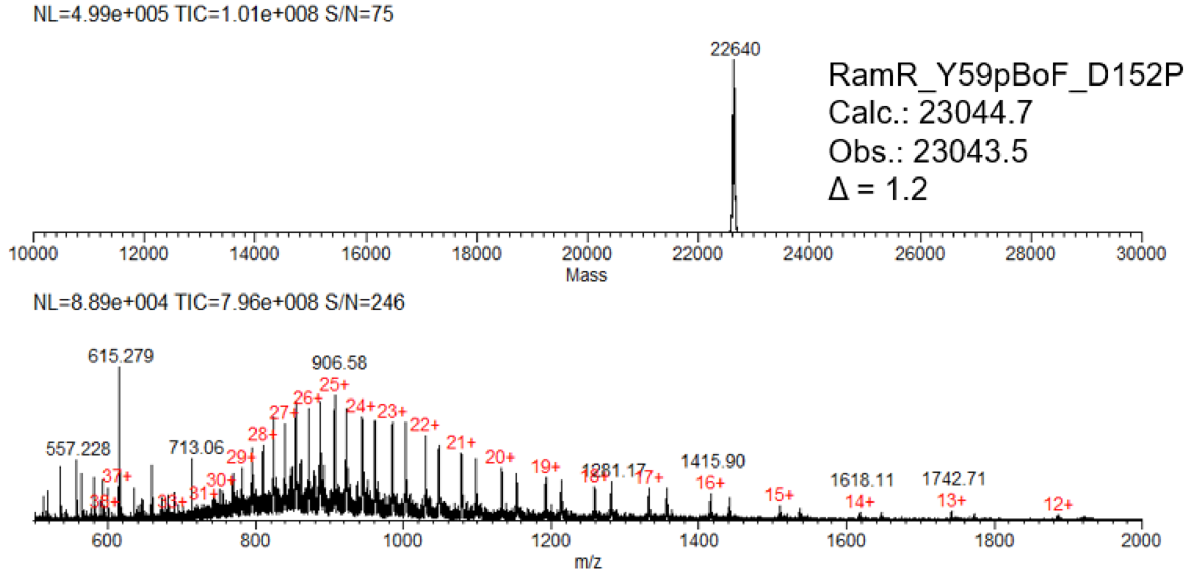
NL=4.99e+005 TIC=1.01e+008 S/N=75
22640
RamR_Y59pBoF_D152P
Calc.: 23044.7
Obs.: 23043.5
Δ = 1.2
Mass
NL=8.89e+004 TIC=7.96e+008 S/N=246
615.279
906.58
557.228
713.06
1415.90
1618.11
1742.71
m/z

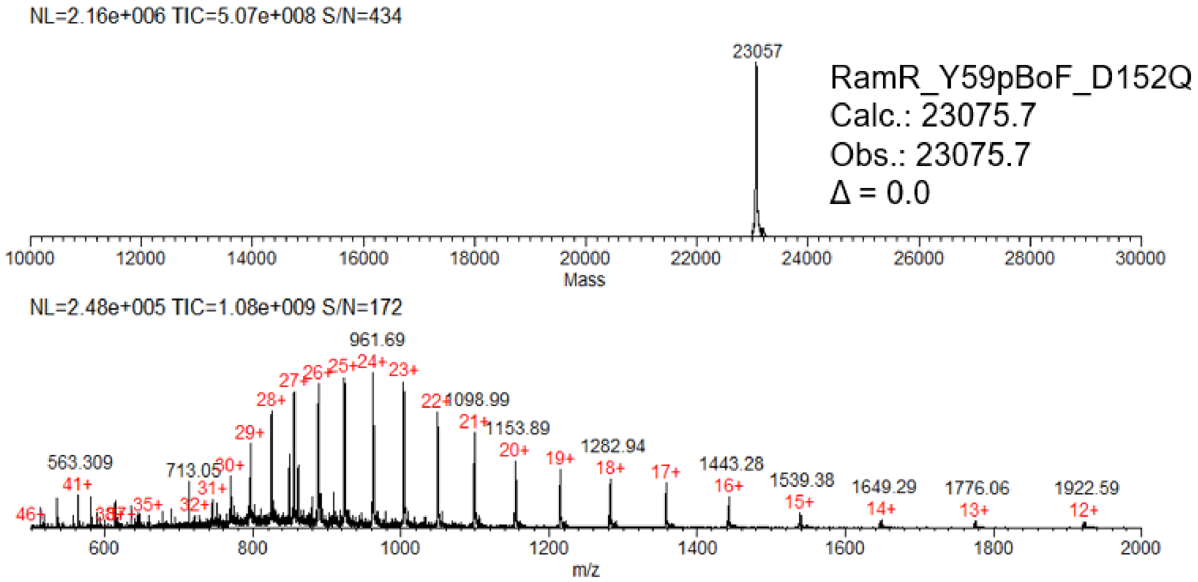
NL=2.16e+006 TIC=5.07e+008 S/N=434
23057
RamR_Y59pBoF_D152Q
Calc.: 23075.7
Obs.: 23075.7
Δ = 0.0
Mass
NL=2.48e+005 TIC=1.08e+009 S/N=172
961.69
1098.99
1153.89
1282.94
563.309
713.05
1443.28
1539.38
1649.29
1776.06
1922.59
m/z

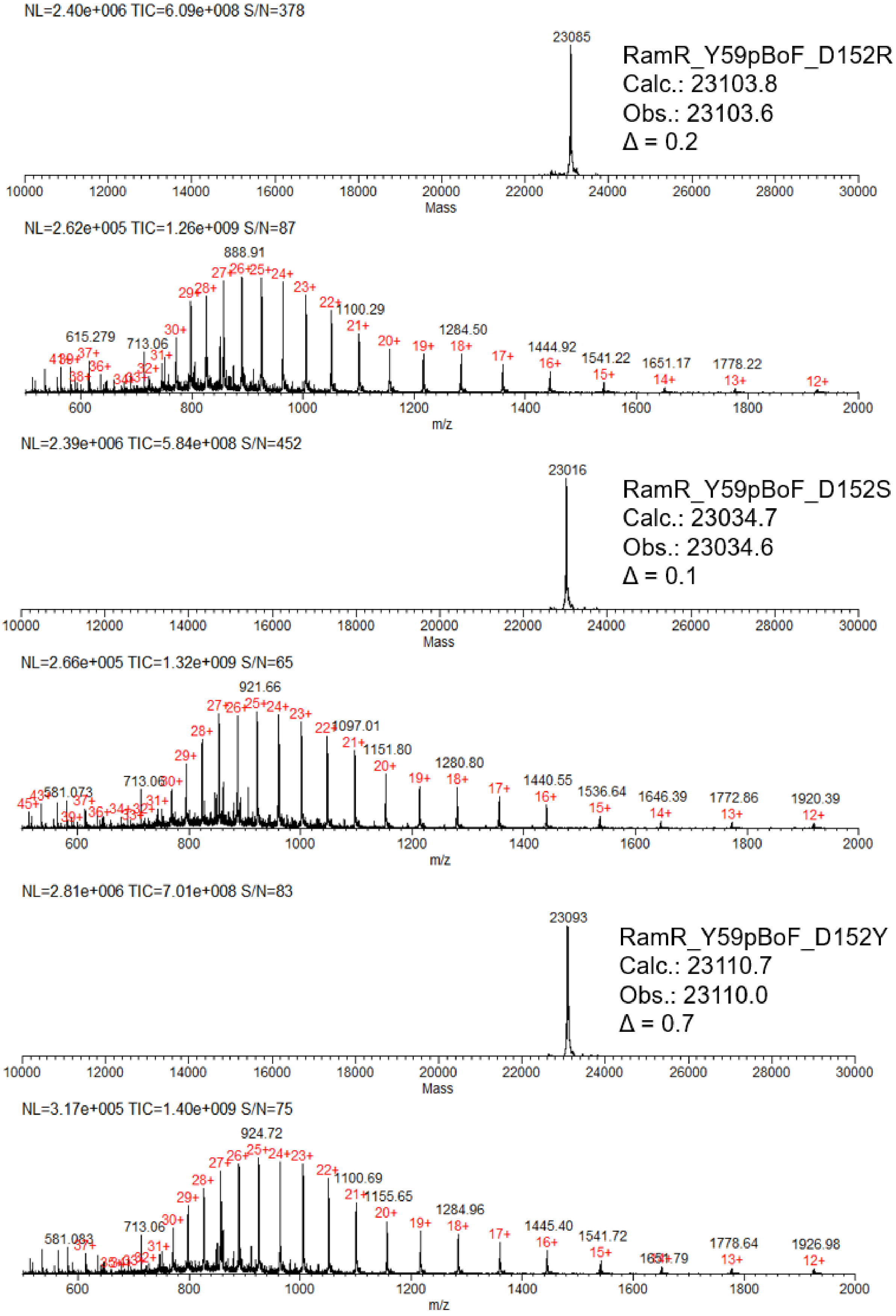
NL=2.40e+006 TIC=6.09e+008 S/N=378
23085
RamR_Y59pBoF_D152R
Calc.: 23103.8
Obs.: 23103.6
Δ = 0.2
Mass
NL=2.62e+005 TIC=1.26e+009 S/N=87
888.91
1100.29
1284.50
1444.92
1541.22
1651.17
1778.22
615.279
713.06
m/z
NL=2.39e+006 TIC=5.84e+008 S/N=452
23016
RamR_Y59pBoF_D152S
Calc.: 23034.7
Obs.: 23034.6
Δ = 0.1
Mass
NL=2.66e+005 TIC=1.32e+009 S/N=65
921.66
1097.01
1151.80
1280.80
1440.55
1536.64
1646.39
1772.86
1920.39
581.073
m/z
NL=2.81e+006 TIC=7.01e+008 S/N=83
23093
RamR_Y59pBoF_D152Y
Calc.: 23110.7
Obs.: 23110.0
Δ = 0.7
Mass
NL=3.17e+005 TIC=1.40e+009 S/N=75
924.72
1100.69
1155.65
1284.96
1445.40
1541.72
1778.64
1926.98
581.083
713.06
m/z

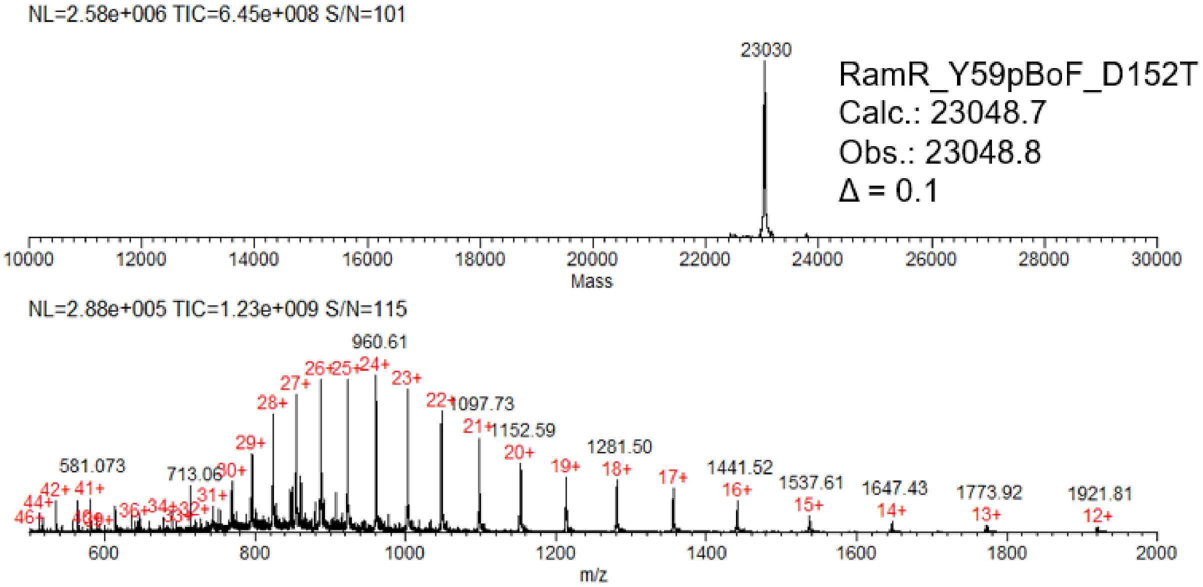
NL=2.58e+006 TIC=6.45e+008 S/N=101
23030
RamR_Y59pBoF_D152T
Calc.: 23048.7
Obs.: 23048.8
Δ = 0.1
Mass
NL=2.88e+005 TIC=1.23e+009 S/N=115
960.61
1097.73
1152.59
1281.50
1441.52
1537.61
1647.43
1773.92
1921.81
581.073
713.06
m/z

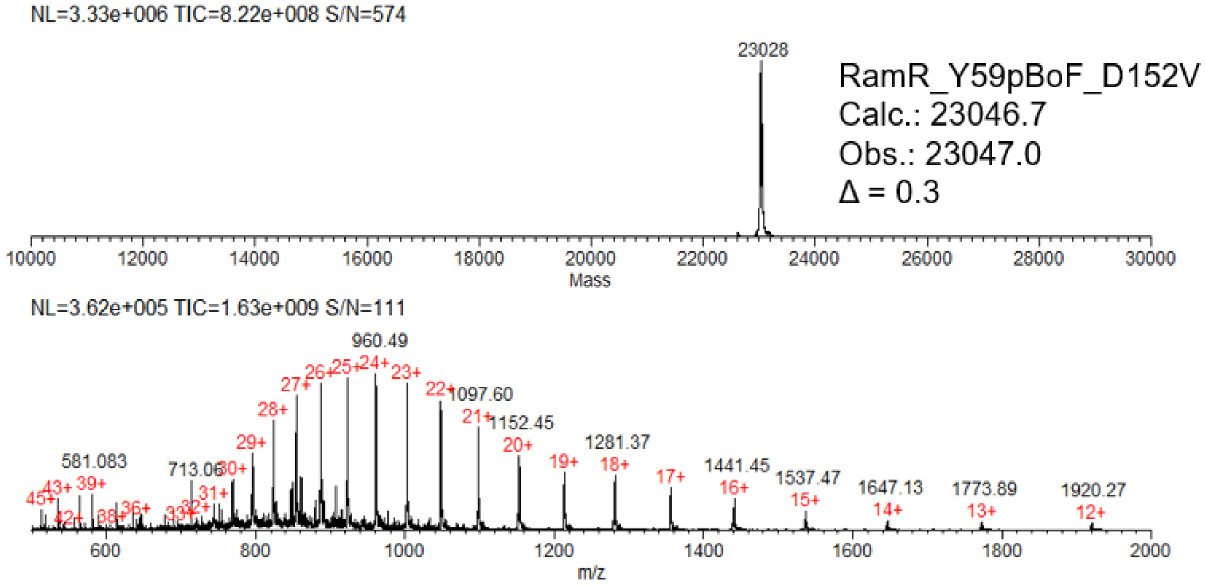
NL=3.33e+006 TIC=8.22e+008 S/N=574
23028
RamR_Y59pBoF_D152V
Calc.: 23046.7
Obs.: 23047.0
Δ = 0.3
Mass
NL=3.62e+005 TIC=1.63e+009 S/N=111
960.49
1097.60
1152.45
1281.37
1441.45
1537.47
1647.13
1773.89
1920.27
581.083
713.06
m/z

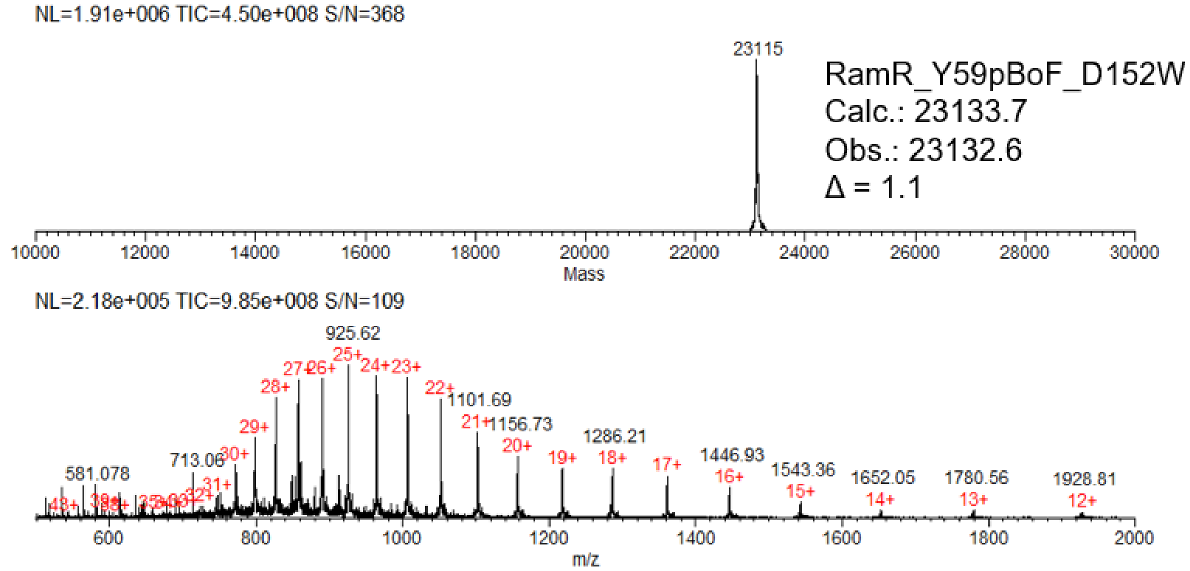
NL=1.91e+006 TIC=4.50e+008 S/N=368
23115
RamR_Y59pBoF_D152W
Calc.: 23133.7
Obs.: 23132.6
Δ = 1.1
Mass
NL=2.18e+005 TIC=9.85e+008 S/N=109
925.62
1101.69
1156.73
1286.21
1446.93
1543.36
1652.05
1780.56
1928.81
581.078
713.06
m/z

SI.18 References of supporting information

1. Straathof, A. J. J.; Jongejan, J. A., The enantiomeric ratio: origin, determination and prediction. *Enzyme Microb. Technol.* **1997,** *21* (8), 559-571.
2. Longwitz, L.; Leveson-Gower, R. B.; Rozeboom, H. J.; Thunnissen, A.-M. W. H.; Roelfes, G., Boron catalysis in a designer enzyme. *Nature* **2024,** *629* (8013), 824-829.
3. Macho, J. M.; Blue, R. M.; Lee, H.-W.; MacMillan, J. B., Boron NMR as a Method to Screen Natural Product Libraries for B-Containing Compounds. *Org. Lett.* **2022,** *24* (17), 3161-3166.
4. Brustad, E.; Bushey, M. L.; Lee, J. W.; Groff, D.; Liu, W.; Schultz, P. G., A Genetically Encoded Boronate-Containing Amino Acid. *Angew. Chem. Int. Ed.* **2008,** *47* (43), 8220-8223.
5. Svensson, O.; Malbet-Monaco, S.; Popov, A.; Nurizzo, D.; Bowler, M. W., Fully automatic characterization and data collection from crystals of biological macromolecules. *Acta Cryst. D* **2015,** *71* (8), 1757-1767.
6. Kabsch, W., XDS. *Acta Cryst. D* **2010,** *66* (2), 125-132.
7. Evans, P. R.; Murshudov, G. N., How good are my data and what is the resolution? *Acta Cryst. D* **2013,** *69* (7), 1204-1214.
8. Winn, M. D.; Ballard, C. C.; Cowtan, K. D.; Dodson, E. J.; Emsley, P.; Evans, P. R.; Keegan, R. M.; Krissinel, E. B.; Leslie, A. G. W.; McCoy, A.; McNicholas, S. J.; Murshudov, G. N.; Pannu, N. S.; Potterton, E. A.; Powell, H. R.; Read, R. J.; Vagin, A.; Wilson, K. S., Overview of the CCP4 suite and current developments. *Acta Cryst. D* **2011,** *67* (4), 235-242.
9. McCoy, A. J.; Grosse-Kunstleve, R. W.; Adams, P. D.; Winn, M. D.; Storoni, L. C.; Read, R. J., Phaser crystallographic software. *J. Appl. Crystallogr.* **2007,** *40* (4), 658-674.
10. Yamasaki, S.; Nikaido, E.; Nakashima, R.; Sakurai, K.; Fujiwara, D.; Fujii, I.; Nishino, K., The crystal structure of multidrug-resistance regulator RamR with multiple drugs. *Nat. Commun.* **2013,** *4* (1), 2078.
11. Emsley, P.; Lohkamp, B.; Scott, W. G.; Cowtan, K., Features and development of Coot. *Acta Cryst. D* **2010,** *66* (4), 486-501.
12. Murshudov, G. N.; Skubak, P.; Lebedev, A. A.; Pannu, N. S.; Steiner, R. A.; Nicholls, R. A.; Winn, M. D.; Long, F.; Vagin, A. A., REFMAC5 for the refinement of macromolecular crystal structures. *Acta Cryst. D* **2011,** *67* (4), 355-367.

13. Long, F.; Nicholls, R. A.; Emsley, P.; Grazulis, S.; Merkys, A.; Vaitkus, A.; Murshudov, G. N., AceDRG: a stereochemical description generator for ligands. *Acta Cryst. D* **2017,** *73* (2), 112-122.

14. Liebschner, D.; Afonine, P. V.; Baker, M. L.; Bunkoczi, G.; Chen, V. B.; Croll, T. I.; Hintze, B.; Hung, L.-W.; Jain, S.; McCoy, A. J.; Moriarty, N. W.; Oeffner, R. D.; Poon, B. K.; Prisant, M. G.; Read, R. J.; Richardson, J. S.; Richardson, D. C.; Sammito, M. D.; Sobolev, O. V.; Stockwell, D. H.; Terwilliger, T. C.; Urzhumtsev, A. G.; Videau, L. L.; Williams, C. J.; Adams, P. D., Macromolecular structure determination using X-rays, neutrons and electrons: recent developments in Phenix. *Acta Cryst. D* **2019,** *75* (10), 861-877.

15. Williams, C. J.; Headd, J. J.; Moriarty, N. W.; Prisant, M. G.; Videau, L. L.; Deis, L. N.; Verma, V.; Keedy, D. A.; Hintze, B. J.; Chen, V. B.; Jain, S.; Lewis, S. M.; Arendall III, W. B.; Snoeyink, J.; Adams, P. D.; Lovell, S. C.; Richardson, J. S.; Richardson, D. C., MolProbity: More and better reference data for improved all-atom structure validation. *Protein Sci.* **2018,** *27* (1), 293-315.

16. Veen MJ, A. F., Rozeboom HJ, Thunnissen A-MWH, Sauer DF, Roelfes G. A, A Genetically Encoded Thiophenol Recruits Noble Metals for Designer Enzymes. *ChemRxiv* **2024**.

17. Bersellini, M.; Roelfes, G., Multidrug resistance regulators (MDRs) as scaffolds for the design of artificial metalloenzymes. *Org. Biomol. Chem.* **2017,** *15* (14), 3069-3073.

18. Yang, Y.; Liu, M.; Wang, T.; Wang, Q.; Liu, H.; Xun, L.; Xia, Y., An Optimized Transformation Protocol for Escherichia coli BW3KD with Supreme DNA Assembly Efficiency. *Microbiol. Spectr.* **2022,** *10* (6), e02497-22.

19. Okuyama, T.; Nagamatsu, H.; Fueno, T., Mechanism of hydrolysis of hydroxy thiolesters in the presence of boric acid. *J. Org. Chem.* **1981,** *46* (7), 1336-1342.
